# Spatial profiling of cancer-associated fibroblasts of sporadic early onset colon cancer microenvironment

**DOI:** 10.1038/s41698-023-00474-w

**Published:** 2023-11-14

**Authors:** Satoru Furuhashi, Matias A. Bustos, Shodai Mizuno, Suyeon Ryu, Yalda Naeini, Anton J. Bilchik, Dave S. B. Hoon

**Affiliations:** 1 Department of Translational Molecular Medicine, Saint John’s Cancer Institute (SJCI), Providence Saint John’s Health Center (SJHC), Santa Monica, CA 90404 USA; 2 Department of Genome Sequencing Center, SJCI, Providence SJHC, Santa Monica, CA 90404 USA; 3 Department of Surgical Pathology, Providence SJHC, Santa Monica, CA 90404 USA; 4 Department of Gastrointestinal and Hepatobiliary Surgery, Providence SJHC, Santa Monica, CA 90404 USA

**Keywords:** Cancer microenvironment, Cancer microenvironment

## Abstract

The incidence of sporadic early-onset colon cancer (EOCC) has increased worldwide. The molecular mechanisms in the tumor and the tumor microenvironment (TME) in EOCC are not fully understood. The aim of this study is to unravel unique spatial transcriptomic and proteomic profiles in tumor epithelial cells and cancer-associated fibroblasts (CAFs). Here, we divide the sporadic colon cancer tissue samples with transcriptomic data into patients diagnosed with EOCC (<50 yrs) and late-onset colon cancer (LOCC, ≥50 yrs) and then, analyze the data using CIBERSORTx deconvolution software. EOCC tumors are more enriched in CAFs with fibroblast associated protein positive expression (FAP(+)) than LOCC tumors. EOCC patients with higher *FAP* mRNA levels in CAFs have shorter OS (Log-rank test, *p* < 0.029). Spatial transcriptomic analysis of 112 areas of interest, using NanoString GeoMx digital spatial profiling, demonstrate that FAP(+) CAFs at the EOCC tumor invasive margin show a significant upregulation of WNT signaling and higher mRNA/protein levels of fibroblast growth factor 20 (FGF20). Tumor epithelial cells at tumor invasive margin of EOCC tumors neighboring FAP(+) CAFs show significantly higher mRNA/protein levels of fibroblast growth factor receptor (FGFR2) and PI3K/Akt signaling activation. NichNET analysis show a potential interaction between FGF20 and FGFFR2. The role of FGF20 in activating FGFR2/pFGFR2 and AKT/pAKT was validated in-vitro. In conclusion, we identify a unique FAP(+) CAF population that showed WNT signaling upregulation and increased FGF20 levels; while neighbor tumor cells show the upregulation/activation of FGFR2-PI3K/Akt signaling at the tumor invasive margin of EOCC tumors.

## Introduction

There has been a decrease in the overall incidence and mortality of colon cancer worldwide since the mid-2000s^1–3^. However, the incidence of patients with early-onset colon cancer (EOCC), which are generally defined as patients <50 yrs, has significantly increased in the last decade^1–3^.

Patients with familial polyposis syndromes of the gastrointestinal tract, hereditary nonpolyposis colon cancer, Lynch syndrome, and inflammatory bowel disease are at increased risk of EOCC; however, those patients account for less than 20% of total colon cancer cases^4^. In other words, more than 80% of the newly diagnosed EOCC patients are considered sporadic cases, since they do not have microsatellite instabilities (MSI, a hypermutable phenotype) or germline mutations^5,6^. Past studies indicate that sporadic EOCC patients present with more left-sided colon cancer, advanced stage, and the tumors have poorer cell differentiation, are microsatellite stable, are chromosomal unstable, and have a higher frequency of mucinous cell histology than sporadic late-onset colon cancer (LOCC) patients^3,7,8^. Suggested risk factors for sporadic EOCC are gender (male), ethnicity (African American or Asian)^9^, and obesity^10^. Studies have sought to identify the actual etiologies and molecular mechanisms promoting sporadic EOCC, however, both remain elusive.

The inter- and intra-tumor heterogeneities at histological, genomic, epigenomic, and transcriptomic levels between EOCC and LOCC make it challenging to identify the detailed mechanisms driving sporadic EOCC^11,12^. The tumor microenvironment (TME) is essential in the pathogenesis of cancer, and the different cellular components of the TME play an important role in tumor progression, response to therapy, and prognosis^13^. Cancer-associated fibroblasts (CAFs) are one of the most prominent cell types in the TME with diverse phenotypes that are not well characterized^13^. CAFs are generally characterized by the expression of fibroblast activation protein (FAP) and actin alpha 2 (ACTA2), as well as other previously defined markers^13,14^. CAFs originate from resident normal fibroblasts and mesenchymal stem cells^15,16^. CAFs are responsible for the deposit and remodeling of the extracellular matrix (ECM) as well as the production and release of specific enzymes that contribute to the characteristics of the TME^17^. CAFs enhance epithelial cell growth, tumorigenicity, angiogenesis, and the metastatic potential of transformed cells^13^. Recent studies identify functionally distinct CAF subclasses based on gene expression in solid cancers^14,18,19^. Previous studies emphasize the importance of the spatial arrangement that promotes intercellular interactions between CAFs and malignant cells to generate more aggressive behavior as well as tumor resistance^20^.

In this study, we perform bioinformatic, proteomic, targeted RNA-Sequencing (RNA-Seq), and spatial transcriptome analysis using NanoString GeoMx digital spatial profiler (NGDSP, NanoString Technologies, Inc., Seattle, WA), to assess the tumor epithelial cells and CAFs in histologically defined regions of interest (ROI) including the tumor invasive margin, the tumor center, and the adjacent normal areas between EOCC and LOCC. The results suggest that specific cell transcriptomic changes in tumor epithelial cells and CAFs define specific cell states and signaling pathways at the tumor invasive margin of EOCC tumors.

## Results

### CAF-related genes are upregulated in the tumor tissues of EOCC patients

**Fig. 1:**
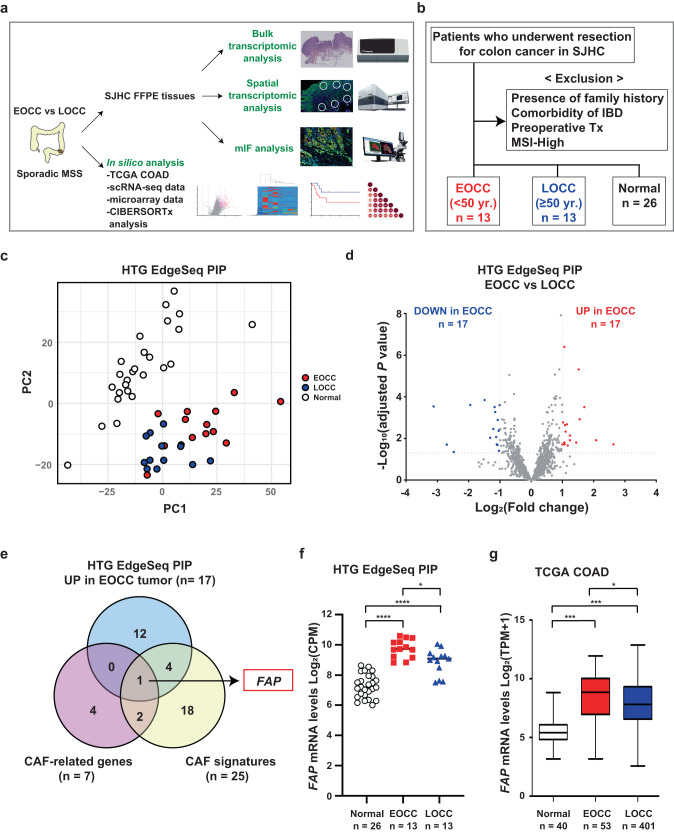
CAF-related gene markers are upregulated in sporadic MSS EOCC. **a** The schema of the study design includes the targeted sequencing, in silico, spatial transcriptomic, and mIF analysis. **b** Exclusion criteria of the 26 colon cancer patients selected at SJHC. Tissue samples were as follows EOCC (*n* = 13); LOCC (*n* = 13); adjacent normal tissue (*n* = 26). **c** PCA plot of the 52 FFPE tissue samples that were analyzed by HTG EdgeSeq PIP. **d** Volcano plot showing the DEGs in EOCC vs LOCC analyzed by HTG EdgeSeq PIP. **e** Venn diagram showing the overlapped genes that were upregulated in EOCC, CAF-related, and CAF-signatures. **f** Dot plot showing the mRNA levels of FAP (Log_2_ (CPM)) in adjacent normal (normal, white, (*n* = 26), EOCC (red, *n* = 13), and LOCC (blue, *n* = 13). **g** Box plot showing *FAP* mRNA levels (Log_2_(TPM + 1)) in normal tissues (white, *n* = 40), EOCC (red, *n* = 53), and LOCC (blue, *n* = 401). *p < 0.05; ***p < 0.001. EOCC early-onset colon cancer, LOCC late-onset colon cancer, MSS microsatellite stable, TCGA The Cancer Genome Atlas, CPM counts per million, COAD colon adenocarcinoma, scRNA-seq single cell RNA-sequencing, SJHC Saint John’s Health Center, FFPE formalin-fixed paraffin-embedded, mIF multiplex immunofluorescence, Tx treatment, MSI microsatellite instability, PIP precision immune panel, r correlation coefficient, and TPM transcripts per million.

In recent studies using single-cell RNA-seq (scRNA-Seq) different groups characterized the cell type abundance of the TME of colon cancer tumors^21^. However, there is limited information on transcriptomic profiles and the aberrant molecular signaling pathways in the TME of sporadic EOCC tumors that are distinctive from LOCC tumors. To evaluate this, several datasets have been utilized as described in Supplementary Table 1. Initially, 26 patients with a defined exclusion criteria were stratified based on the age of the patients into EOCC (<50 yrs, *n* = 13) and LOCC (≥50 yrs, *n* = 13, Supplementary Tables 1, 2). Additionally, 13 adjacent normal tissue samples were included as control for each EOCC and LOCC groups. Both EOCC and LOCC patients did not have significant differences in clinicopathological factors (Supplementary Table 3). Then, formalin-fixed-paraffin-embedded (FFPE) sections obtained from each patient were analyzed using targeted RNA-Seq assay that included 1,392 unique genes (HTG EdgeSeq Precision Immune Panel (PIP), Fig. 1a, b). Principal component analysis (PCA) plot showed that colon cancer samples from EOCC and LOCC clustered together compared to adjacent normal tissue samples (Fig. 1c). Transcriptomic analysis showed 34 significant differential expressed genes (DEG)s (Log_2_ Fold change (FC) |1| and adjusted *p* < 0.05) in EOCC tumor samples (Fig. 1d). Further analysis showed that among all the most common CAF-related genes (*FAP, ACTA2, COL11A1*, *ITGA11, CSPG4, TNC*, and *PDPN*)^22^ and CAF signatures^23^, *FAP* was the only consistent upregulated gene in EOCC compared to LOCC (Fig. 1d, f). These results made us hypothesize that CAFs may have some potential functions in EOCC.

Then, tumor samples from The Cancer Genome Atlas (TCGA) Colon Adenocarcinoma (COAD) from the RNA-seq dataset were stratified based on the age of the patients into EOCC (<50 yrs, *n* = 53) and LOCC (≥50 yrs, *n* = 401; Supplementary Fig. 1a–c). Both EOCC and LOCC patients did not have significant differences in clinicopathological factors (Supplementary Table 4). Transcriptomic analysis showed 597 significant DEGs (Log_2_FC | 0.5| and *p* < 0.05) in EOCC tumor samples (Supplementary Fig. 1d). To identify DEGs that may have biological implications in the TME of EOCC patients, we focused on seven genes (*FAP, ACTA2, COL11A1*, *ITGA11, CSPG4, TNC*, and *PDPN*) that are used to define CAF populations^22^. *FAP* and *ACTA2* showed the highest FC between EOCC and LOCC samples (Supplementary Fig. 1d) and showed strong positive correlations with the other CAF-related genes (Supplementary Fig. 1e). Furthermore, only *FAP*, *COL11A1*, and *ITGA11* were significantly upregulated in EOCC or LOCC tissues compared to normal colon tissues (Fig. 1g, and Supplementary Fig. 1f–k). In IHC analysis, ACTA2, but not FAP, was strongly detected in smooth muscle cells of colon cancer tumor tissues (Supplementary Fig. 3a–c and Supplementary Tables 5, 6), indicating the lack of cell specificity in ACTA2 to define CAFs populations in colon cancer.

At genomic level, there were no significant differences in the mutation frequency of the most frequently mutated oncogenes and tumor suppressor genes (*APC*, *KRAS*, *TP53*, *BRAF*, *NRAS*, and *PIK3CA*^24^); or in the proportion of the consensus molecular subtypes (CMS)^25^, Supplementary Table 4, Supplementary Fig. 2a–h).

To summarize, FAP is upregulated in EOCC tumors and detected in CAFs; thus, these results encourage us to characterize the CAFs FAP(+) population in EOCC tumors. We *hypothesized* that CAFs with FAP(+) expression best represented a distinctive cell population with biological implications in EOCC tumors.

### EPCAM(+) tumor epithelial cells and FAP(+) CAFs in EOCC have distinctive pathway enrichments

**Fig. 2:**
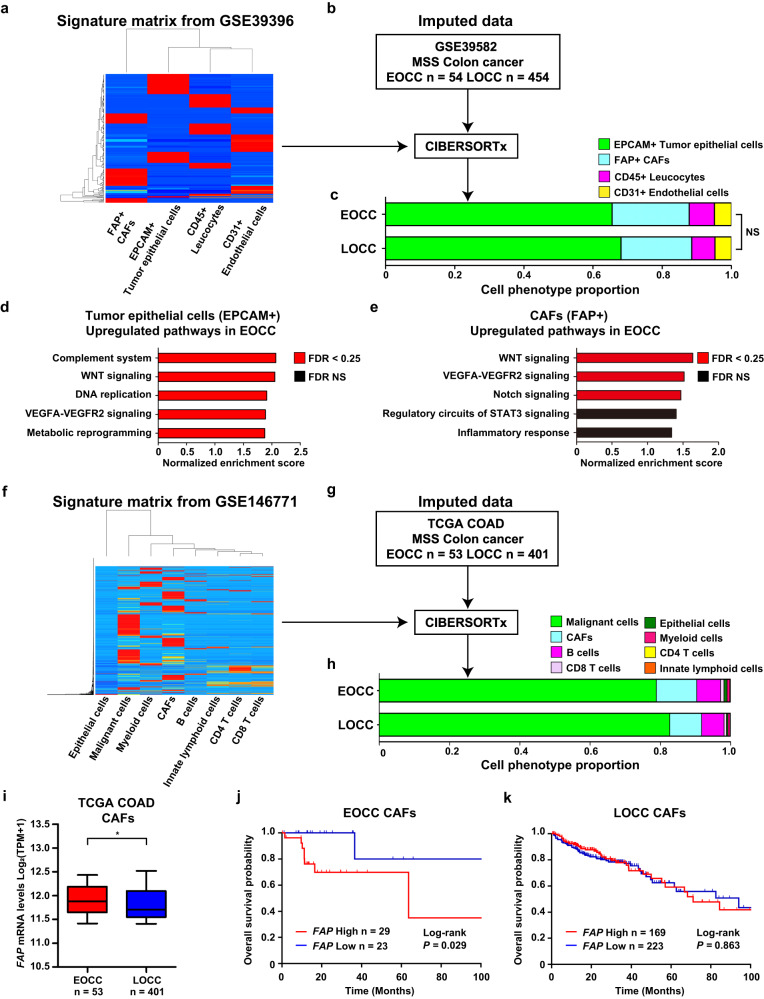
CIBERSORTx deconvolutes cell type abundance and expression from bulk transcriptomic data. **a, b** The workflow of CIBERSORTx. **a** Hierarchical clustering heatmap of the signature matrix created by CIBERSORTx using the GSE39396 dataset. **b** The GSE39582 dataset included MSS colon cancer tumors (*n* = 508) that were categorized into EOCC, (<50 yrs, *n* = 54) and LOCC (≥50 yrs, *n* = 454). The GSE39582 dataset was imputed with the signature matrix and deconvoluted by CIBERSORTx. **c** Stacked bar chart showing the average proportion of each cell phenotype in EOCC and LOCC samples. Bar chart showing normalized enrichment scores of the top five ranked upregulated pathways in EPCAM(+) tumor epithelial cells (**d**) or FAP(+) CAFs (**e**) in EOCC compared to LOCC. Red bars indicate significant *p*-values after considering an FDR < 0.25, and black bars indicate NS *p* values. **f** Hierarchical clustering heatmap of the signature matrix created by CIBERSORTx using the GSE146771 dataset. **g** TCGA COAD MSS colon cancer dataset was categorized into EOCC (*n* = 53) and LOCC (*n* = 401). The dataset was imputed with the signature matrix and deconvoluted by CIBERSORTx. **h** Stacked bar chart showing the average proportion of each cell phenotype in EOCC and LOCC samples. **i** Box plot showing estimated *FAP* mRNA levels (Log_2_(TPM + 1)) of EOCC (red, *n* = 53) and LOCC (blue, *n* = 401) in CAFs that were deconvoluted by CIBERSORTx. Kaplan-Meier survival curves of OS proportion in EOCC (**j**, *n* = 29 versus *n* = 23) and LOCC (**k**, *n* = 169 versus *n* = 223) patients. Patients were stratified on high or low *FAP* mRNA levels in CAFs by the minimum *p*-value approach. *p < 0.05. CAF cancer-associated fibroblast, MSS microsatellite stable, EOCC early-onset colon cancer, LOCC late-onset colon cancer, FDR false discovery rate, NS not significant, TCGA The Cancer Genome Atlas, COAD colon adenocarcinoma, TPM transcripts per million.

Previous studies identified the cell type abundances from RNA-Seq tissue analysis using CIBERSORTx software, which performs digital cytometry by data deconvolution^26^. Using this bioinformatic tool, we sought to investigate the transcriptomic profile of FAP(+) CAFs in the TME of EOCC tumors. CIBERSORTx requires a matrix to generate signatures and then, apply the matrix to specific datasets to estimate cell type abundances^26^. Colon cancer dataset GSE39396 was utilized to create a signature matrix (Supplementary Table 1). GSE39396 dataset contains cell data for the following cell populations EPCAM(+) tumor epithelial cells, FAP(+) CAFs, CD45(+) leucocytes, and CD31(+) endothelial cells (Fig. 2a). Then, the MSS colon cancer dataset GSE39582 (Supplementary Table 1) was imputed to CIBERSORTx using the signature matrix from GSE39396 (Fig. 2b). Using cell fractions mode, cell proportions of EPCAM(+) tumor epithelial cells, FAP(+) CAFs, CD45(+) leucocytes, and CD31(+) endothelial cells were generated for each tissue sample (Fig. 2c, and Supplementary Table 7). The average cellular proportion for each cell phenotype did not have any significant differences between EOCC and LOCC (Fig. 2c). Then, a gene expression profile for each cell phenotype in each patients’ sample was generated using CIBERSORTx high-resolution cell expression mode. The gene expression profiles in EPCAM(+) tumor epithelial cells were compared between EOCC and LOCC samples to determine distinctive pathways. Gene set enrichment analysis (GSEA) pathway analysis revealed that the complement system, WNT signaling, DNA replication, VEGFA-VEGFR2 signaling, and metabolic reprogramming were the top five ranked pathways in EPCAM(+) tumor epithelial cells of EOCC (Fig. 2d, Supplementary Table 8). In FAP(+) CAFs of EOCC tumor samples, the GSEA pathway analysis showed the upregulation of WNT signaling, VEGA-VEGFR2 pathway, and Notch signaling (Fig. 2e, Supplementary Table 9).

We also performed CIBERSORTx using the TCGA COAD dataset to validate our previous findings and evaluate the clinical impact of FAP(+) CAFs detected in the TME of EOCC on outcomes. The scRNA-seq data GSE146771 were utilized to create a signature matrix (Fig. 2f and Supplementary Table 1). Each cell phenotype proportion was successfully generated in both EOCC and LOCC tumors from TCGA COAD datasets (Fig. 2g, h, Supplementary Table 10). Consistently, the *FAP* mRNA levels were significantly higher in CAFs of EOCC than in LOCC tumors (*p* = 0.0142, Fig. 2i).

Then, colon cancer patients were stratified based on high-*FAP* or low-*FAP* mRNA levels in CAFs by using the minimum *p*-value approach^27^. Of clinical relevance, EOCC patients with high-*FAP* mRNA levels in CAFs had significantly shorter OS (*p* = 0.029, Fig. 2j), disease-specific survival (DSS, Supplementary Fig. 3d), and progression-free interval (PFI, Supplementary Fig. 3e) than EOCC patients with low-*FAP* mRNA levels. However, no significant differences were observed in OS, DSS, and PFI for LOCC patients with high- or low-*FAP* mRNA levels in CAFs (Fig. 2k, Supplementary Fig. 3f, g). To summarize, FAP(+) CAFs as well as EPCAM(+) tumor epithelial cells show an upregulation of WNT signaling. Furthermore, the presence of CAFs with high-*FAP* mRNA levels in the tumors was associated with a poor prognosis for EOCC patients.

### Unique CAFs population at the tumor invasive margin of EOCC tumors

**Fig. 3:**
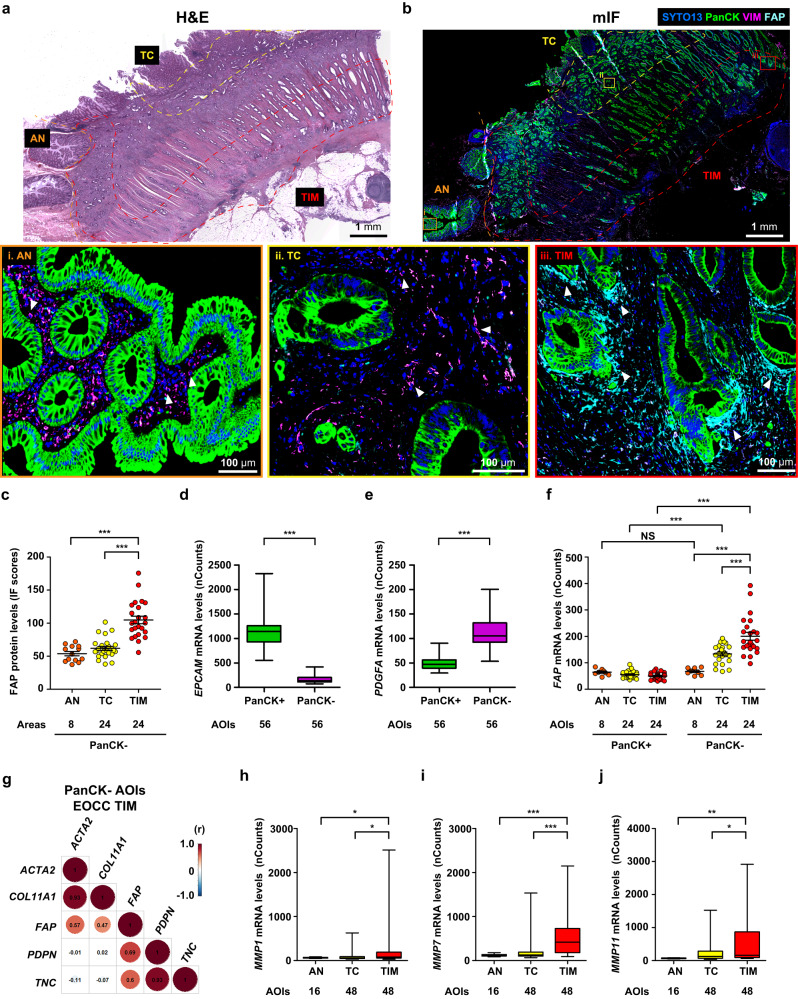
Multiplex immunofluorescence staining of the morphological markers utilized for GeoMx DSP analysis. **a** H&E staining of colon cancer samples. All the areas were marked with dotted lines: adjacent normal (AN, orange), tumor center (TC, yellow), and tumor invasive margin (TIM, red). **b** mIF staining using morphological markers including SYTO13 (DNA, blue), PanCK (green), VIM (magenta), and FAP (cyan) in sequential FFPE slides of (**a**). Representative images are shown for the AN (**i**), TC (**ii**), and TIM (**iii**). White arrowheads in images **i** and **ii** indicate VIM(+)/PanCK(−) cells. White arrowheads in image **iii** indicate FAP(+)/PanCK(−) cells. **c** Quantification of FAP protein levels in PanCK(-) area of AN (*n* = 8), TC (*n* = 24), and TIM (*n* = 24) using IF scores, which were calculated with Qupath software. Quantification of *EPCAM* (**d**) and *PDGFA* (**e**) mRNA levels (nCounts) in PanCK(+) (*n* = 56) and PanCK(-) (*n* = 56) AOIs using GeoMx DSP. **f** Quantification of *FAP* mRNA levels (nCounts) in PanCK(+) (AN (*n* = 8), TC (*n* = 24), and TIM (*n* = 24)) and PanCK(-) (AN (*n* = 8), TC (*n* = 24), and TIM (*n* = 24)) AOIs using GeoMx DSP. **g** Plot showing the Spearman correlation values for five CAF-related genes at EOCC TIM in PanCK- AOIs. Quantification of *MMP1* (**h**), *MMP7* (**i**), and *MMP11* (**j**) mRNA levels (nCounts) in AN (*n* = 16), TC (*n* = 48), and TIM (*n* = 48) AOIs obtained by GeoMx DSP analysis. *p < 0.05; **p < 0.01; ***p < 0.001. H&E hematoxylin and eosin, AN adjacent normal, TC tumor center, TIM tumor invasive margin, mIF multiplex immunofluorescence, PanCK pan-cytokeratin, VIM vimentin, IF immunofluorescence, nCounts normalized counts, NS not significant, AOI area of illumination, EOCC early-onset colon cancer, (r) correlation coefficient.

CAFs play critical roles in mutual crosstalk with cancer cells to promote tumor progression in various cancer types, including colon cancer^28^. Since scRNA-seq or the combination of bulk RNA-seq data and CIBERSORTx analysis lack spatial information, we utilized NGDSP to determine spatial cell arrangements and distribution of tumor epithelial cells and CAFs in FFPE tumor samples obtained from EOCC and LOCC patients. Four sporadic EOCC and four LOCC patients were selected based on matched clinicopathological information (Supplementary Table 2). The FFPE samples were stained by multiplex immunofluorescence (mIF) using four selected morphological markers (SYTO13, PanCK, VIM, and FAP). As we expected, PanCK(+) staining was limited to epithelial cells, while VIM(+) staining was broadly distributed in non-epithelial cells in both tumor and adjacent normal areas (Fig. 3a, b). FAP(+) staining was predominantly detected in non-epithelial cells, with a prevalence at tumor invasive margin (Fig. 3b). Quantitative analysis using Qupath software showed that FAP protein levels were significantly enhanced at PanCK(−) areas of tumor invasive margin compared to tumor center and adjacent normal areas (Fig. 3c, Supplementary Fig. 3h–j). Comparable results were observed in a validation cohort of colon cancer patients using the Opal mIF staining (Supplementary Fig. 4a–g). To summarize, mIF analysis demonstrated that FAP(+) CAFs are significantly increased at the tumor invasive margin of colon cancer.

### FAP(+) CAFs are enriched at the tumor-invasive margin

We performed spatial transcriptomic analysis using the NGDSP to gain insights into spatial relationships linking discrete cell states and potential intercellular interactions. Briefly, the ROIs were defined based on histological definition of adjacent normal, tumor center, and tumor invasive margin. In each ROI, specific areas of illumination (AOI)s were defined based on the morphological markers staining pattern. A total of 112 AOIs from four sporadic EOCC and LOCC patients were selected for analysis (Supplementary Fig. 5–8). *EPCAM* (a marker for epithelial cells) or *PDGFA* (a fibroblast marker)^22^ showed increased mRNA levels in PanCK(+) and PanCK(−) AOIs, respectively (Fig. 3d, e), suggesting that the transcriptomic data correlated with morphological markers.

Then, we investigated whether the mRNA profiles of PanCK(-) AOIs at EOCC tumor center and tumor invasive margin reflected the transcriptomic profiles found in CAF populations. Among the seven CAF-related genes (*FAP*, *ACTA2*, *COL11A1*, *ITGA11*, *CSPG4*, *TNC*, and *PDPN*) shown in Supplementary Fig. 1d, the cancer transcriptomic atlas (CTA) assay includes probes for *FAP*, *ACTA2*, *COL11A1*, *TNC*, and *PDPN*. The mRNA levels of *FAP*, *ACTA2*, *COL11A1*, *TNC*, and *PDPN* at tumor center and tumor invasive margin in PanCK(-) AOIs were significantly higher than in PanCK(+) AOIs (Fig. 3f, Supplementary Fig. 9a–d), suggesting that the transcriptomic profiles obtained from PanCK(-) AOIs were representative of CAF populations. Furthermore, the mRNA levels of *FAP* and *TNC* genes were significantly upregulated in PanCK(-) AOIs at the tumor invasive margin compared to the adjacent normal or tumor center (Fig. 3f, Supplementary Fig. 9c). The *FAP* mRNA levels showed a significant positive correlation with the *ACTA2*, *COL11A1*, *TNC*, and *PDPN* genes in PanCK(-) AOIs at EOCC tumor invasive margin (Fig. 3g). No positive correlations were observed for *ACTA2*, *COL11A1*, *TNC*, and *PDPN* genes in PanCK(-) AOIs at EOCC tumor center or at LOCC tumor invasive margin (Supplementary Fig. 9e, f). These results demonstrated that FAP(+) CAFs populations are enriched at EOCC tumor invasive margin.

Matrix metalloproteases (MMPs) belong to a protease group that control the tumor invasiveness and are often upregulated at the tumor invasive margin^29^. To demonstrate that the transcriptomic profiles during AOIs selection reflected the histological definition of tumor invasive margin, we evaluated the mRNA levels of *MMP1, MMP7*, and *MMP11* in tumor invasive margin, tumor center, and adjacent normal areas. All the MMPs analyzed were significantly upregulated at the tumor invasive margin compared to the tumor center and adjacent normal (Fig. 3h–j), supporting the histopathology definition of tumor invasive margin. Also, the mRNA levels of *CEACAM1* and *CEACAM6* (known membrane markers of malignant epithelial cells in various adenocarcinoma, including colon cancer^30,31^), as well as MKI67 (a proliferation marker), were significantly higher at tumor center and tumor invasive margin than adjacent normal in PanCK(+) AOIs (Supplementary Fig. 9g–i). In summary, the transcriptomic profiles of the selected AOIs strongly support the histopathology definition of tumor invasive margin, tumor center, and adjacent normal areas.

### Distinctive transcriptomic profiles of tumor invasive margin tumor epithelial cells and FAP(+) CAFs between EOCC and LOCC

**Fig. 4:**
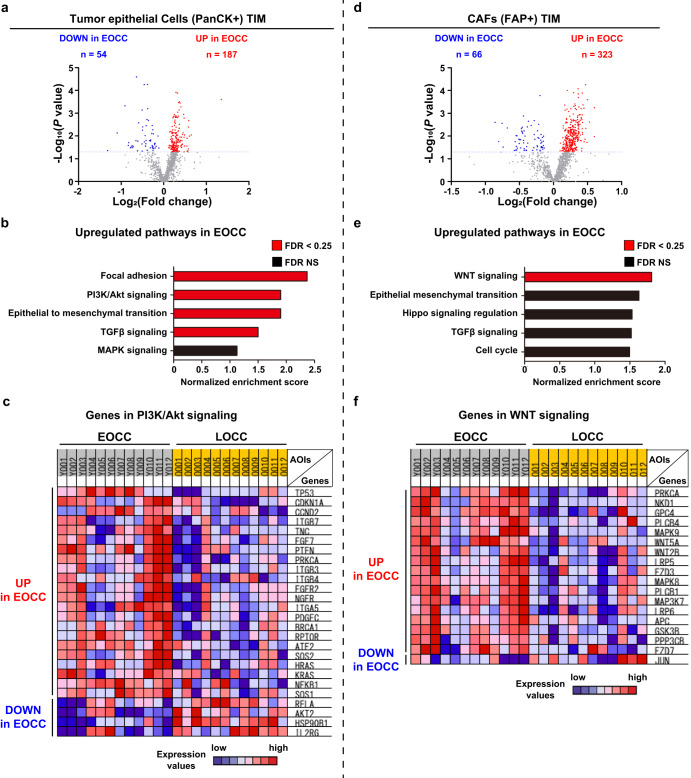
Distinct molecular profiles at the tumor invasive margin of PanCK(+) tumor epithelial cells and FAP(+) CAFs between EOCC and LOCC. **a** Volcano plot showing the DEGs in PanCK(+) tumor epithelial cells at tumor invasive margin (TIM) comparing between EOCC and LOCC obtained by NGDSP analysis. Of the 241 DEGs, 187 were upregulated (red dots) and 54 were downregulated (blue dots) in EOCC, respectively. **b** Bar chart showing normalized enrichment scores of top five ranked pathways that were upregulated in PanCK(+) AOIs of EOCC TIM compared to those of LOCC TIM. Red bars indicate significant *p*-values after considering FDR < 0.25 and black bars indicate NS *p*-values. **c** Heatmap comparing the mRNA levels of representative genes in the PI3K/Akt signaling pathway between PanCK(+) AOIs from EOCC and LOCC. **d** Volcano plot showing the DEGs in FAP(+) CAFs at TIM comparing between EOCC and LOCC obtained by GeoMx DSP. Of the 389 DEGs, 323 were upregulated (red dots) and 66 downregulated (blue dots) in EOCC. **e** Bar chart showing normalized enrichment scores of top five ranked pathways that were upregulated in FAP(+) AOIs of EOCC TIM compared to LOCC TIM. Red bars indicate significant *p*-values after considering an FDR < 0.25 and black bars indicate NS *p* values. **f** Heatmap comparing the mRNA levels of representative genes in the WNT signaling pathway between FAP(+) AOIs from EOCC and LOCC. TIM tumor invasive margin, EOCC early-onset colon cancer, FDR false discovery rate, NS not significant, AOI area of illumination.

We examined the DEGs in PanCK(+) tumor epithelial cells adjacent to FAP(+) CAFs areas at the tumor invasive margin between EOCC (*n* = 12 AOIs) and LOCC (*n* = 12 AOIs) using NGDSP analysis (Supplementary Fig. 5c, d). Of the 1,223 genes considered, 241 DEGs were found in EOCC compared to LOCC (Fig. 4a). GSEA analysis using the 241 DEGs showed that focal adhesion and the PI3K/Akt signaling were the most enriched pathways in EOCC PanCK(+) tumor epithelial cells (Fig. 4b, c). We also compared gene expression profiles of FAP(+) CAFs at tumor invasive margins between EOCC (*n* = 12 AOIs) and LOCC (*n* = 12 AOIs). A total of 389 DEGs were found in EOCC compared to LOCC tumors (Fig. 4d). Pathway analysis using the 389 DEGs showed that the WNT signaling pathway was the only significantly upregulated pathway (Fig. 4e, f).

To confirm that the above findings were specific to tumor invasive margin, we evaluated DEGs in each cell phenotype at tumor center between EOCC and LOCC. PanCK(+) tumor epithelial cells at tumor center showed 229 DEGs in EOCC compared to LOCC tumors (Supplementary Fig. 10a), however, pathway analysis showed no significant enrichment between EOCC and LOCC at tumor center (Supplementary Fig. 10b). In VIM(+) normal fibroblasts at tumor center, 152 DEGs were found in EOCC compared to LOCC tumors (Supplementary Fig. 10c), but pathway analysis showed no significant enrichment between EOCC and LOCC at tumor center (Supplementary Fig. 10d). The transcriptomic profiles of PanCK(+) epithelial cells or VIM(+) normal fibroblasts at adjacent normal were compared between EOCC and LOCC. The comparisons showed 53 DEGs in PanCK(+) epithelial cells and 55 DEGs in VIM(+) normal fibroblasts in EOCC tumors (Supplementary Fig. 10e, f). Pathway analysis using the DEGs in adjacent normal tissue between EOCC and LOCC showed no pathway enrichments due to limited number of DEGs. To summarize, the results indicated that PI3K/Akt and WNT signaling pathways were significantly upregulated at the EOCC tumor invasive margin in PanCK(+) tumor epithelial cells and FAP(+) CAFs, respectively. Thus, supporting the spatial transcriptomic changes at the tumor invasive margin of EOCC tumors.

### *FGF20*, a downstream target of the WNT signaling pathway in FAP(+) CAFs of EOCC

**Fig. 5:**
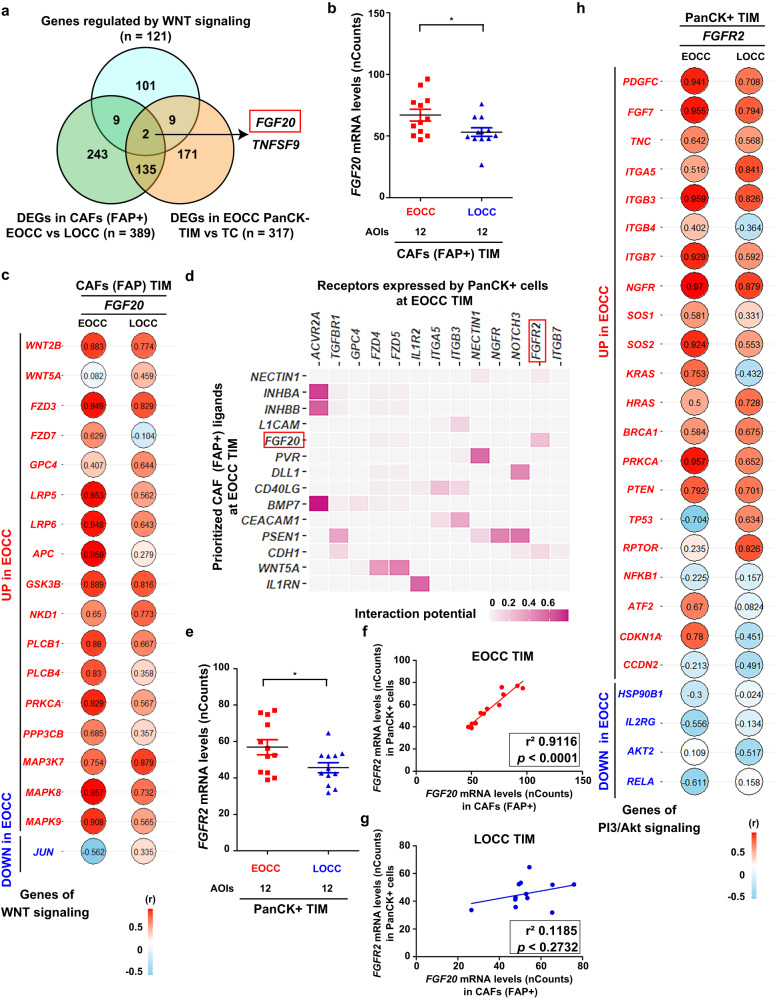
*FGF20* is upregulated in FAP(+) CAFs and *FGFR2* is upregulated in adjacent tumor epithelial cells with PI3K/Akt signaling upregulation. **a** Venn diagram showing the overlapping genes among the WNT pathway target genes (*n* = 121), the DEGs in FAP(+) CAFs at tumor invasive margin (TIM) between EOCC and LOCC (*n* = 389), and the DEGs in PanCK(-) cells between TIM and tumor center (TC) in EOCC (*n* = 317). **b** Scatter plot showing the *FGF20* mRNA levels in FAP(+) CAFs at TIM between EOCC (*n* = 12) and LOCC (*n* = 12). **c** Balloon plot indicating the Spearman correlation values among the mRNA levels of *FGF20* and those of the DEGs of the WNT signaling pathway in EOCC and LOCC, respectively. **d** Heatmap showing predicted ligand-receptor interactions between FAP(+) CAF and PanCK(+) tumor epithelial cells at EOCC TIM ordered by ligand activities according to NicheNet algorithm. **e** Scatter plot showing the *FGFR2* mRNA levels (nCounts) in tumor epithelial cells (PanCK+) at EOCC (*n* = 12) and LOCC (*n* = 12) TIM. Plot showing the correlation values between the mRNA levels (nCounts) of *FGF20* in FAP(+) CAFs and *FGFR2* in PanCK(+) tumor epithelial cells at EOCC TIM (**f**) and LOCC TIM (**g**), respectively. **h** Balloon plot indicating the spearman correlation values between the mRNA levels of *FGFR2* and those of the DEGs of PI3K/Akt signaling pathway in EOCC and LOCC, respectively. *p < 0.05. DEGs differentially expressed genes, CAF cancer-associated fibroblast, EOCC early-onset colon cancer, LOCC late-onset colon cancer, TIM tumor invasive margin, TC tumor center, nCounts normalized counts, (r) correlation coefficient, r^2^ coefficient of determination.

Based on the PI3K/Akt and WNT pathways upregulation observed at the EOCC tumor invasive margin using spatial analysis, we sought to identify potential intercellular crosstalk between PanCK(+) tumor epithelial cells and FAP(+) CAFs at the EOCC tumor invasive margin. To determine the downstream target genes of WNT signaling that were upregulated in FAP(+) CAFs of EOCC (Fig. 4e, f), we searched for WNT signaling target genes (https://web.stanford.edu/group/nusselab/cgi-bin/wnt/target_genes). Of the 121 candidate genes listed in the website database (Supplementary Table 11), two genes (fibroblast growth factor 20*; FGF20* and tumor necrosis factor superfamily member 9; *TNFSF9*) overlapped with DEGs in FAP(+) CAFs at the tumor invasive margin between EOCC and LOCC tumors, and with the DEGs in PanCK(-) AOIs between the tumor invasive margin and tumor center in EOCC tumors (Fig. 5a). These results suggested that both *FGF20* and *TNFSF9* showed a spatial upregulation at the tumor invasive margin of EOCC.

Previous studies demonstrated the upregulation of *FGF20* by WNT signaling activation in CRC and other cell lines^32,33^. *FGF20* is secreted and functions in a paracrine manner to bind and activate FGF receptors^34^. Thus, for further analysis we focused on *FGF20* as a WNT signaling downstream target*. FGF20* mRNA levels were significantly upregulated in FAP(+) CAFs at the tumor invasive margin of EOCC compared to LOCC tumors (Fig. 5b) and showed a significant positive correlation (*r* > 0.6) with 15 of 17 DEGs of WNT signaling in EOCC (Fig. 5c). These results indicated a strong association between the WNT signaling and the upregulation of *FGF20* mRNA levels in FAP(+) CAFs at the EOCC tumor invasive margin. Thus, we *hypothesized* that the focal increased levels of FGF20 in FAP(+) CAFs affect the neighbor tumor epithelial cells at the EOCC tumor invasive margin.

### Upregulated *FGFR2* in tumor epithelial cells in EOCC tumor invasive margin

We sought to determine how the upregulation of *FGF20*, a downstream effector of WNT signaling in FAP(+) CAFs, can potentially influence neighbor PanCK(+) tumor epithelial cells at EOCC tumor invasive margin. NicheNet analyses^35^ were applied to predict differentially expressed ligands in FAP(+) CAFs that would interact with receptors at neighbor PanCK(+) tumor epithelial cells at EOCC tumor invasive margin. Potential ligand-receptor interaction analysis showed that FGF20 ligand produced in FAP(+) CAFs at EOCC tumor invasive margin had the highest interaction potential to bind to FGFR2 in PanCK(+) tumor epithelial cells at EOCC tumor invasive margin (Fig. 5d).

Among the FGFR family members (*FGFR1*, *FGFR2*, *FGFR3*, and *FGFR4*), only *FGFR2* was significantly upregulated in PanCK(+) tumor epithelial cells at the EOCC tumor invasive margin compared to the LOCC tumor invasive margin in NGDSP analysis (Fig. 5e, Supplementary Fig. 11a). Intriguingly, the *FGFR2* mRNA levels in PanCK(+) tumor epithelial cells at the EOCC tumor invasive margin were positively correlated with *FGF20* mRNA levels in neighbor FAP(+) CAFs at the EOCC tumor invasive margin (Fig. 5f); while no correlations between *FGFR2* and *FGF20* were observed in LOCC tumor invasive margin (Fig. 5g).

In addition, we evaluated the DEGs between PanCK(+) and FAP(+) in EOCC compared to LOCC at tumor invasive margin, tumor center, or adjacent normal areas. The results showed the overlap of 123, 50, and 9 DEGs in each comparison (Supplementary Fig. 12a–c, Supplementary Table 12). Of notice, FGFR2 was also detected as upregulated in FAP(+) stromal cells at the tumor invasive margin of EOCC (Supplementary Fig. 12d).

Since FGF-FGFR interaction activates the PI3K/Akt signaling pathway^36^, the correlations between *FGFR2* and PI3K/Akt signaling pathway genes were investigated. The *FGFR2* mRNA levels positively correlated with all the PI3K/Akt signaling pathway genes analyzed, except for *TP53*, *NFKB1*, and *CCDN2* (Fig. 5h). These findings suggested that FGFR2 in tumor epithelial cells may function as a receptor for the secreted FGF20. FGFR2 is upregulated in tumor epithelial cells, while FGF20 is increased in neighbor FAP(+) CAFs at EOCC tumor invasive margin.

The cell cycle is regulated by cyclin-dependent kinase (CDK)-Cyclin complex, which are activated by the PI3K/Akt signaling^37^. Surprisingly, the mRNA levels of *CDK1* and *Cyclin A2* (*CCNA2*) were significantly higher in the tumor epithelial cells of EOCC tumor invasive margin compared to LOCC tumor invasive margin. No significant differences were observed for *CDK2* and *Cyclin B1* (*CCNB1*; Supplementary Fig. 11b). In addition, the mRNA levels of *CDK1* showed significant positive correlations with *FGF20* in FAP(+) CAFs and *FGFR2* in PanCK(+) tumor epithelial cells at EOCC tumor invasive margin, but not at LOCC tumor invasive margin (Supplementary Fig. 11c–f). These data suggest that *CDK1* and *CCNA2* genes, which are downstream of the PI3K/Akt signaling pathway, are upregulated in tumor epithelial cells at EOCC tumor invasive margin.

### Interactions between FAP(+) CAFs and PanCK(+) tumor epithelial cells at EOCC tumor invasive margin through FGF20-FGFR2 axis

**Fig. 6:**
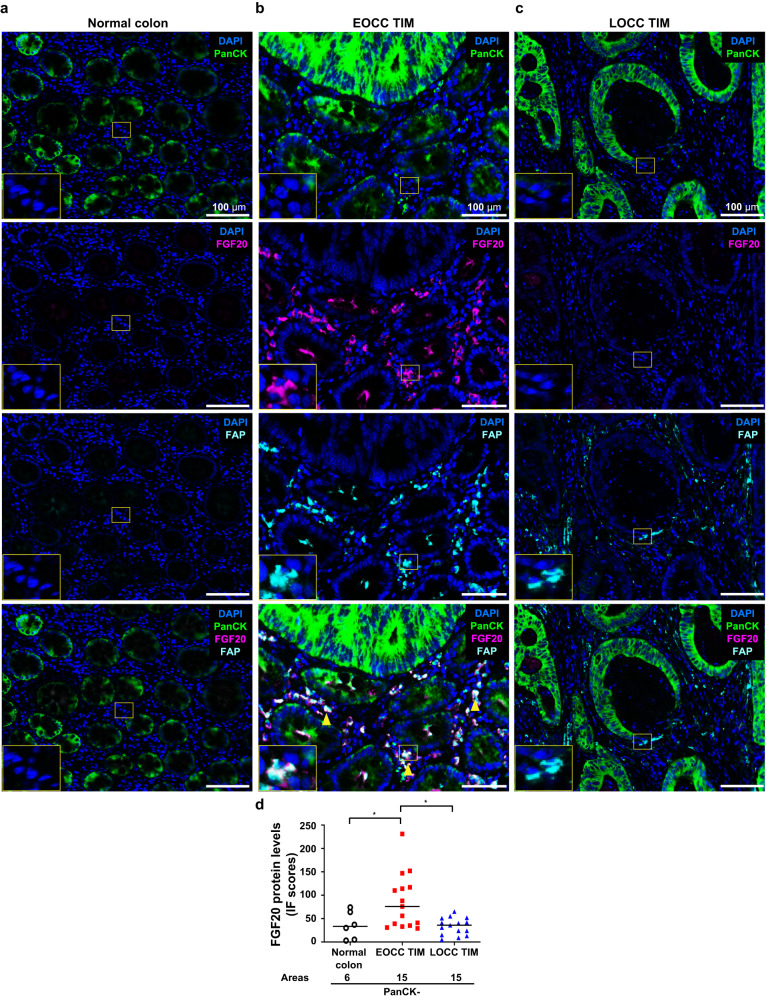
Multiplex immunofluorescences show FGF20 staining patterns at stromal tissues of EOCC tumor invasive margin. Representative mIF images of normal colon (**a**), EOCC tumor invasive margin (TIM, **b**), and LOCC TIM (**c**). Tissue samples were stained using the Opal multiplex staining kit. DAPI (blue); PanCK (green, Opal 690); FGF20 (magenta, Opal 540); FAP (cyan, Opal 650). Yellow arrowheads in the middle bottom picture indicate PanCK(-) cells with positive staining for both FAP and FGF20 proteins. The insets represent the magnified image of the yellow box of each picture. **d** Quantification of FGF20 protein levels in each area obtained from adjacent normal colon (white, *n* = 6), EOCC (red, *n* = 15), and LOCC (blue, *n* = 15) tissues using IF scores that were calculated with InForm software. *p < 0.05. EOCC early-onset colon cancer, TIM tumor invasive margin, LOCC late-onset colon cancer, mIF multiplex immunofluorescence.

**Fig. 7:**
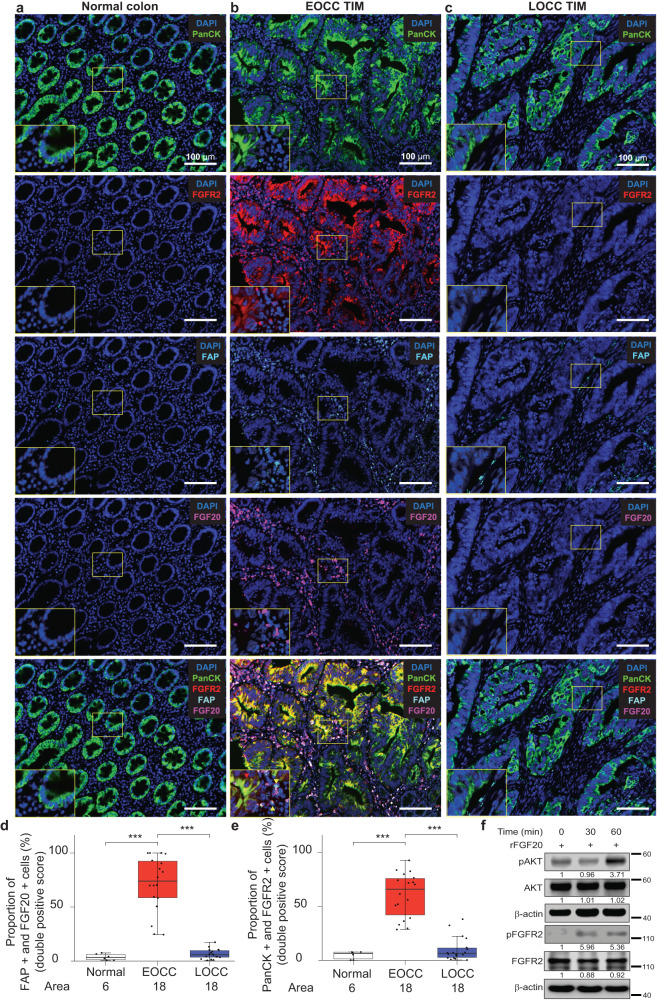
Multiplex immunofluorescences show FGFR2 and FGF20 staining patterns at tumor epithelial cells of EOCC tumor invasive margin. Representative mIF images of normal colon (**a**), EOCC tumor invasive margin (TIM, **b**) and LOCC TIM (**c**). Tissue samples were stained using the Opal kit: DAPI (blue), PanCK (green, Opal 690); FGFR2 (red, Opal 620); FAP (cyan, Opal 650); FGF20 (magenta, Opal 540). The insets represent the magnified image of the yellow box of each picture. **d** Quantification of FGF20(+)/FAP(+) cells in normal colon (white, *n* = 6), EOCC (red, *n* = 18), and LOCC (blue, *n* = 18) tissue areas. The percentage of FGF20(+) cells that were FAP(+) was estimated using QuPath software. **e** Quantification of FGFR2(+)/PanCK(+) cells in normal colon (white, *n* = 6), EOCC (red, *n* = 18), and LOCC (blue, *n* = 18) tissue areas. The percentage of FGFR2(+) cells that were PanCK(+) were estimated using QuPath software. **f** Western blot analysis for phosphorylated-FGFR2 (pFGFR2), FGFR2, phosphorylated-AKT (pAKT), AKT, β-actin in HT-29 cell lines that were incubated with recombinant FGF20 (rFGF20) for 30 and 60 min. β-actin was used as loading control. ***p < 0.001. EOCC early-onset colon cancer, TIM tumor invasive margin, LOCC late-onset colon cancer, mIF multiplex immunofluorescence.

To validate our previous observations obtained from NGDSP spatial analysis, the FGF20 and FGFR2 protein levels and distribution at the EOCC tumor invasive margin were evaluated by mIF. Significantly higher FGF20 protein levels were observed in the PanCK(-) areas at the EOCC tumor invasive margin than in LOCC tumor invasive margin and normal colon tissue areas (Fig. 6a–d). In addition, FGF20 protein detection overlapped with FAP protein in PanCK(-) areas at EOCC tumor invasive margin (Fig. 6b). Quantitative analysis for FGFR2 revealed higher FGFR2 protein levels in tumor epithelial cells at tumor invasive margin of EOCC than LOCC or normal colon tissue areas (Supplementary Fig. 13); on contrary the protein levels of FGF20 were significantly upregulated in CAFs FAP(+) at the tumor invasive margin of EOCC compared to LOCC (Fig. 7a–d and Supplementary Fig. 14).

Then, to reinforce our findings, functional assays were performed in HT-29 cell line (derived from a primary tumor obtained from an EOCC patient). Briefly, HT-29 cell line was incubated with 10 ng/mL of recombinant FGF20 (rFGF20) at different time points. As readout, the protein levels of phosphorylated-FGFR2 and total FGFR2 were measured. rFGF20 induced the activation of pFGFR2 at 30 and 60 min compared to control (Fig. 7f). Also, the levels of pAKT/AKT were measured. Consistently, the pAKT levels increased at 60 min after incubation with rFGF20 (Fig. 7f), suggesting an AKT-activation mediated by rFGF20.

**Fig. 8:**
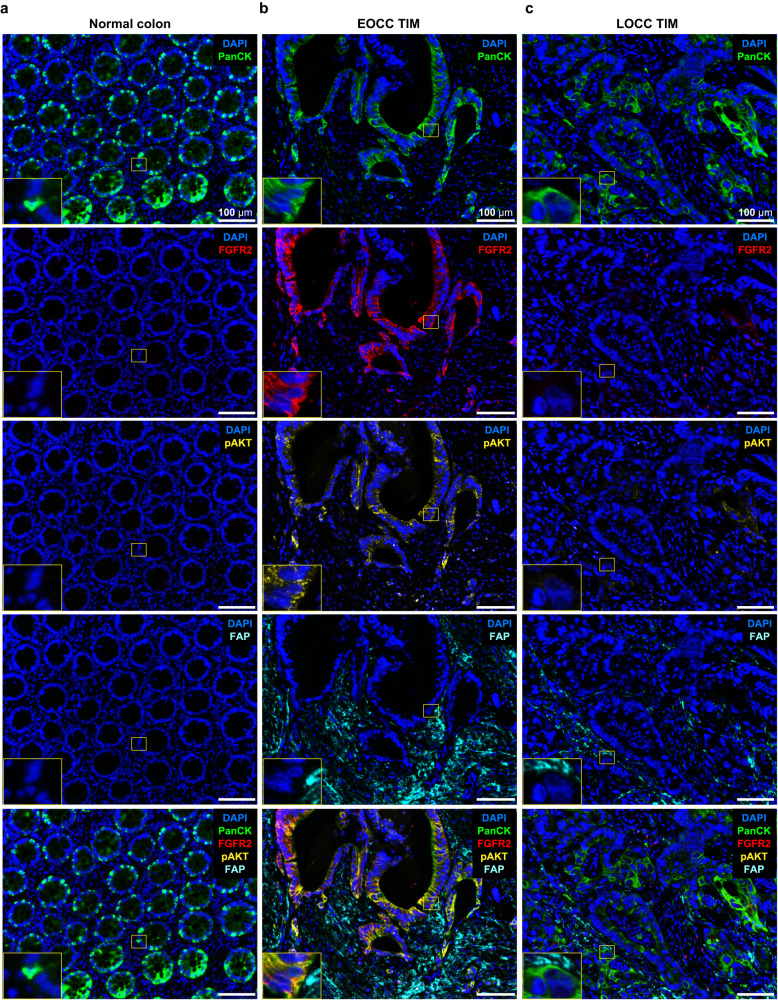
Multiplex immunofluorescences show pAKT staining patterns at tumor epithelial cells of EOCC tumor invasive margin. Representative mIF images of normal colon (**a**), EOCC tumor invasive margin (TIM, **b**), and LOCC TIM (**c**). Tissue samples were stained using the Opal kit. DAPI (blue); PanCK (green, Opal 690); FGFR2 (red, Opal 620); pAKT (yellow, Opal 570); FAP (cyan, Opal 650). The insets represent the magnified image of the yellow box of each picture. EOCC early-onset colon cancer, TIM tumor invasive margin, LOCC late-onset colon cancer, mIF multiplex immunofluorescence.

**Fig. 9:**
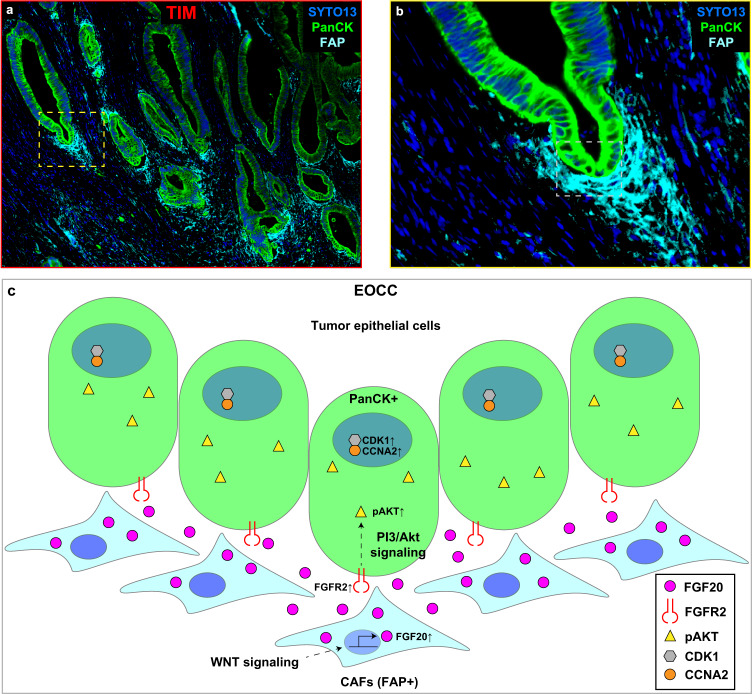
Schematic illustration of the potential interactions of FAP(+) CAFs and PanCK(+) tumor epithelial cells at the EOCC tumor invasive margin. **a** A mIF image showing EOCC tumor invasive margin (TIM). SYTO13 (blue, DNA); pan-cytokeratin (green, PanCK); fibroblast activation protein (cyan, FAP). **b** Magnification of the yellow dotted box in (**a**). **c** Schematic illustration of the white dotted box in (**b**). The illustration summarizes the potential interaction of FAP(+) CAFs and PanCK(+) tumor epithelial cells in EOCC at TIM. FAP(+) CAFs produced high levels of FGF20 because of the WNT signaling overactivation. Neighbor PanCK(+) tumor epithelial cells show FGFR2 and PIK3/Akt upregulation. FGF20 binds to FGFR2 and triggers pAKT activation and CDK1/CCNA2 upregulation.

Furthermore, we also investigated the protein levels of phosphorylated-AKT^S473^ (pAKT) to evaluate the activation of PI3K/Akt signaling in EOCC tumor invasive margin. The staining of pAKT overlapped with FGFR2 detection at the EOCC tumor invasive margin (Fig. 8a–c). Quantitative analysis revealed that pAKT protein levels in PanCK(+) tumor epithelial cells were significantly higher in EOCC tumor invasive margin than in LOCC tumor invasive margin and normal colon tissue areas (Fig. 8a–c, Supplementary Fig. 11g). These results support the hypothesis that FAP(+) CAFs crosstalk with adjacent tumor epithelial cells via the FGF20-FGFR2 interaction that is also associated with activated PI3K/Akt signaling at the EOCC tumor invasive margin (Fig. 9).

## Discussion

In this study, we discovered that FAP(+) CAFs at the EOCC tumor invasive margin showed an upregulation of the WNT signaling and may affect neighbor PanCK(+) tumor epithelial cells via the FGF20-FGFR2-PI3K/AKT signaling pathway.

Genomic analyses showed no significant changes in the frequency of *APC*, *KRAS*, *TP53*, *BRAF*, *NRAS*, and *PIK3CA*^24^ mutations between sporadic EOCC and LOCC, suggesting that the mutations in these genes cannot explain the increased incidence of EOCC^38,39^. Thus, we speculated that the intrinsic profiles of the CAFs are different between sporadic EOCC and LOCC samples. In transcriptomic analysis, we identified DEGs in sporadic tumors derived from EOCC patients and focused on FAP(+) CAFs cell types. To elucidate the cell-specific transcriptomic changes in FAP(+) CAFs of EOCC tumors, we performed CIBERSORTx analysis^26^. The average proportion of FAP(+) CAFs did not differ between EOCC and LOCC patients while depending on the histological location, FAP(+) CAFs showed a significant upregulation of the WNT signaling pathway in EOCC tumors.

Previous studies in colon cancer demonstrated that patients with high-FAP protein levels in stromal areas of the tumors had significantly shorter OS, independent of the patient’s age^40^. However, the limitation is that the analysis was performed using IHC analysis and did not specifically analyze the role of FAP in CAFs. The second limitation is that differences between EOCC and LOCC were not addressed. Using the deconvoluted data, we focused on CAFs in EOCC tumors and stratified CAFs based on the FAP mRNA levels. Supporting previously reported results, we showed that EOCC patients with high-*FAP* mRNA levels in CAFs had poorer clinical outcomes than low-*FAP* levels in CAFs. However, these results did not provide the spatial cell-arrangements and transcriptomic cell-states; both of which are important to understand the role of FAP(+) CAFs in EOCC tumors.

NGDSP analysis and cell deconvolution analysis demonstrated that FAP(+) CAF at EOCC tumor invasive margin showed WNT signaling upregulation. While the tumor-intrinsic roles of the WNT signaling pathway are well established in various tumors including colon cancer^41^, the potential roles of WNT signaling in CAFs remain poorly understood. Supporting our results, Mosa et al. sought to elucidate the distinct CAF populations and categorized CAFs based on WNT activity into myofibroblast CAF with high WNT activity and inflammatory CAF (iCAFs) with low WNT activity^42^. The study showed that the co-culture of tumor organoids with iCAFs resulted in significant upregulation of markers of epithelial-mesenchymal transition (EMT) in tumors, which suggested that iCAFs promote tumor progression^42^.

To gain further insights, we searched for downstream genes controlled by the WNT signaling. By data integration, FGF20 was found upregulated and a potential downstream target of WNT signaling. The FGF family is one of the most diverse growth factor groups in mammals and 22 FGF ligands have been identified in mice and humans^43^. FGF20, as well as FGF9 and FGF16, belongs to the FGF9 subfamily. All FGF9 members activate, by paracrine binding, high-affinity tyrosine kinase receptors that are coded by four genes (FGFR1, FGFR2, FGFR3, and FGFR4)^36^. The FGF/FGFR downstream signaling pathways include PI3K/Akt, MAPK, and JAK/STAT3, all of which regulate cell proliferation, differentiation, and survival^44,45^. Our results found that FGF20 is a downstream target of the WNT signaling in FAP(+) CAFs at EOCC tumor invasive margin and suggested that FGF20 may represent a ligand for FGF receptors.

Past studies indicated that genetic alterations in FGFRs are associated with tumor progression in solid tumors^46^, and although the genetic alterations in CRC are limited, some studies have shown responses to FGFR inhibitors in CRC patients that have FGFR alterations^47^. Nevertheless, the transcriptomic alterations and the spatial transcriptomic changes of FGF-FGFR signaling and their association with CRC are less explored. In this study, we found that FGF20 was specifically detected in FAP(+) CAFs areas that were closely located to PanCK(+) tumor epithelial cells that had positive FGFR2 detection at EOCC tumor invasive margin. Of all the FGFR screens in the analysis, only FGFR2 was identified as the FGF20 receptor by an integrated ligand-receptor network using the NicheNet program. Thus, we proposed that in neighbor PanCK(+) tumor epithelial cells, FGFR2 is a potential receptor that binds to FGF20. Future functional characterization is warranted to demonstrate the intercellular crosstalk between FAP(+) CAFs producing FGF20 and tumor epithelial cells having enhanced FGFR2 protein levels at the tumor invasive margin of EOCC. Our results support a potential interplay between the CAFs and tumor epithelial cells, which promotes the activation of FGFR2-PI3K/Akt signaling in tumor cells at the tumor invasive margin of EOCC.

In conclusion, our study demonstrate how spatial cell-states like FAP(+) CAFs may have clinical implications at the tumor invasive margin of EOCC tumors by affecting neighbor tumor epithelial cells. Future studies are needed to validate our proposed mechanisms and the implications of CAFs at the tumor invasive margin of EOCC tumors.

## Methods

### Ethics approval

The study was conducted following the Declaration of Helsinki. Human samples and clinical information for this study were obtained according to the protocol guidelines approved by Providence SJHC under SJHC/SJCI Joint Institutional Review Board (IRB): Universal Consent (Providence Health and Services Portland IRB: JWCI-18-0401) and Western IRB: MORD-RTPCR-0995. All archival FFPE tissue specimens and patients in the study were de-identified and HIPAA regulations were followed. Written informed consent was obtained from the participants to participate in the study.

### Patient selection

The Providence Saint John’s Health Center cohort consisted of colon cancer patients diagnosed with pathological colon cancer. All 26 patients underwent surgery for colon cancer in SJHC between 2015 and 2020. The following data were collected from each patient chart: patient baseline characteristics at the time of operation including, age at diagnosis, gender, race, family history, comorbidities of inflammatory bowel diseases, tumor location, the presence of preoperative treatment, and pathological features of the resected specimens. Patients were divided into young (EOCC, <50 yr.) and old (LOCC, ≥50 yr.) patient groups, according to the definition of EOCC described in previous studies^9,48^. Patients with any suspicious family history of hereditary colon cancer, a known genetic predisposition for colon cancer, or the comorbidity of inflammatory bowel disease were excluded. Patients who had received any preoperative therapy were also excluded. The location of tumors was defined as right-sided (cecum, ascending colon, hepatic flexure, transverse colon, and splenic flexure) or left-sided (descending colon, sigmoid colon), according to the International Classification of Diseases (ICD)−10 classification. The pathological features were determined following the Tumor, Node, and Metastasis (TNM) system, based on the eighth edition of the American Joint Committee on Cancer. DNA MMR status was evaluated by immunohistochemical staining of MLH1, PMS2, MSH2, and MSH6 by the Pathology Dept. at SJHC, as previously described^49^. Patients with loss of two or more of these four genes were classified as microsatellite instability-high (MSI-H) and excluded from the study. The quality of all FFPE sections was evaluated using hematoxylin and eosin (H&E) staining. All FFPE sections included in the study were evaluated by a board-certified pathologist at the Pathology Dept. at SJHC. Detailed clinicopathological information about the patients is listed in Supplementary Tables 2–4. All the 26 patients included were analyzed by HTG-EdgeSeq PIP (Supplementary Tables 2, 3). A cohort of 8 patients, out of the 26 patients included in the study, were assessed by NGDSP. Four patients were diagnosed with EOCC, and four patients were diagnosed with LOCC (Supplementary Table 2). A cohort of 8 patients were assessed by Opal staining (Supplementary Table 2). The patients analyzed by the three different assays overlapped (Supplementary Table 2).

### NanoString GeoMx DSP analysis

Tissue preparation was performed according to the vendor’s protocol with slight modifications. 5 µm thick FFPE tissue sections were baked at 60 °C for 30 min, then the slides were sequentially incubated three times for 5 min in xylene, and then twice for 5 min in each 100% ethanol, 95% ethanol, then water. Antigen was retrieved by placing slides in a staining jar containing tris ethylenediamine tetra acetic acid (EDTA, pH 9) and incubated at low pressure at 100 °C for 20 min, followed by a 5 min wash in phosphate buffer-saline (PBS) pH 7. Thereafter, slides were placed in a staining jar with 1 mg/mL proteinase K and then incubated at 37 °C for 15 min. After proteinase digestion, slides were washed in 10% neutral buffered formalin (NBF) for 10 min. This step was followed by two washes in NBF stop buffer (100 mM Tris-HCl and 100 mM glycine) and one wash in PBS. The RNA probe mix (the CTA probe set covers 1811 unique genes that were summarized in https://nanostring.com/products/geomx-digital-spatial-profiler/geomx-rna-assays/geomx-cancer-transcriptome-atlas/) was diluted with buffer R, added to the tissue slides, and covered with a hybridization coverslip (GybriSlip, Grace Bio-Labs, Bend, OR), and incubated overnight at 37 °C. Slides were washed twice with a stringent wash buffer containing Saline-Sodium Citrate (SSC) buffer and formamide at 37 °C and then twice with SSC buffer. The slides were then stained for 1 h with fluorescently labeled morphology markers as follows: SYTO13 (nucleus), pan-cytokeratin (PanCK, epithelial cells), vimentin (VIM, normal fibroblasts), and FAP (CAFs). VIM was selected as a marker of normal fibroblast since VIM is known to be highly expressed in fibroblasts of all types and used as a common method to visualize fibroblast populations^50,51^. After being washed twice in the SSC buffer, slides were loaded on the NGDSP instrument for imaging and barcode acquisition, following the manufacturer’s protocol. Slides were scanned based on morphological markers at 20× magnification using a GeoMx DSP instrument. The regions of interest (ROI)s were manually selected using circular or freeform regions measuring around 600–650 µm in diameter. ROIs were segmented into a PanCK(+), VIM(+), or FAP(+) to obtain specific AOIs. With a double digital mirror device module, ultraviolet (UV) light illuminated the segmented AOIs to release the ligated index oligos. The released oligos were collected by the microcapillary arm and then aspirated into an individual well of a microtiter plate. After collection, the microtiter plate was sealed with a semi-permeable film and dried overnight at room temperature.

Libraries were generated according to NanoString’s Next Generation Sequencing (NGS) readout protocol as follows. Dried aspirates in the collection plate were resuspended with 10 µL of nuclease-free water for 10 min, and 4 µL were transferred to a polymerase chain reaction (PCR) plate. Two µL of 5X PCR Master Mix (NanoString Technologies, Inc., Seattle, WA) and 4 µL of a unique primer mix (NanoString) assigned by their position on the collection plate were added to each well. Contents were mixed by pipetting and the sealed PCR plate was transferred to a thermal cycler for an amplification for 18 cycles. PCR products were pooled in equal amounts. Pooled library was purified twice with a 1.2X magnetic beads-to-sample ratio with KAPA Pure Beads (Roche, Basel, Switzerland), each followed by two 80% ethanol washes, then eluted with Elution Buffer (NanoString). The quality and quantity of the purified pooled library were assessed with High Sensitivity D1000 ScreenTape System (Agilent, Santa Clara, CA) and Qubit dsDNA High Sensitivity assay (Invitrogen, Waltham, MA), respectively. The library was denatured following Illumina’s recommendations and sequenced on NextSeq 550 (Illumina, San Diego, CA) with 27 paired-end cycles, achieving the minimum read depth (30 reads per µm^2^) or higher determined by the selected AOI’s sizes.

### Data processing using GeoMx DSP software

The FastQ files generated by the Illumina sequencing were converted into digital count conversion (dcc) files, then loaded into the GeoMx DSP analysis suite as raw counts. The GeoMx DSP software captures the number of SYTO13 positive nuclei in each segment as well as the surface area of each segment, which were exported for data calibrations. From the software, the counts for oligonucleotides per segment were also exported into a package for quality control and data normalization protocols. Biological probe quality control was performed using default settings. A total of 112 AOIs were all carefully reviewed for segments with sequencing saturation of less than 45%, nuclei of fewer than 160, negative probe counts of less than 8, or a surface area of less than 12,000 squared microns. All 112 AOIs were included for further analysis following the NanoString user’s manual recommendations. Background correction and scaling were performed using geometric means and normalization was performed using Q3 averages of housekeeping genes. Genes with expression levels at or lower than the limit of quantification (LOQ) in at least 5% of segments were filtered out. A total of 1223 genes passed quality control and were analyzed.

Eight patients with four sporadic EOCC and four sporadic LOCC were processed. Per patient, a total of six ROIs with tumor invasive margin (three ROIs) and tumor center (tumor center, three ROIs), were selected, respectively. The definitions of tumor invasive margin and tumor center were considered based on the histological border from H&E staining using consecutive sections, according to past published reports^52–54^. Tumor invasive margin was defined as the areas on the border of malignant cells close to normal tissue based on pathological features (Supplementary Figs. 5–8). Tumor center was defined as central tumor tissue at least 1 mm away from tumor invasive margin and facing the lumen side (Supplementary Figs. 5–8). In addition, 8 ROIs were selected at adjacent normal (AN) mucosal tissue with two EOCC and two LOCC, respectively (Supplementary Figs. 5–8). Adjacent normal was defined as non-tumoral mucosal areas adjacent to the tumor, without any irregularity of nucleus in the epithelium. In each ROI, an AOI was selected according to the morphological markers (epithelial cells at adjacent normal, tumor center, and tumor invasive margin: PanCK(+), VIM(-), FAP(-); normal fibroblasts at adjacent normal and tumor center: PanCK(-), VIM(+), FAP(-); CAFs FAP(+) at tumor invasive margin: PanCK(-), VIM(+), FAP(+). FAP(+) AOIs were only considered at tumor invasive margin, since the estimated number of cells with FAP(+) staining at tumor center and adjacent normal did not reach the minimum number of cells required to be sequenced. Finally, 96 AOIs from colon cancer tumors and 16 AOIs from adjacent normal areas were analyzed (Supplementary Fig. 5). The average AOI’s area was 57,921 μm^2^ and comprised 691 cells on average per AOI.

### Sample Processing for HTG EdgeSeq PIP Assay

Thirteen EOCC tumors, 13 LOCC tumors, and the respective 26 paired-adjacent normal colon tissues were processed (Supplementary Tables 2–3). Normal tissues were defined as normal epithelial areas at least 2 cm away from the tumor margin. Using 5 µm unstained FFPE sections, the tumor areas were marked by referring to the H&E staining of the consecutive slide, with the accordance of a certificated pathologist. The estimated tissue areas were measured using Image J software. Then, the optimal amounts of tissue for each HTG EdgeSeq PIP assay were scraped and placed into microfuge tubes. The calculated volume of lysis buffer for each collected sample was added, and the samples were then overlaid with 500 µl of HTG denaturation oil. After centrifugation, the samples were incubated at 95 °C for 15–20 min to denature protein structures and remove paraffin wax from the FFPE tissue sections. Samples were cooled down for 10 min at room temperature (RT), and HTG-provided Proteinase K was pipetted into the aqueous (lysis, non-oil) phase of the samples at a volume 1/20th of the total lysis buffer. Finally, the samples were incubated at 50 °C for 3 h, with the aqueous phase being mixed by pipetting every 30 min. Subsequently, 25 µL of the sample lysate was loaded onto the HTG EdgeSeq instrument for probe-capture for 20 h.

### Library preparation for HTG EdgeSeq PIP asay

Probe-captured FFPE samples for EdgeSeq PIP assay were amplified and indexed via polymerase chain reaction (PCR) using the master mix (OneTaq HotStart 2X MasterMix in GC Buffer (New England Biolabs Inc., Ipswich, MA, USA), and indexing primers. The PCR reaction conditions were as follows: (1) 95 °C for 4 min, (2) 95 °C for 15 s, (3) 56 °C for 45 s, (4) 68 °C for 45 s, (5) repeating steps 2–4 for a total of 19 cycles, (6) 68 °C for 10 min, and (7) holding at 4 °C. Following PCR, library cleanup was performed with a mixture of clean up buffer (39% of 5 M NaCl, 31.25% of 40% PEG 8000, 29.75% of molecular-grade water) and AMPure XP beads, by combining it with the PCR-amplified sample at 5:2 ratio, respectively. After 5 min of incubation, the samples were placed on the magnetic stand, then washed twice with 80% ethanol, followed by a 5-min air drying period. The elution was performed using 40 µL of 10 mM Tris-HCl, pH 8.0.

### NGS library quality check for HTG EdgeSeq PIP assay

All libraries were quantitated using the KAPA Library Quant Kit (Illumina Inc., San Diego, CA, USA) and the Universal qPCR Mix Kit (Roche, Basel, Switzerland) in accordance with the manufacturer’s recommendations. Quality checks (QC) for library size were performed on the Agilent Technologies TapeStation 2200 instrument using the High Sensitivity D1000 ScreenTape and High Sensitivity D1000 reagents (Agilent Technologies Inc., Santa Clara, CA, USA). The expected peak size ranged between 150 and 170 base pairs. Samples that did not indicate proper library formation were excluded from sequencing and the library preparation process was repeated.

### NGS library normalization and pooling for HTG EdgeSeq PIP assay

Quantitated libraries were diluted, normalized, and pooled based on the raw quantity (pM) generated by the HTG EdgeSeq RUO Library calculator software version 2.0.0. Then, libraries were denatured in 0.2 N NaOH for 8 min at RT. NaOH was hydrolyzed with 200 mM Tris pH 7.4, and the denatured pool was then diluted down to 4 pM using the Hyb buffer supplied in the NextSeq 550 High Output Kit v2.5 (Illumina Inc., San Diego, CA, USA). To introduce sequencing diversity and a positive sequencing control, 4 pM of 12.5 pM *Phi*X Control v3 (Illumina Inc., San Diego, CA, USA) was spiked into the diluted and denatured 4 pM sample pool. The final pool consisted of 95% sample libraries and 5% *Phi*X control by volume. The pooled library was then denatured at 98 °C for 4 min and immediately placed on ice for at least 5 min before loaded onto the Illumina NextSeq 550 instrument, following the Illumina instrument sequencing protocol.

### NGS profiling of the Libraries for HTG EdgeSeq PIP assay

Sequencing on Illumina platforms was conducted according to the HTG instructions, with a read length of 1 × 50 base pairs. The raw sequencing data was transformed into FASTQ files using Illumina BaseSpace BCL to FASTQ software version 2.2.0 and Illumina Local Run Manager Software version 2.0.0. FASTQ files were analyzed with HTG EdgeSeq Parser software version v5.1.724.4793 to generate raw counts for a total of 1410 genes. Each sample was profiled for 1392 genes related to tumor-immune interaction, in addition to 18 control genes.

### Targeted RNA-Seq data analysis

The software platform HTG EdgeSeq Reveal (HTG REVEAL software version 2.0.1, http://reveal.htgmolecular.com) was used for data analysis. The raw reads count was normalized using: https://bioconductor.org/packages/release/bioc/vignettes/DESeq2/inst/doc/DESeq2.html. Differential Expression Outputs including mean normalized values in each group, fold change (FC), raw *p* value and adjusted *p* value (*p* value for each probe after adjustment using the Benjamini and Hochberg method for controlling the false discovery rate) between groups were calculated. PCA was used to determine sample clustering. In all PIP data comparisons, a Log2|FC | ≥ 1 and adjusted *p* value < 0.05 as significant difference between groups was considered.

### Evaluation of immunofluorescence intensity and proportion using Qupath software

The FAP immunofluorescence (IF) intensity and proportion in scanned images in the GeoMx DSP instrument were quantitatively evaluated. Briefly, a 20× magnifying image in each area (adjacent normal, tumor center, and tumor invasive margin) was taken by GeoMx DSP instrument and uploaded in Qupath software (v.0.3.2, Queen’s University, Belfast, Northern Ireland). The stromal area was manually segmented as PanCK(-) region according to negative selection of PanCK(+) protein expression, and H-scores of cytoplasmic FAP(+) staining cells in each interested area were automatically calculated using Qupath built-in “Positive cell detection”^55^. The optical signal threshold to classify the score into 4-bins was set to 10, 30, and 100. Eight FFPE tissue slides from eight patients (4 EOCC and 4 LOCC) were analyzed and, in each slide, three 20× pictures per each area (adjacent normal, tumor center, and tumor invasive margin) were captured. The mean scores in each area were compared for statistical significance.

### Multiplex immunofluorescence using Opal Kit

Multiplex immunofluorescence was performed using Opal 7-Color Manual IHC Kit (NEL 811001KT, Akoya Biosciences), which relies on individual tyramide signal amplification-conjugated fluorophores to detect various targets. Staining was performed following the manufacturers’ instructions with slight modifications. After deparaffinization, slides were placed in a plastic container filled with AR buffer (blocking/Ab diluent, Akoya Biosciences). AR buffer was microwaved for 165 s at high power to reach 100 °C. Then, slides were microwaved in AR buffer at low power (75 °C) for an additional 15 min. Slides were then cooled down for 15 min at room temperature and rinsed with deionized water and TBS-T. Peroxidase blocking was performed using 3% H_2_O_2_ for 10 min and slides were incubated additionally for 10 min with Ab Diluent/Blocking solution (Akoya Biosciences) to initiate protein stabilization and background reduction. Slides were then incubated with primary Abs (Panel 1: PanCK, CD45, and FAP; Panel 2: PanCK, FAP, and FGF20; Panel 3: PanCK, FAP, and FGFR2; Panel 4: PanCK, FAP, FGFR2, and pAKT; Panel 5: PanCK, FAP, FGFR2, and FGF20). The slides were washed and incubated for 10 min at room temperature with the secondary Ab (Opal polymer HRP Mouse/Rabbit, Akoya Biosciences). After two washes in TBS-T, the slides were then incubated at room temperature for 10 min with one of the following Alexa Fluor tyramides (Akoya Biosciences) included in the Opal kit to detect Ab staining and prepared according to the manufacturer’s instructions: Opal 540, Opal 570, Opal 620, Opal 650, and Opal 690. After incubation, the slides were washed twice with TBS-T. Then, the slides were microwaved to detach the primary and secondary Abs. After all reactions (3 to 4 rounds of microwave treatment, primary Ab, secondary Ab, and Alexa Fluor tyramide incubation), slides were counterstained with 4′, 6′- diaminodino-2-phenylindole (DAPI) and mounted with Mowiol 4–88 mounting media (prepared as described by manufacturer’s instructions). Patients’ information is shown in Supplementary Tables 2–5. The information about primary Abs and the corresponding fluorophores are listed in Supplementary Table 13.

Multiplex-stained slides were imaged using the Mantra Multispectral Imaging System (v1.0, Akoya Biosciences). All samples were captured at 20× and/or 40× magnification. Filter cubes used for multispectral imaging were DAPI (440–680 nm), fluorescein isothiocyanate (FITC, 520–680 nm), and Cy3 (570–690 nm), Texas Red (580–700 nm), and Cy5 (670–720 nm). The signal intensities for each marker were normalized, and spectral unmixing was performed with InForm Analysis software (v.2.6.0, Akoya Biosciences). Images encompassing the entire slide through the full emission spectrum of each filter (DAPI, FITC, Cy3, Texas Red, and Cy5) were captured. A spectral signature for each fluorophore was obtained by using the same multispectral imaging protocol of a single-stained slide, as well as an unstained slide to obtain the auto-fluorescence signature of the tissue. Images of the single-stained tissues and unstained tissues were used to extract the spectrum of each fluorophore and tissue autofluorescence, respectively, and to establish a spectral library required for multispectral unmixing.

For the scoring of the protein levels in tissue samples, H-scores and double positive scores were calculated using the InForm software (Akoya Biosciences) according to manufacturers’ instructions and as previously described^56^. For H-scores, the PanCK(+) area or PanCK(-) stromal area were semi-automatically segmented according to positive or negative PanCK staining, and nuclei/cytoplasm compartments were distinguished by detecting the intensity of nuclear staining. The optical signal threshold to classify the score into 4-bins was set to 2, 8, and 30 for FAP, 0.2, 0.4, and 0.6 for FGF20, 0.15, 0.3, and 0.45 for FGFR2, 0.15, 0.3, and 0.45 for pAKT, respectively. The optical signal threshold to classify the double positive scores was set to 8 for FAP, 0.4 for FGF20, 0.6 for PanCK, and 0.3 for FGFR2, respectively. For validation of FAP expression, six FFPE tissue slides from sporadic EOCC (*n* = 3), and LOCC (*n* = 3) were analyzed. For validation of FGF20, FGFR2, and pAKT protein expression, FFPE tissue slides from sporadic EOCC (*n* = 5), LOCC (*n* = 5), and normal colon (*n* = 2) were analyzed. Normal colon samples were defined as those separated at least two centimeters away from the tumor margin. Three to four photographs per sample were captured on each slide at 20X magnification. The median H-scores and double positive scores in each area were compared for statistical significance.

### TCGA COAD database

The TCGA COAD dataset (RNA-Seq) was downloaded from UCSC Xena (https://xena.ucsc.edu/). Among 512 tissue samples, 472 samples were successfully annotated with clinical information. Patients with MSI-H or the presence of any preoperative treatment were excluded, resulting in 454 samples for analysis. Samples were categorized into early-onset colon cancer (EOCC, <50 yr., *n* = 53) and late-onset colon cancer (LOCC, ≥50 yr., *n* = 401), respectively. A total of 13,145 genes were included in the analysis; genes with max counts <3 were excluded. Normal tissues from COAD patients who had gene expression profiles (*n* = 40) were used in different comparisons. The TCGA COAD whole-exome sequencing dataset, to determine gene mutation status, was downloaded from cBioPortal (https://www.cbioportal.org/). The consensus molecular subtyping (CMS) status of the TCGA COAD dataset was downloaded from synapse (http://www.synapse.org).

### Immunohistochemistry

Immunohistochemical staining was performed as previously described^56^. After deparaffinization and rehydration, sections were incubated with 10 mM citrate buffer (pH 6.0), at 100 °C for 20 min for antigen retrieval (AR). Then sections were cooled to room temperature for 20 min. Endogenous peroxidase was quenched with 3% hydrogen peroxide (H_2_O_2_) for 10 min at room temperature. After permeabilization with 0.4% Triton X buffer for 20 min, sections were exposed to a blocking solution (Protein Block Serum-Free; Dako, Carpinteria, CA). The sections were then incubated overnight with the primary antibody (Ab). The next day, the sections were washed with Tris-buffered saline-tween 20 (TBS-T), and sections were incubated with the secondary biotinylated Ab (K0675, Dako) and system horseradish peroxidase (HRP) for 30 min, followed by three 5-min rounds of Phosphate-buffered saline (PBS) washings. Staining signals were developed using 3,3′-diaminobenzidine (Dako). Sections were counterstained via Gill’s hematoxylin (Fisher Scientific, Waltham, MA) and then mounted. The representative pictures were captured using the Mantra Multispectral Imaging system (Akoya Biosciences, Marlborough, MA). The Abs and dilutions utilized are listed in Supplementary Table 5.

### CIBERSORTx

CIBERSORTx (https://cibersortx.stanford.edu/) was used to estimate cellular abundances and gene expression of each cell phenotype. GSE39396 (Supplementary Table 1) from Gene Expression Omnibus (GEO) database (https://www.ncbi.nlm.nih.gov/geo/) was applied as reference signature gene matrices following the manufacturer’s online protocol. In the GSE39396 dataset, six colorectal fresh tumors were analyzed. Briefly, EPCAM(+), FAP(+), CD45(+), and CD31(+) cell populations were purified by fluorescence-activated cell sorting. EPCAM(+), FAP(+), CD45(+), and CD31(+) cell populations were profiled by microarray to obtain the mRNA profiles. A second colon cancer dataset GSE39582 was downloaded from the GEO database (Supplementary Table 1). GSE39582 had transcriptome data from colon cancer patients with clinical information for age and microsatellite instability (MSI) status available. Colon cancer patients with deficient mismatch repair (MMR) status (*n* = 77) were considered MSI-H and those with MSI-H were excluded from the analysis. Total of 508 MSS patients with EOCC (<50 yr., *n* = 54) and LOCC (≥50 yr., *n* = 454) were imputed to cell fractions mode to determine the cellular abundances of each phenotype in each sample imputed, using the reference signature gene matrix. The mRNA data were deconvoluted using high-resolution cell expression mode to show the estimated gene expression profile in each phenotype.

For deconvolution of the TCGA COAD dataset (EOCC, *n* = 53, LOCC *n* = 401), the scRNA-seq dataset GSE146771 was downloaded from the GEO database (Supplementary Table 1) and applied as reference signature matrices to match the sequencing platform. Total 454 MSS patients from TCGA COAD dataset were imputed for cell fraction mode and high-resolution cell expression mode to estimate the cellular abundances and gene expression profile in each cell type. Generated gene expression data in cancer-associated fibroblast (CAF)s were converted to Log2 value and the *FAP* mRNA levels were compared between EOCC and LOCC patients. Patients were stratified based on high-FAP and low-*FAP* mRNA levels, which were defined by median values. Overall survival, disease-specific survival, and progression-free interval rates were determined by using Kaplan-Meier methods and significant differences were assessed using the Log-rank test.

### Gene set enrichment analysis

Gene set enrich analysis (GSEA) application (Broad Institute of Massachusetts Institute of Technology, https://www.gsea-msigdb.org/gsea/index.jsp), was used to compare gene expression profiles between EOCC and LOCC. The gene set database c2.cp.wikipathways.v7.5.1. symbols.gmt was used for the analysis. Normalized enrichment score (NES) was calculated and used to compare the results across gene sets. NES with a false discovery rate (FDR) of <0.25 was defined as significant.

### Ligand and receptor-based cell interaction prediction analysis

The NicheNet algorithm is a method that predicts which ligands produced by one cell regulate the expression of which target genes in another cell^35^. Ligand–receptor links are inferred by combining bulk or scRNA-seq data of interacting cells with existing knowledge of signaling and gene regulatory networks. In this study, the NicheNet algorithm was used to determine potential paracrine communications between FAP(+) CAFs and neighbor tumor epithelial cells (PanCK+). To investigate how FAP(+) CAFs influence neighbor tumor epithelial cells (PanCK+) at tumor invasive margin, FAP(+) CAFs cells and PanCK(+) tumor epithelial cells were considered as “sender cells” and “receiver cells”, respectively. For ligand and receptor interactions, 323 upregulated DEGs in CAFs (FAP+) and 187 upregulated DEGs in PanCK(+) tumor epithelial cells at EOCC tumor invasive margin were imputed as “expressed gene senders” and “expressed genes receivers”, respectively. Potential ligands in FAP(+) CAFs and potential receptors in PanCK(+) tumor epithelial cells were defined using the computational ligand-receptor network. 1223 genes that were listed in the CTA and passed LOQ were used for background genes. The DEGs related to the PI3K/Akt signaling pathway in PanCK(+) tumor epithelial cells at EOCC tumor invasive margin were imputed as specific genes of interest. The indicated score of interaction potential accords with the weight of the interaction between the ligand and receptor in the integrated weighted ligand signaling network of NicheNet. An open-source R package “nichenetr” is available on GitHub (https://github.com/saeyslab/nichenetr).

### Western blot analysis

HT29 cell lines (HTB-38, ATCC, VA, USA) were treated with recombinant FGF20 (cat#: 2547-FG-025, R&D Systems) for 30 and 60 min or left untreated. Protein extraction was performed as previously described^57–59^. Traditional western blot was performed as previously described^57–59^, except for the antibodies utilized are summarized in Supplementary Table 6. All western blot images were analyzed with ImageJ software (http://imagej.nih.gov/ij/). All the blots shown in Fig. 7f and Supplementary Fig. 15 were derived from the same experiment and were processed in parallel. All the uncropped western blot images were included in Supplementary Fig. 15.

### Statistical Analysis

All the statistical analyses were performed using GraphPad Prism 7 software (GraphPad software Inc., La Jolla), GeoMx DSP software, or R 4.2.1 version in a two-tailed way. The distribution and variation within each group of data were assessed before selecting the correct statistical analysis. Fisher’s exact test or Chi-square tests were used to compare nominal variables. Student’s *t* test, linear mixed model, or Mann-Whitney U test was used for comparison between the two groups. Benjamini-Hochberg correction was used to decrease the FDR. Multiple groups were compared by one- or two-way Analysis of Variance (ANOVA) followed by post-hoc tests. The correlation was determined by Spearman’s correlation test. The Kaplan-Meier method and Wilcoxon test were used to estimate prognosis. All the figures were unified using Adobe Illustrator Creative Cloud (Adobe Inc., Los Angeles, CA). All data were presented as mean ± standard error mean (SEM) or median (range). * *p* < 0.05, ** *p* < 0.01, and *** *p* < 0.001 was indicated as statistically significant.

### Reporting summary

Further information on research design is available in the Nature Research Reporting Summary linked to this article.

### Supplementary information

Supplementary Information

REPORTING SUMMARY

## Data Availability

The data that support the findings of this study has been deposited in GEO database under GSE240624 SuperSeries which includes GSE240531 for Nanostring GeoMx DSP dataset and GSE240623 for HTG-EdgeSeq PIP dataset. The rest of the publicly available data utilized and obtained from GEO or TCGA databases have been described in Supplementary Table 1.

## Code availability

Codes are available upon request.
