# Supplementary material for: Spatial profiling of cancer-associated fibroblasts of sporadic early onset colon cancer microenvironment
Source: NPJ Precis Oncol. 2023 Nov 14;7:118. doi: 10.1038/s41698-023-00474-w (PMC10645739; doi:10.1038/s41698-023-00474-w)
Supplement: Supplementary file 1 — Supplementary Information [file 41698_2023_474_MOESM1_ESM.pdf]

Spatial profiling of cancer associated fibroblasts of sporadic early onset colon cancer microenvironment

Satoru Furuhashi[1], Matias A. Bustos[1], Shodai Mizuno[1], Ryu Suyeon[2], Yalda Naeini[3], Anton J. Bilchik[4], and Dave S. B. Hoon[1, 2]

Affiliations of authors:

[1]Department of Translational Molecular Medicine, Saint John's Cancer Institute (SJCI), Providence Saint John's Health Center (SJHC), Santa Monica, CA 90404, USA.

[2]Department of Genome Sequencing Center, SJCI, Providence SJHC, Santa Monica, CA 90404, USA.

[3]Department of Surgical Pathology, Providence SJHC, Santa Monica, CA 90404, USA. [4]Department of Gastrointestinal and Hepatobiliary Surgery, Providence SJHC, Santa Monica, CA 90404, USA.

Supplementary Figures

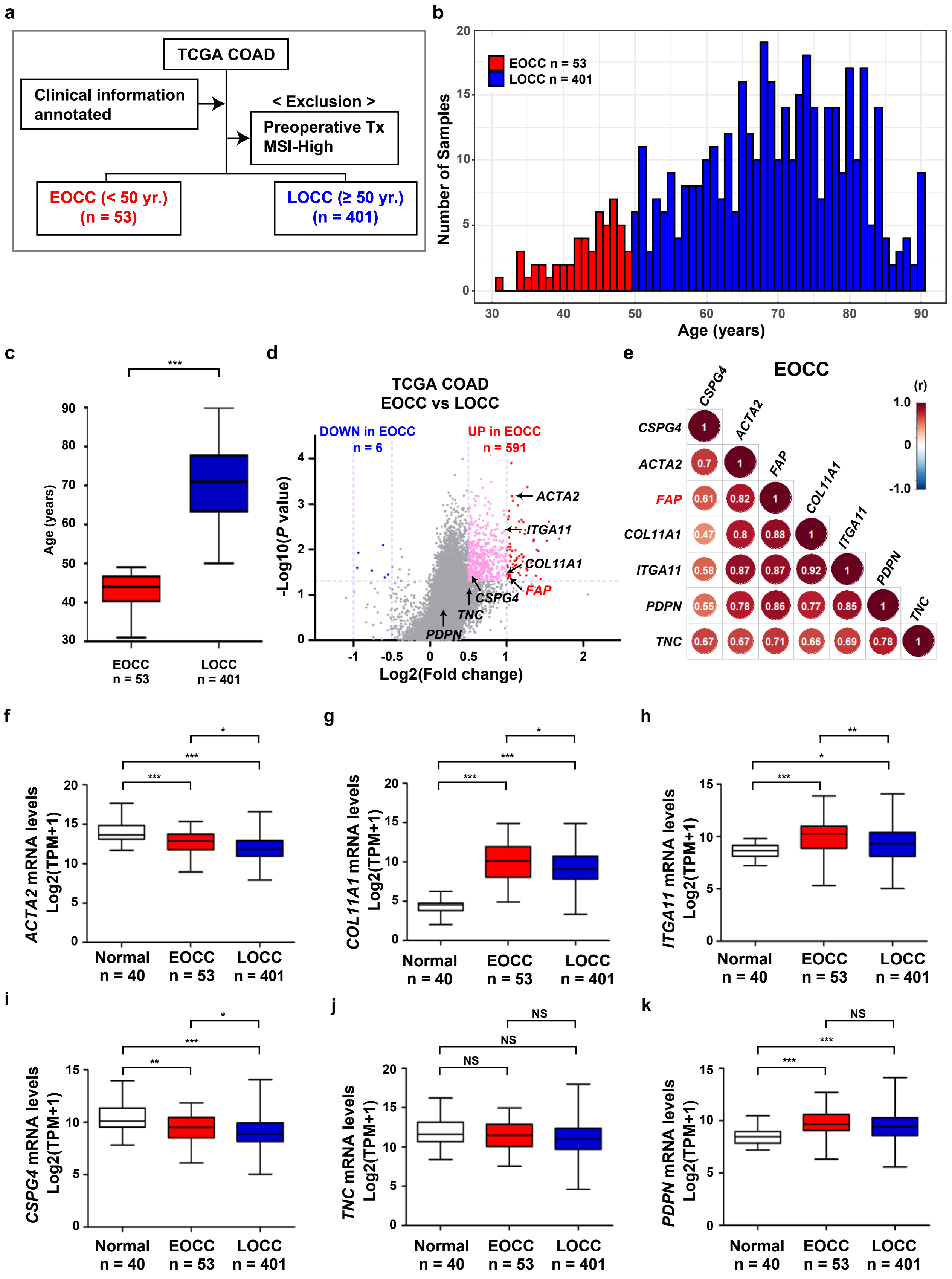

a
TCGA COAD
Clinical information annotated
< Exclusion >
Preoperative Tx
MSI-High
EOCC (< 50 yr.) (n = 53)
LOCC (≥ 50 yr.) (n = 401)
b
EOCC n = 53
LOCC n = 401
Number of Samples
Age (years)
c
Age (years)
EOCC n = 53
LOCC n = 401
d
TCGA COAD
EOCC vs LOCC
DOWN in EOCC n = 6
UP in EOCC n = 591
ACTA2
ITGA11
COL11A1
FAP
CSPG4
TNC
PDPN
-Log10(P value)
Log2(Fold change)
e
EOCC
(r)
CSPG4
ACTA2
FAP
COL11A1
ITGA11
PDPN
TNC
f
ACTA2 mRNA levels Log2(TPM+1)
Normal n = 40
EOCC n = 53
LOCC n = 401
g
COL11A1 mRNA levels Log2(TPM+1)
h
ITGA11 mRNA levels Log2(TPM+1)
i
CSPG4 mRNA levels Log2(TPM+1)
j
TNC mRNA levels Log2(TPM+1)
NS
k
PDPN mRNA levels Log2(TPM+1)

Supplementary Figure 1: CAF-related gene markers are upregulated in sporadic MSS EOCC a Flow chart showing the criteria for case selection in the TCGA COAD dataset and data categorization into EOCC (< 50 yr., n = 53) and LOCC (≥ 50 yr., n = 401). **b** A histogram showing the age distribution of MSS COAD dataset. **c** Boxplot showing age differences in EOCC and LOCC patients. **d** Volcano plot showing the 597 differentially expressed genes between EOCC and LOCC; 591 were upregulated (red dots: $Log_2(FC) \geq 1$; pink dots: $Log_2(FC) \geq 0.5$ and ≤ 1) and six were downregulated in EOCC (blue dots, $Log_2(FC) \leq 0.5$). **e** Hierarchical correlation plot showing the spearman correlation values among the mRNA levels of the seven CAF-related genes in the EOCC dataset. **f-k** Box plot charts showing *ACTA2* (**f**), *COL11A1* (**g**), *ITGA11* (**h**), *CSPG4* (**i**), *TNC* (**j**), and *PDPN* (**k**) mRNA levels ($Log_2(TPM+1)$) in normal tissues (white), EOCC tissue (red) and LOCC tissue (blue) using the TCGA COAD dataset. *p <0.05, **p <0.01, ***p <0.001. NS, not significant; FC, fold-change; TPM, transcripts per million; TCGA, The Cancer Genome Atlas; COAD, colon adenocarcinoma; Tx, treatment; MSI, microsatellite instability; EOCC, early-onset colon cancer; LOCC, late-onset colon cancer; (r), correlation coefficient.

Spatial profiling of a unique cancer-associated fibroblast population in the tumor microenvironment in sporadic early-onset colon cancer

Satoru Furuhashi[1], Matias A. Bustos[1], Shodai Mizuno[1], Ryu Suyeon[2], Yalda Naeini[3], Anton J. Bilchik[4], and Dave S. B. Hoon[1, 2]

Affiliations of authors:

[1]Department of Translational Molecular Medicine, Saint John's Cancer Institute (SJCI), Providence Saint John's Health Center (SJHC), Santa Monica, CA 90404, USA.

[2]Department of Genome Sequencing Center, SJCI, Providence SJHC, Santa Monica, CA 90404, USA.

[3]Department of Surgical Pathology, Providence SJHC, Santa Monica, CA 90404, USA. [4]Department of Gastrointestinal and Hepatobiliary Surgery, Providence SJHC, Santa Monica, CA 90404, USA.

Supplementary information

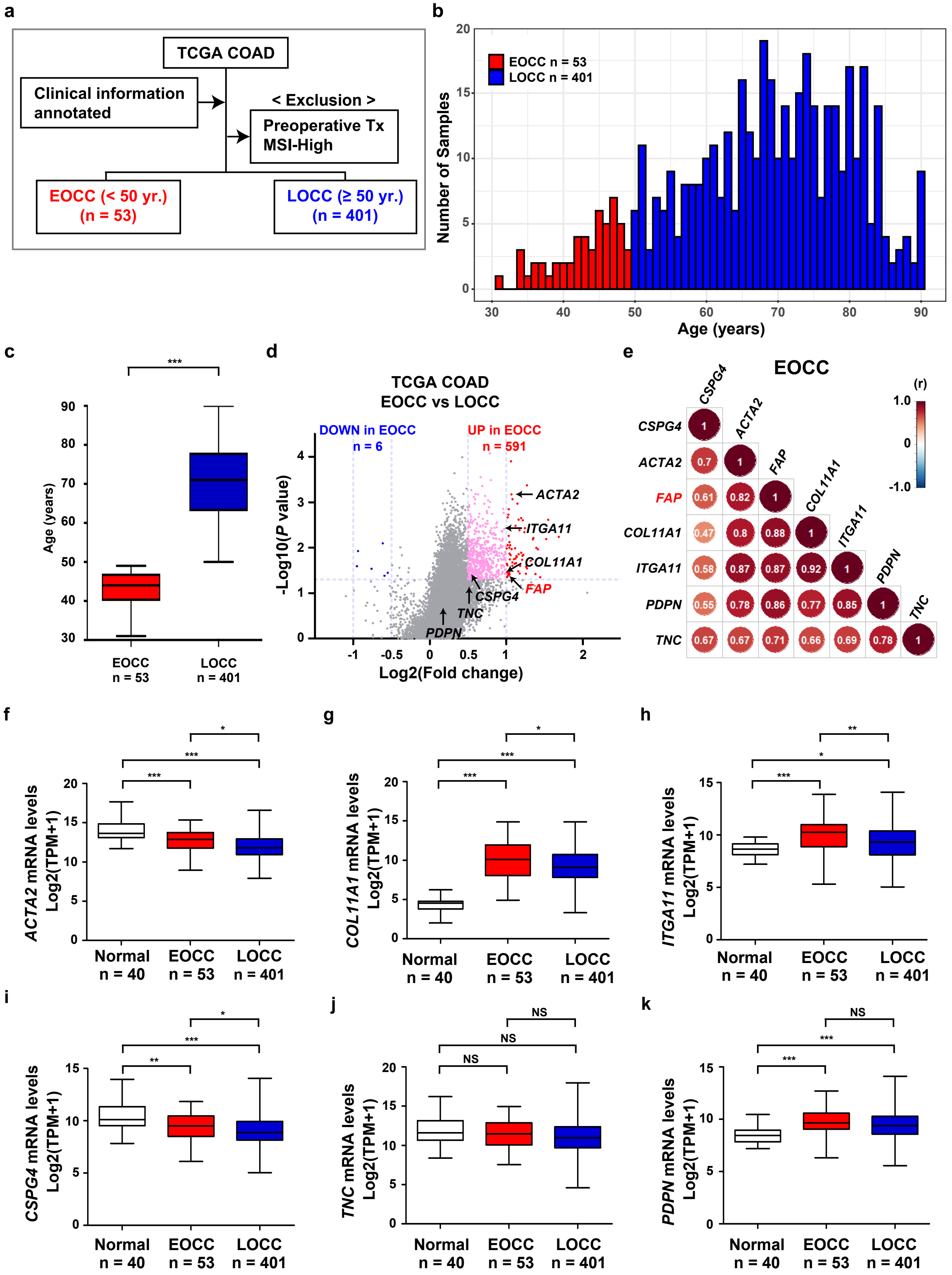

a
TCGA COAD
Clinical information annotated
< Exclusion >
Preoperative Tx
MSI-High
EOCC (< 50 yr.)
(n = 53)
LOCC (≥ 50 yr.)
(n = 401)
b
EOCC n = 53
LOCC n = 401
Number of Samples
Age (years)
c
Age (years)
EOCC n = 53
LOCC n = 401
d
TCGA COAD
EOCC vs LOCC
DOWN in EOCC n = 6
UP in EOCC n = 591
ACTA2
ITGA11
COL11A1
FAP
CSPG4
TNC
PDPN
-Log10(P value)
Log2(Fold change)
e
EOCC
CSPG4
ACTA2
FAP
COL11A1
ITGA11
PDPN
TNC
(r)
f
ACTA2 mRNA levels Log2(TPM+1)
Normal n = 40
EOCC n = 53
LOCC n = 401
g
COL11A1 mRNA levels Log2(TPM+1)
h
ITGA11 mRNA levels Log2(TPM+1)
i
CSPG4 mRNA levels Log2(TPM+1)
j
TNC mRNA levels Log2(TPM+1)
NS
k
PDPN mRNA levels Log2(TPM+1)

Supplementary Figure 1: CAF-related gene markers are upregulated in sporadic MSS EOCC a Flow chart showing the criteria for case selection in the TCGA COAD dataset and data categorization into EOCC (< 50 yr., n = 53) and LOCC (≥ 50 yr., n = 401). **b** A histogram showing the age distribution of MSS COAD dataset. **c** Boxplot showing age differences in EOCC and LOCC patients. **d** Volcano plot showing the 597 differentially expressed genes between EOCC and LOCC; 591 were upregulated (red dots: $Log_2(FC) \geq 1$; pink dots: $Log_2(FC) \geq 0.5$ and ≤ 1) and six were downregulated in EOCC (blue dots, $Log_2(FC) \leq 0.5$). **e** Hierarchical correlation plot showing the spearman correlation values among the mRNA levels of the seven CAF-related genes in the EOCC dataset. **f-k** Box plot charts showing *ACTA2* (**f**), *COL11A1* (**g**), *ITGA11* (**h**), *CSPG4* (**i**), *TNC* (**j**), and *PDPN* (**k**) mRNA levels ($Log_2(TPM+1)$) in normal tissues (white), EOCC tissue (red) and LOCC tissue (blue) using the TCGA COAD dataset. **p* <0.05, ***p* <0.01, ****p* <0.001. NS, not significant; FC, fold-change; TPM, transcripts per million; TCGA, The Cancer Genome Atlas; COAD, colon adenocarcinoma; Tx, treatment; MSI, microsatellite instability; EOCC, early-onset colon cancer; LOCC, late-onset colon cancer; (r), correlation coefficient.

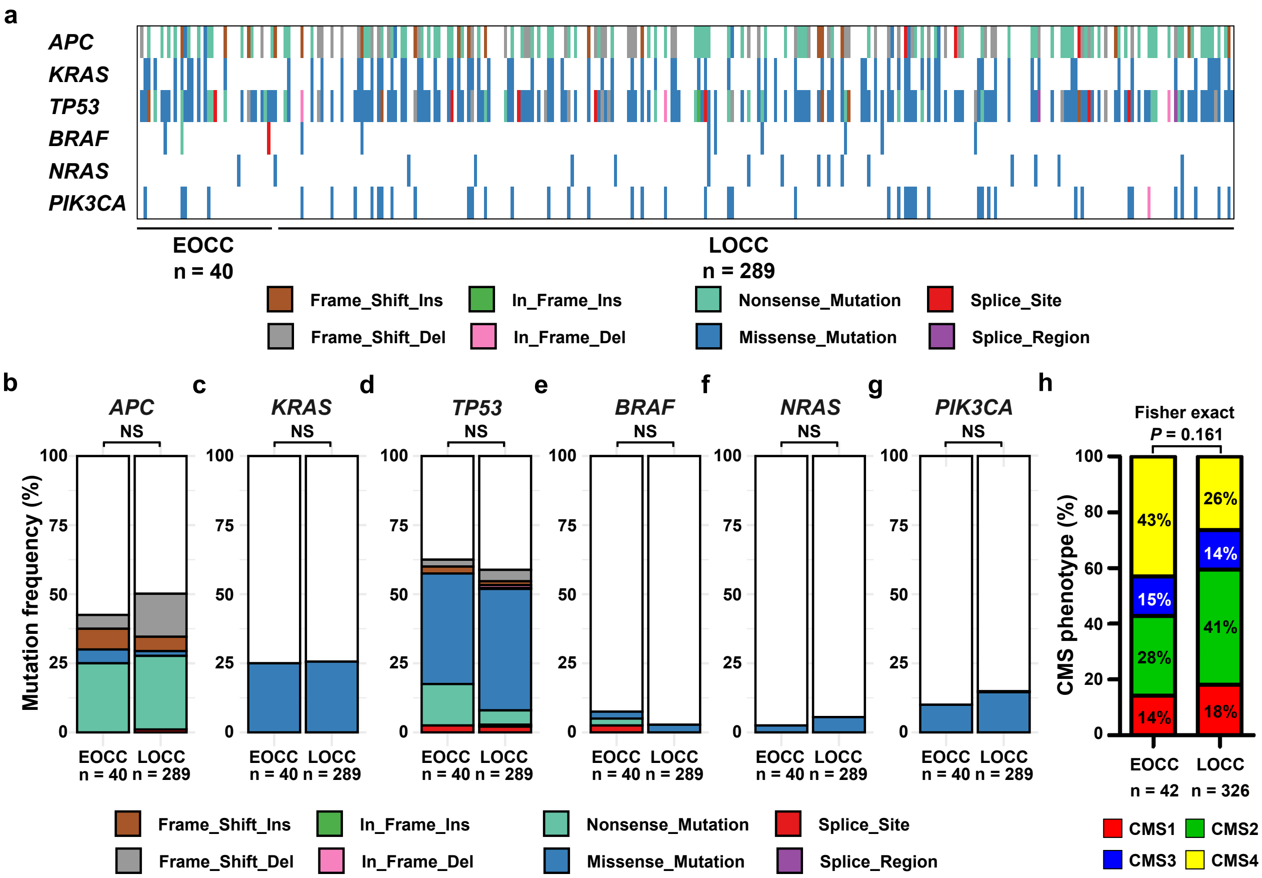
a
APC
KRAS
TP53
BRAF
NRAS
PIK3CA
EOCC
n = 40
LOCC
n = 289
Frame_Shift_Ins
In_Frame_Ins
Nonsense_Mutation
Splice_Site
Frame_Shift_Del
In_Frame_Del
Missense_Mutation
Splice_Region
b
c
d
e
f
g
h
APC
KRAS
TP53
BRAF
NRAS
PIK3CA
NS
Mutation frequency (%)
100
75
50
25
0
EOCC
n = 40
LOCC
n = 289
Fisher exact
P = 0.161
CMS phenotype (%)
100
80
60
40
20
0
43%
15%
28%
14%
26%
14%
41%
18%
EOCC
n = 42
LOCC
n = 326
CMS1
CMS2
CMS3
CMS4

Supplementary Figure 2: Mutation frequency of oncogene/tumor suppressor genes and transcriptomic levels of CAF-related genes between EOCC and LOCC in the TCGA COAD dataset a A heatmap showing the type of mutations of *APC*, *KRAS*, *TP53*, *BRAF*, *NRAS*, and *PIK3CA* in EOCC (n = 40), and LOCC (n = 289) using the TCGA COAD dataset. **b-g** Bar charts indicating mutation frequency of *APC* (**b**), *KRAS* (**c**), *TP53* (**d**), *BRAF* (**e**), *NRAS* (**f**), and *PIK3CA* (**g**) genes between EOCC and LOCC patients using the TCGA COAD dataset. **h** Stacked bar chart showing the proportion of CMS in EOCC and LOCC samples, respectively. EOCC, early-onset colon cancer; LOCC, late-onset colon cancer; NS, not significant; CMS, consensus molecular subtype.

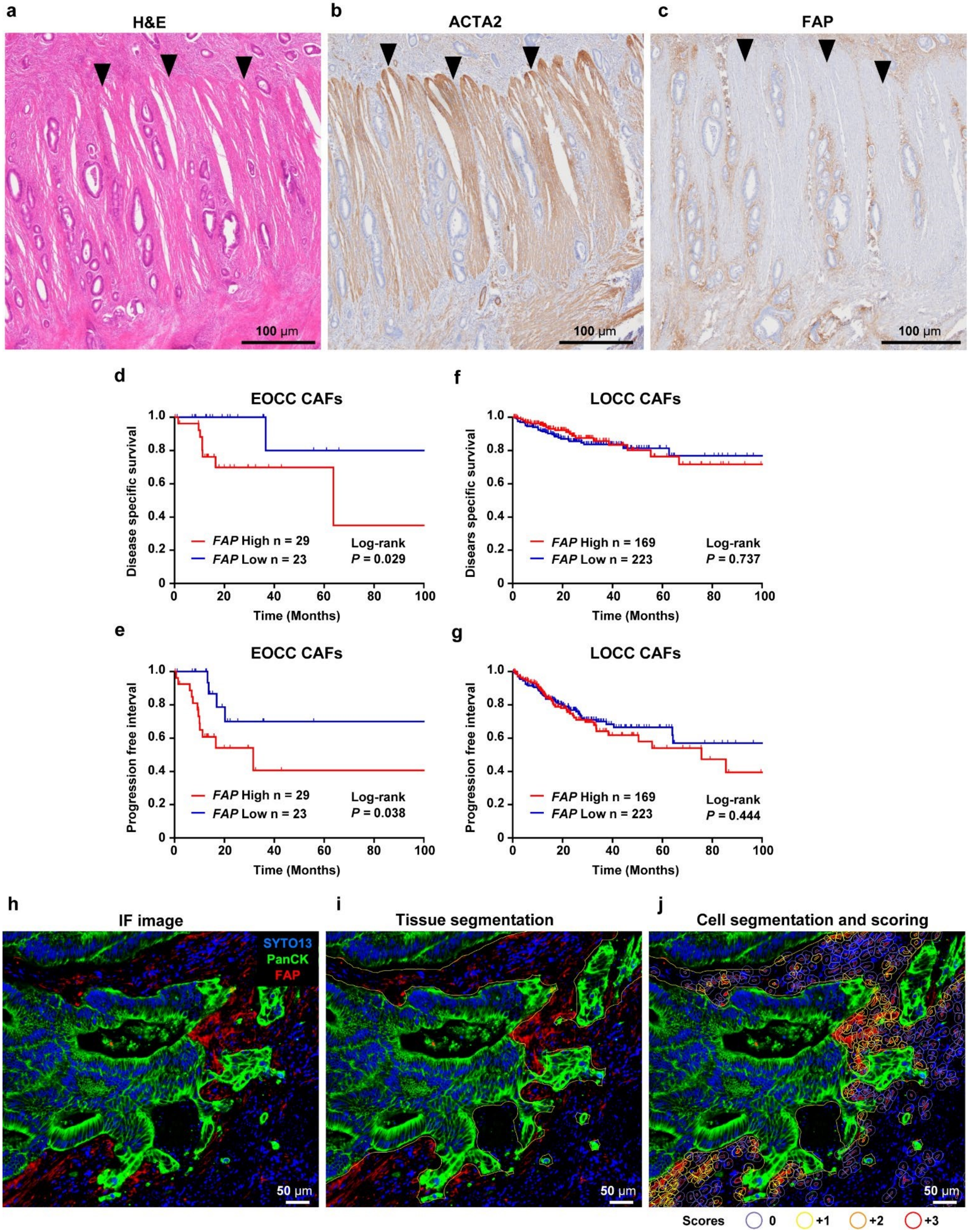

a
H&E
100 µm
b
ACTA2
100 µm
c
FAP
100 µm
d
EOCC CAFs
Disease specific survival
FAP High n = 29
FAP Low n = 23
Log-rank
P = 0.029
Time (Months)
e
EOCC CAFs
Progression free interval
FAP High n = 29
FAP Low n = 23
Log-rank
P = 0.038
Time (Months)
f
LOCC CAFs
Disears specific survival
FAP High n = 169
FAP Low n = 223
Log-rank
P = 0.737
Time (Months)
g
LOCC CAFs
Progression free interval
FAP High n = 169
FAP Low n = 223
Log-rank
P = 0.444
Time (Months)
h
IF image
SYTO13
PanCK
FAP
50 µm
i
Tissue segmentation
50 µm
j
Cell segmentation and scoring
50 µm
Scores 0 +1 +2 +3

Supplementary Figure 3: Staining patterns of ACTA2 and FAP in CC tissue and quantification of FAP protein levels a-c H&E staining (**a**) and IHC staining of ACTA2 (**b**) and FAP (**c**) using serial FFPE CC sections. Black arrowheads indicate smooth muscles in tumor tissues. **(d, e)** Kaplan-Meier survival curves of disease-specific survival (**d**) and progression-free interval (**e**) in EOCC patients that were stratified on high or low *FAP* mRNA levels in CAFs by the minimum *p*-value approach. **f, g** Kaplan-Meier survival curves of disease-specific survival (**f**) and progression -free interval (**g**) in LOCC patients that were stratified on high or low *FAP* mRNA levels in CAFs. **h-j** Automated process to quantify FAP protein levels in colon cancer tissue using an IF image (**h**) and Qupath software. The steps are as follows: tissue segmentation (**i**); cell segmentation and scoring (**j**). Yellow lines in **i** indicate borders between PanCK(+) and PanCK(-) segment. The intensity and proportion of FAP expression in the PanCK(-) segment were automatically scored in **j**. H&E, hematoxylin and eosin; ACTA2, Actin Alpha 2, Smooth Muscle; FAP, fibroblast activation protein; EOCC, early-onset colon cancer; CAF, cancer-associated fibroblast; LOCC, late-onset colon cancer; IF, immunofluorescence.

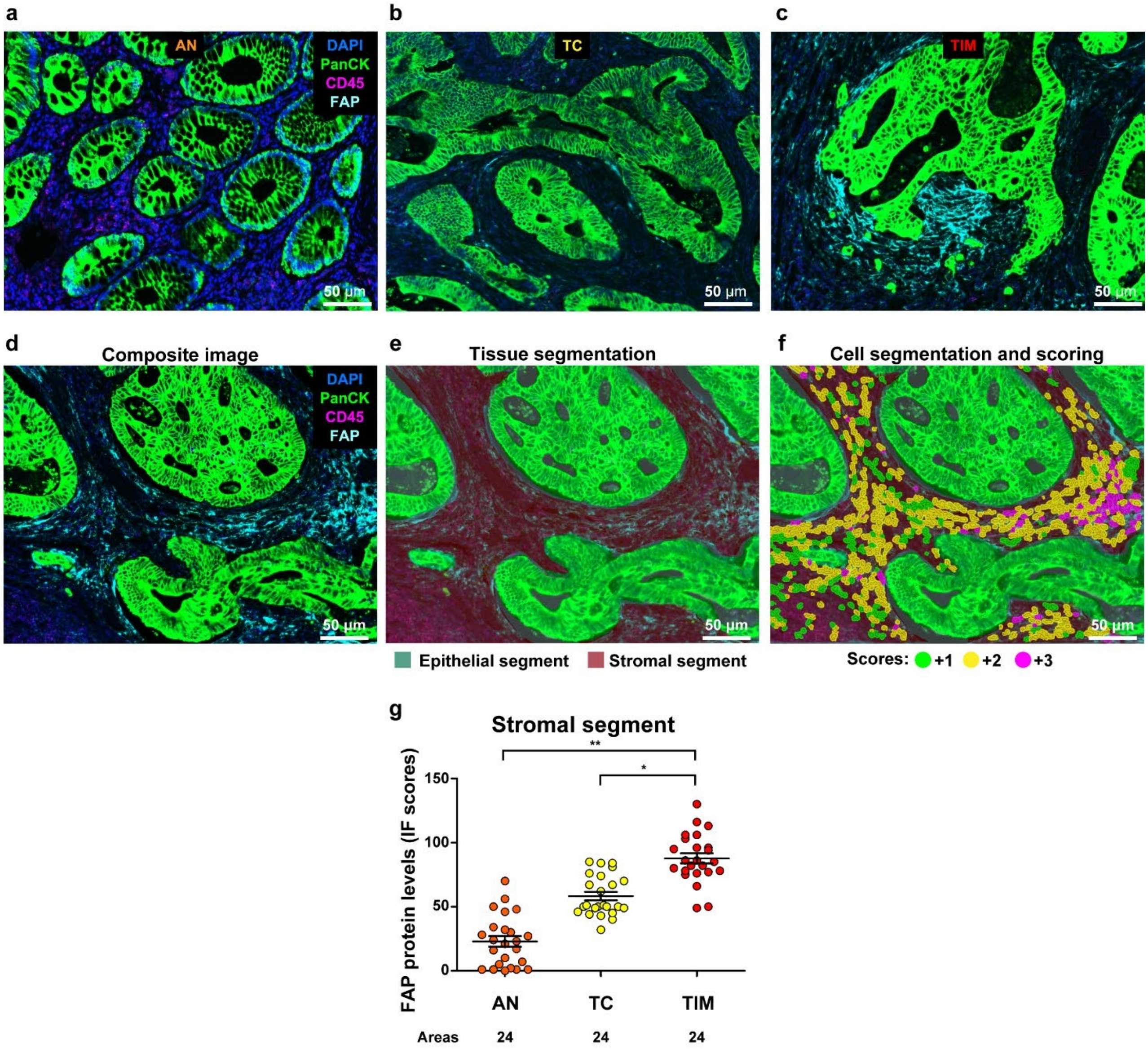

a
AN
DAPI
PanCK
CD45
FAP
50 µm
b
TC
50 µm
c
TIM
50 µm
d
Composite image
DAPI
PanCK
CD45
FAP
50 µm
e
Tissue segmentation
50 µm
Epithelial segment
Stromal segment
f
Cell segmentation and scoring
50 µm
Scores: +1 +2 +3
g
Stromal segment
**
*
FAP protein levels (IF scores)
150
100
50
0
AN
TC
TIM
Areas
24
24
24

Supplementary Figure 4: FAP protein expression in colon cancer tumor and TME

a-c Representative mIF images of AN (**a**), TC (**b**), and TIM (**c**) of colon cancer tissues. Tissue samples were stained using the Opal multiplex staining kit. DAPI (blue); PanCK (green, Opal 690); CD45 (magenta, Opal 540); FAP (cyan, Opal 650). **d-f** The intensity and proportion of FAP protein expression in stromal tissues were automatically scored. Shown are the composite images **(d),** tissue segmentation (**e**), and cell segmentation and scoring (**f**) performed by InForm software. The green mask in figure **e** is for PanCK(+) epithelial segment and the red mask is for PanCK(-) stromal segment. **g** Box plot chart showing the comparison of FAP protein levels (IF scores) in PanCK(-) stromal segment among the considered areas (AN, TC, and TIM). **p* <0.05; ***p* < 0.01.

AN, adjacent normal; TC, tumor center; TIM, tumor invasive margin; IF immunofluorescence.

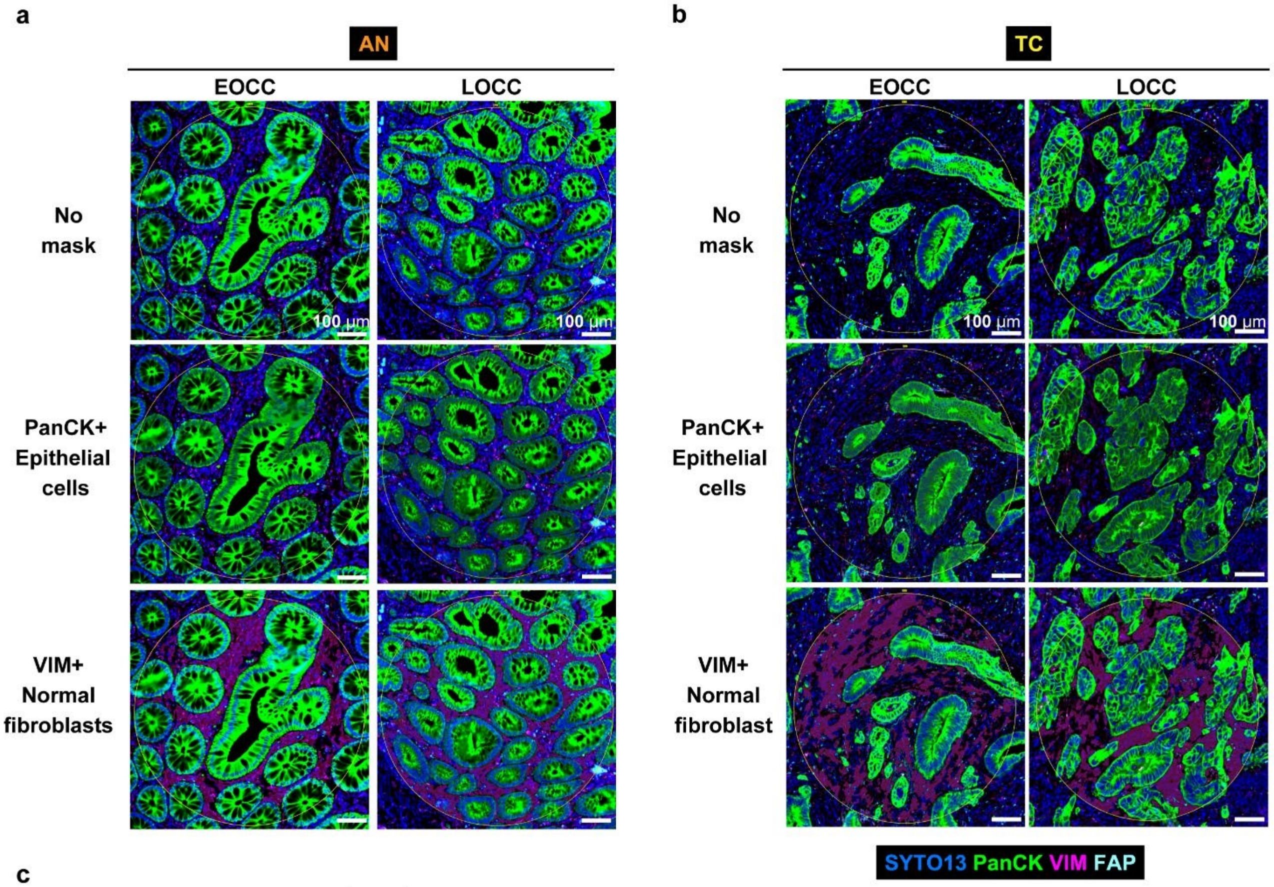

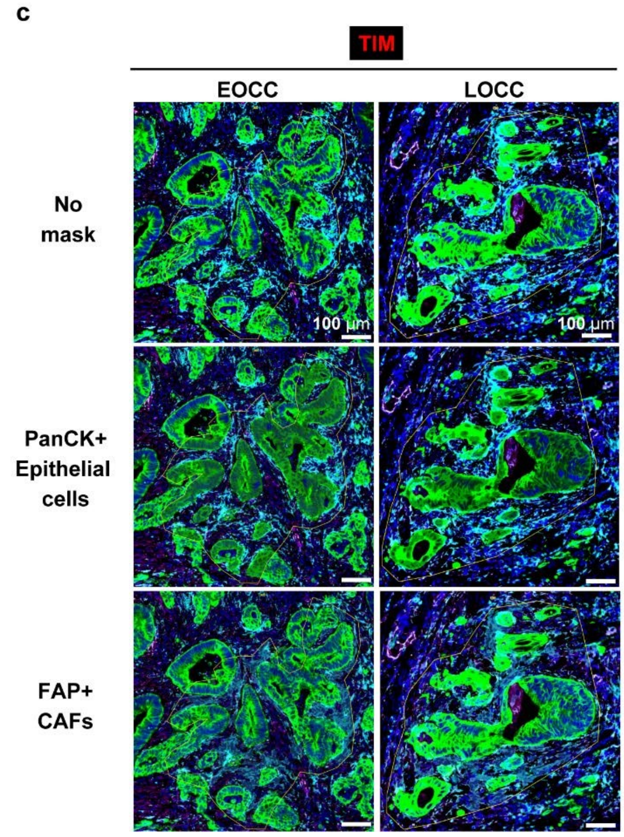

d

Number of AOIs

	AN		TC		TIM	
	EOCC	LOCC	EOCC	LOCC	EOCC	LOCC
PanCK+ Epithelial cells	4	4	12	12	12	12
VIM+ Normal fibroblasts	4	4	12	12	0	0
FAP+ CAFs	0	0	0	0	12	12

Supplementary Figure 5: Selections of the AOIs in NGDSP.

a-c Representative pictures of unmasked or masked AOIs at AN (**a**), TC (**b**), and TIM (**c**) in EOCC or LOCC tumor samples. SYTO13 (blue, DNA); pan-cytokeratin (green, PanCK(+)); vimentin (magenta, VIM(+)); fibroblast activation protein (cyan, FAP(+)). **d** Table showing the numbers of sequenced AOIs in each histological area and sample type. AN, adjacent normal; EOCC, early-onset colon cancer; LOCC, late-onset colon cancer; TC, tumor center; TIM, tumor invasive margin; CAF, cancer-associated fibroblast; AOI, area of illumination.

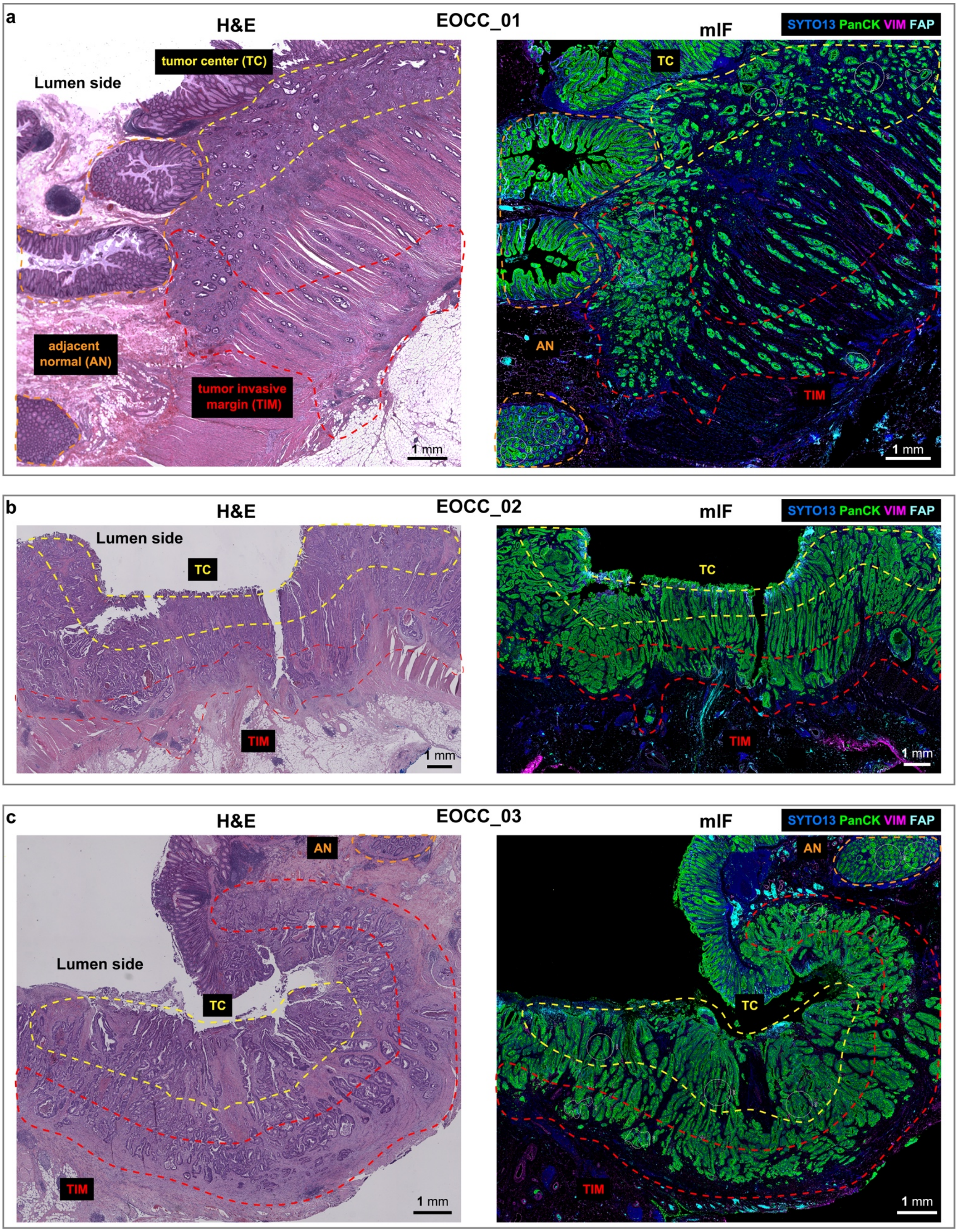
a
H&E
EOCC_01
mIF
SYTO13 PanCK VIM FAP
tumor center (TC)
Lumen side
adjacent normal (AN)
tumor invasive margin (TIM)
1 mm
TC
AN
TIM
1 mm
b
H&E
EOCC_02
mIF
SYTO13 PanCK VIM FAP
Lumen side
TC
TIM
1 mm
TC
TIM
1 mm
c
H&E
EOCC_03
mIF
SYTO13 PanCK VIM FAP
AN
Lumen side
TC
TIM
1 mm
AN
TC
TIM
1 mm

Supplementary Figure 6: Definition of AN, TC, and TIM and the corresponding ROI selections of EOCC_01, EOCC_02, and EOCC_03 tissues

a-c Left panel: H&E staining of colon cancer samples; Right panel: mIF staining using morphological markers including SYTO13 (blue, DNA), PanCK(+) (green), VIM(+) (magenta), and FAP(+) (cyan) in the sequential FFPE slide. The selected ROIs for NGDSP were marked with a circle. The histological areas were defined and marked with dotted lines: AN (orange), TC (yellow), and TIM (red). (**a**), EOCC_01; (**b**), EOCC_02; (**c**), EOCC_03. EOCC, early-onset colon cancer; H&E, hematoxylin and eosin; mIF, multiplex immunofluorescence, AN, adjacent normal; ROI, regions of interest; TC, tumor center; TIM, tumor invasive margin.

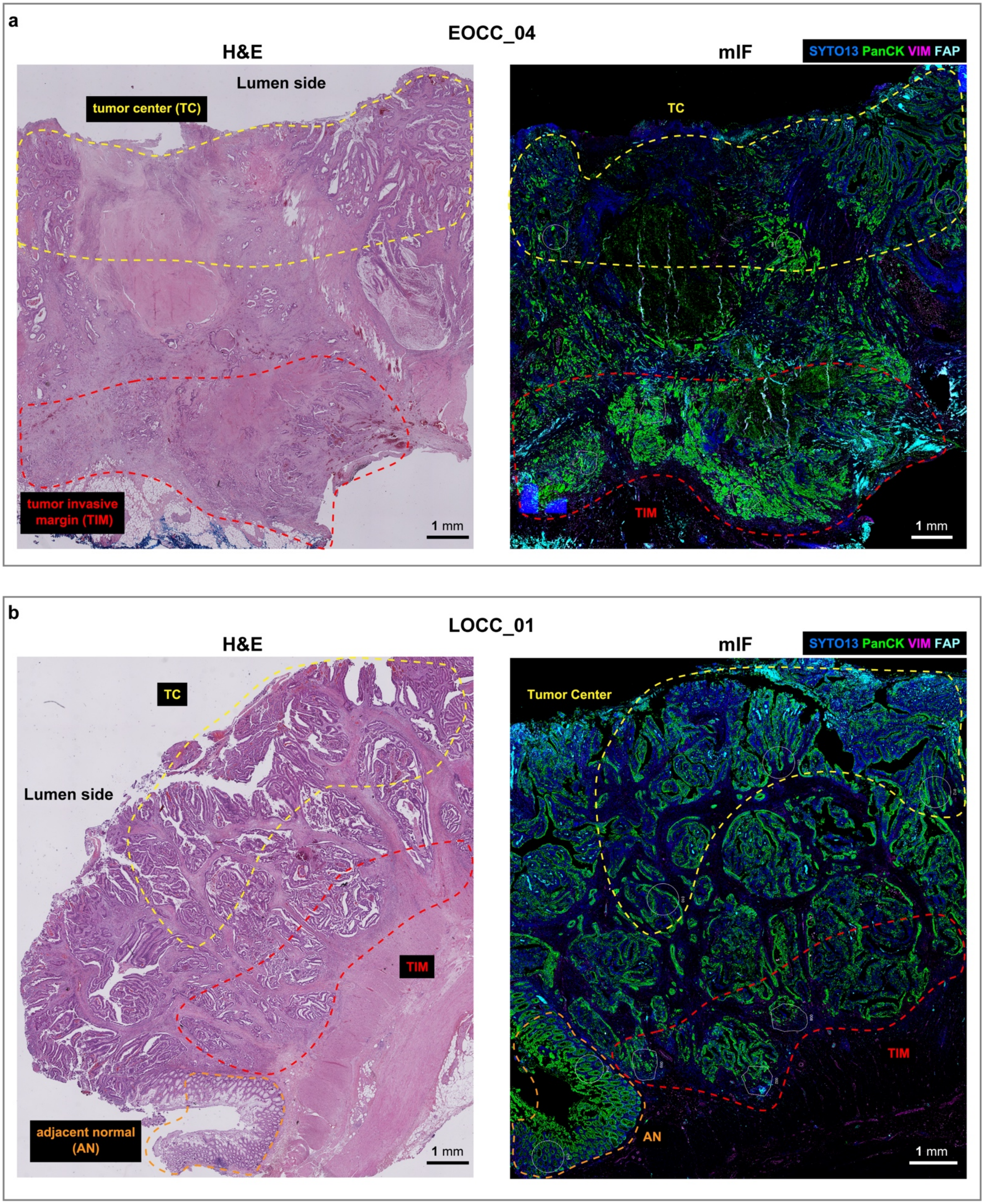
a
EOCC_04
H&E
mIF
SYTO13 PanCK VIM FAP
Lumen side
tumor center (TC)
tumor invasive margin (TIM)
1 mm
TC
TIM
1 mm
b
LOCC_01
H&E
mIF
SYTO13 PanCK VIM FAP
TC
Lumen side
TIM
adjacent normal (AN)
1 mm
Tumor Center
TIM
AN
1 mm

Supplementary Figure 7: Definition of AN, TC, and TIM and corresponding ROI selections of EOCC_04 and LOCC_01 tissues

a, b Left panel: H&E staining of CC samples; Right panel: mIF staining using morphological markers including SYTO13 (blue, DNA), PanCK(+) (green), VIM(+) (magenta), and FAP(+) (cyan) in the sequential slide. The selected ROIs for GeoMx DSP were marked with a circle. The histological areas were defined and marked with dotted lines: AN (orange), TC (yellow), and TIM (red). (**a**), EOCC_04; (**b**), LOCC_01. ROI, region of interest; EOCC, early-onset colon cancer; H&E, hematoxylin and eosin; mIF, multiplex immunofluorescence, AN, adjacent normal; TC, tumor center; TIM, tumor invasive margin; LOCC, late-onset colon cancer.

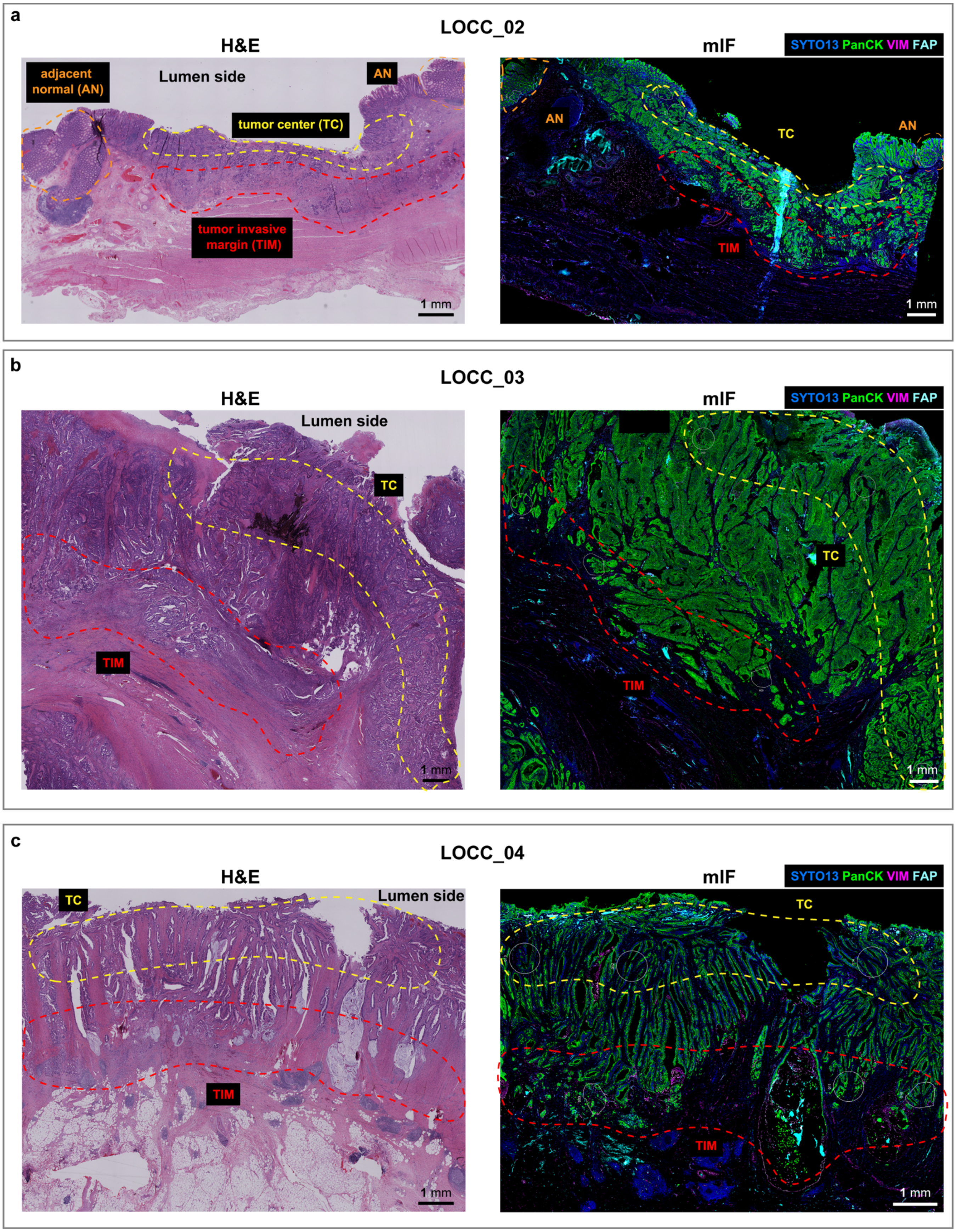

a
LOCC_02
H&E
mIF
SYTO13 PanCK VIM FAP
adjacent normal (AN)
Lumen side
AN
tumor center (TC)
tumor invasive margin (TIM)
1 mm
AN
TC
AN
TIM
1 mm
b
LOCC_03
H&E
Lumen side
TC
TIM
1 mm
mIF
SYTO13 PanCK VIM FAP
TC
TIM
1 mm
c
LOCC_04
H&E
TC
Lumen side
TIM
1 mm
mIF
SYTO13 PanCK VIM FAP
TC
TIM
1 mm

Supplementary Figure 8: Definition of AN, TC, and TIM and corresponding ROI selections of LOCC_02, LOCC_03, and LOCC_04 tissues

a-c Left panel: H&E staining of colon cancer samples; Right panel: mIF staining using morphological markers including SYTO13 (blue, DNA), PanCK(+) (green), VIM(+) (magenta), and FAP(+) (cyan) in the sequential slide. The selected ROIs for NGDSP were marked with a circle. The histological areas were defined and marked with dotted lines: AN (orange), TC (yellow), and TIM (red). (**a**), LOCC_02; (**b**), LOCC_03; (**c**), LOCC_04. ROI, region of interest; LOCC, late-onset colon cancer; H&E, hematoxylin and eosin; mIF, multiplex immunofluorescence, AN, adjacent normal; TC, tumor center; TIM, tumor invasive margin.

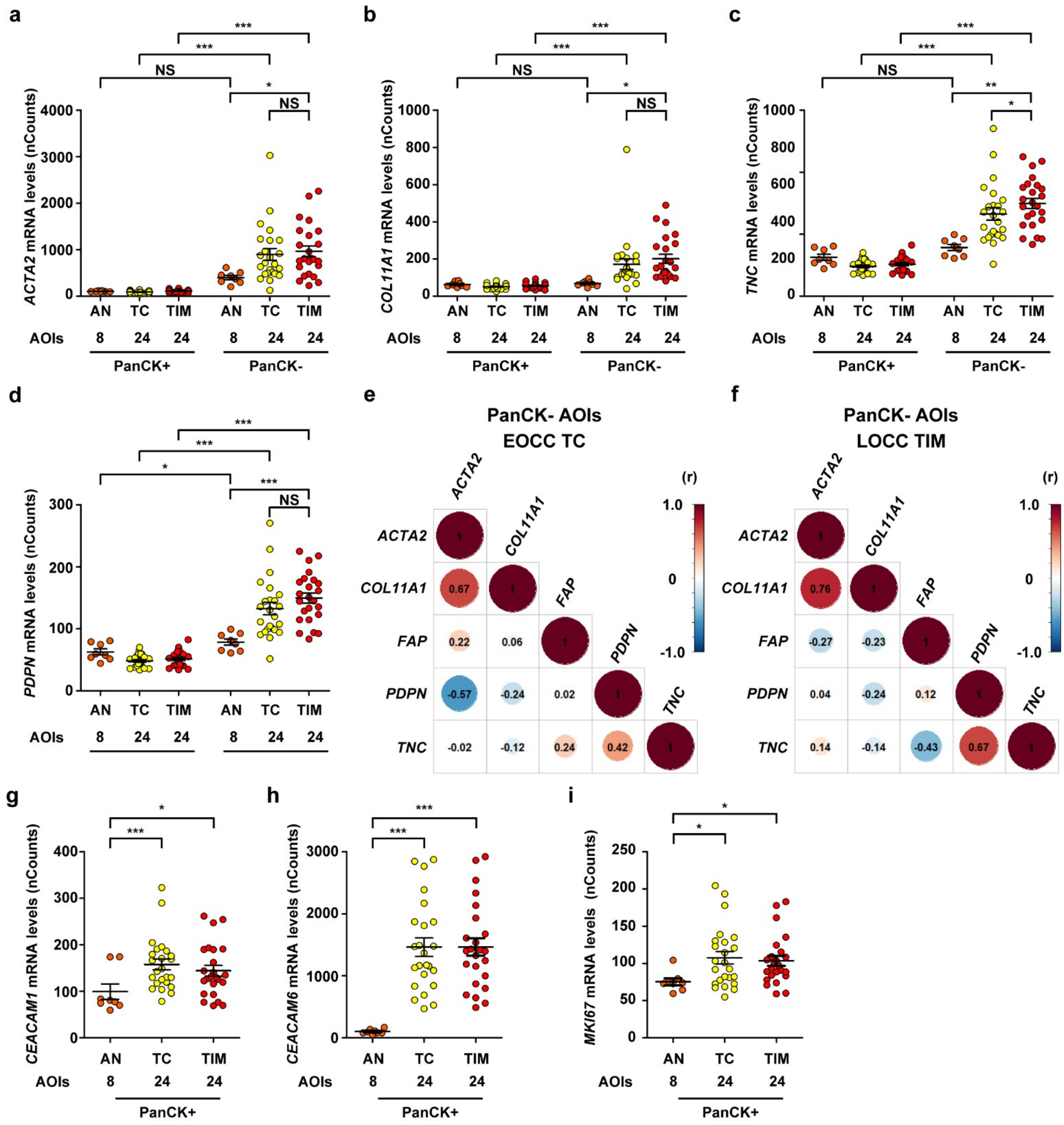

a
ACTA2 mRNA levels (nCounts)
NS
***
***
*
NS
AN TC TIM AN TC TIM
AOIs 8 24 24 8 24 24
PanCK+
PanCK-
b
COL11A1 mRNA levels (nCounts)
NS
***
***
*
NS
c
TNC mRNA levels (nCounts)
NS
***
***
**
*
d
PDPN mRNA levels (nCounts)
*
***
***
***
NS
e
PanCK- AOIs
EOCC TC
ACTA2
COL11A1
FAP
PDPN
TNC
(r)
1.0
0
-1.0
1
0.67
0.22
0.06
-0.57
-0.24
0.02
-0.02
-0.12
0.24
0.42
f
PanCK- AOIs
LOCC TIM
0.76
-0.27
-0.23
0.04
-0.24
0.12
0.14
-0.14
-0.43
0.67
g
CEACAM1 mRNA levels (nCounts)
***
*
AN TC TIM
AOIs 8 24 24
PanCK+
h
CEACAM6 mRNA levels (nCounts)
***
***
i
MKI67 mRNA levels (nCounts)
*
*

Supplementary Figure 9: **Comparisons of transcriptomic levels of CAF-related genes, *CEACAMs*, and *MKI67* in each area were obtained by NGDSP analysis**

a-d Quantification of *ACTA2* (**a**), *COL11A1* (**b**), *TNC* (**c**), and *PDPN* (**d**) mRNA levels (nCounts) in each AOI obtained by GeoMx DSP is shown. **e, f** Correlation plots indicating the spearman correlation values for five CAF-related genes at PanCK(-) AOIs in EOCC TC (**e**) and at PanCK(-) AOIs in LOCC TIM (**f**). **g-i** Quantification of *CEACAM1* (**g**), *CEACAM6* (**h**), and *MKI67* (**i**) mRNA levels (nCounts) in each AOI obtained by NGDSP. *$p < 0.05$; **$p < 0.01$; ***$p < 0.001$. nCounts, normalized counts; NS, not significant; AOI, area of illumination; AN, adjacent normal; TC, tumor center; TIM, tumor invasive margin; EOCC, early-onset colon cancer; LOCC, late-onset colon cancer; (r), correlation coefficient.

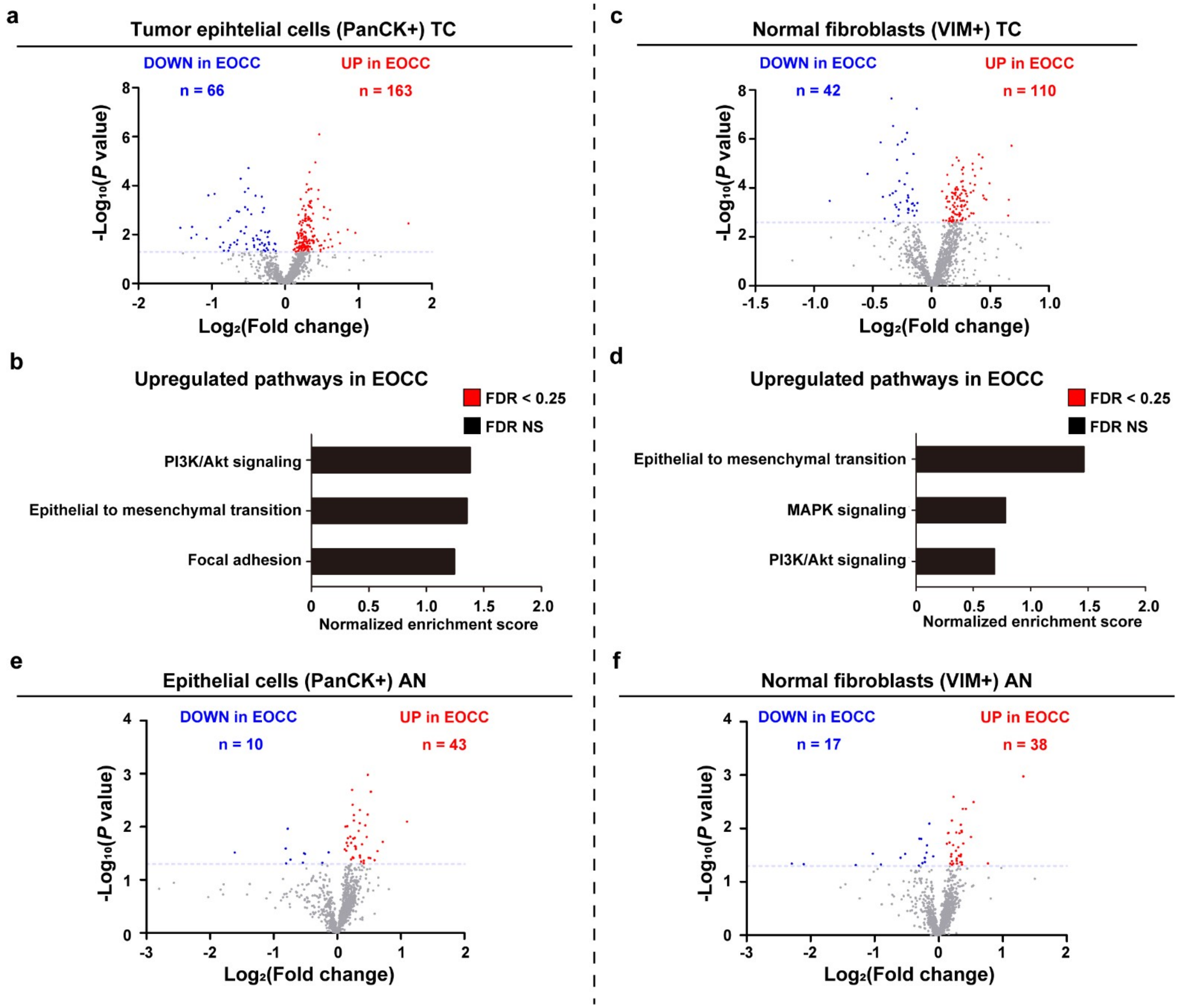

a
Tumor epihtelial cells (PanCK+) TC
DOWN in EOCC
n = 66
UP in EOCC
n = 163
-Log10(P value)
Log2(Fold change)
b
Upregulated pathways in EOCC
FDR < 0.25
FDR NS
PI3K/Akt signaling
Epithelial to mesenchymal transition
Focal adhesion
Normalized enrichment score
c
Normal fibroblasts (VIM+) TC
DOWN in EOCC
n = 42
UP in EOCC
n = 110
-Log10(P value)
Log2(Fold change)
d
Upregulated pathways in EOCC
FDR < 0.25
FDR NS
Epithelial to mesenchymal transition
MAPK signaling
PI3K/Akt signaling
Normalized enrichment score
e
Epithelial cells (PanCK+) AN
DOWN in EOCC
n = 10
UP in EOCC
n = 43
-Log10(P value)
Log2(Fold change)
f
Normal fibroblasts (VIM+) AN
DOWN in EOCC
n = 17
UP in EOCC
n = 38
-Log10(P value)
Log2(Fold change)

Supplementary Figure 10: Comparisons of mRNA levels of each phenotype at TC and AN between EOCC and LOCC

a Volcano plot showing the DEGs in PanCK(+) tumor epithelial cells at TC in EOCC versus LOCC, obtained by GeoMx DSP analysis. Of the 229 DEGs, 163 were upregulated (red dots) and 66 were downregulated (blue dots) in EOCC. **b** Bar chart showing normalized enrichment scores of top ranked pathways that were upregulated in PanCK(+) tumor epithelial cells AOIs of EOCC compared to LOCC. Black bars indicate NS *p*-values with an FDR ≥ 0.25. **c** Volcano plot showing the DEGs in VIM(+) normal fibroblasts at TC comparing between EOCC and LOCC obtained by GeoMx DSP analysis. Of the 152 DEGs, 110 were upregulated (red dots) and 42 were downregulated (blue dots) in EOCC. **d** Bar chart showing normalized enrichment scores of top ranked pathways that were upregulated in VIM(+) fibroblasts AOIs of EOCC compared to LOCC. Black bars indicate NS *p*-values with an FDR ≥ 0.25. **e** Volcano plot showing the DEGs in PanCK(+) epithelial cells at AN comparing between EOCC and LOCC obtained by NGDSP analysis. Of the 53 DEGs, 43 were upregulated (red dots) and 10 were downregulated (blue dots) in EOCC. **f** Volcano plot showing the DEGs in VIM(+) normal fibroblasts at AN comparing between EOCC and LOCC obtained by GeoMx DSP analysis. Of the 55 DEGs, 38 were upregulated (red dots) and 17 were downregulated (blue dots) in EOCC. AOI, area of illumination; TC, tumor center; EOCC, early-onset colon cancer; FDR, false discovery rate; NS, not significant; VIM, vimentin; AN, adjacent normal.

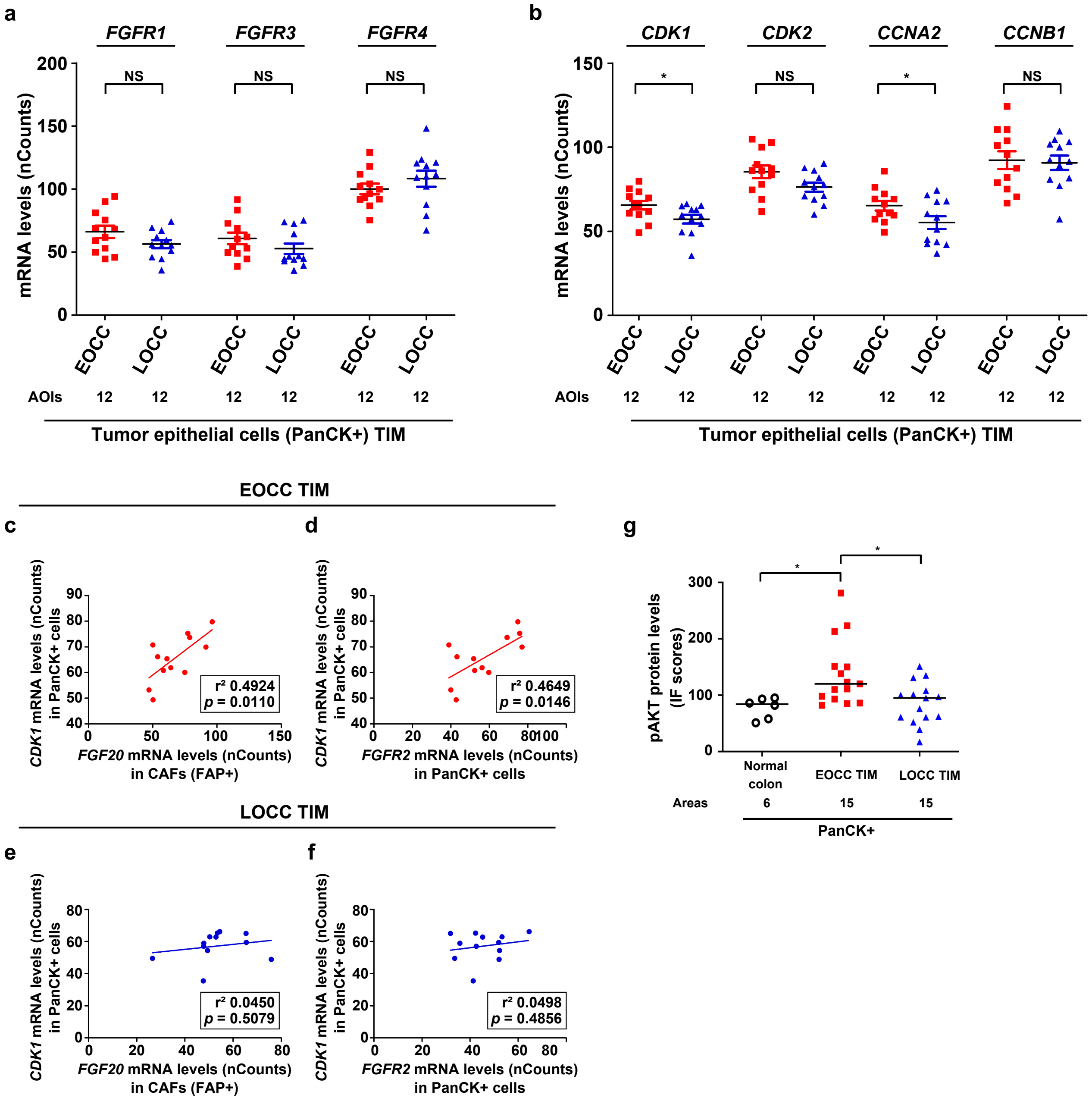

a
FGFR1
FGFR3
FGFR4
NS
mRNA levels (nCounts)
EOCC
LOCC
AOIs
12
Tumor epithelial cells (PanCK+) TIM
b
CDK1
CDK2
CCNA2
CCNB1
*
NS
EOCC TIM
c
d
CDK1 mRNA levels (nCounts) in PanCK+ cells
FGF20 mRNA levels (nCounts) in CAFs (FAP+)
FGFR2 mRNA levels (nCounts) in PanCK+ cells
r² 0.4924
p = 0.0110
r² 0.4649
p = 0.0146
LOCC TIM
e
f
r² 0.0450
p = 0.5079
r² 0.0498
p = 0.4856
g
pAKT protein levels (IF scores)
Normal colon
EOCC TIM
LOCC TIM
Areas
6
15
15
PanCK+

Supplementary Figure 11: Comparisons of mRNA levels in FGFR families and cell cycle genes obtained by GeoMx DSP analysis

a Scatter plot showing the *FGFR1*, *FGFR3*, and *FGFR4* mRNA levels (nCounts) between EOCC and LOCC in each AOI of PanCK(+) tumor epithelial cells TIM. **b** Scatter plot showing the *CDK1*, *CDK2*, *CCNA2*, and *CCNB1* mRNA levels (nCounts) between EOCC and LOCC in each AOI of PanCK(+) tumor epithelial cells TIM. **c, d** Plot showing the correlation values between the mRNA levels (nCounts) of *CDK1* in PanCK(+) tumor epithelial cells and *FGF20* in FAP(+) CAFs (**c**) and *FGFR2* in PanCK(+) tumor epithelial cells (**d**) at EOCC TIM. **(e, f)** Plot showing the correlation values between the mRNA levels (nCounts) of *CDK1* in PanCK(+) tumor epithelial cells and *FGF20* in FAP(+) CAFs (**e**) and *FGFR2* in PanCK(+) tumor epithelial cells (**f**) at LOCC TIM. **g** Quantification of pAKT protein levels in each area of normal colon (white), EOCC TIM (red), and LOCC TIM (blue) tissues using IF scores which were calculated with InForm software. *p <0.05. nCounts, normalized counts; NS, not significant; EOCC, early-onset colon cancer; LOCC, late-onset colon cancer; AOI, area of illumination, TIM, tumor invasive margin; CAF, cancer-associated fibroblast; r^2, coefficient of determination; IF, immunofluorescence.

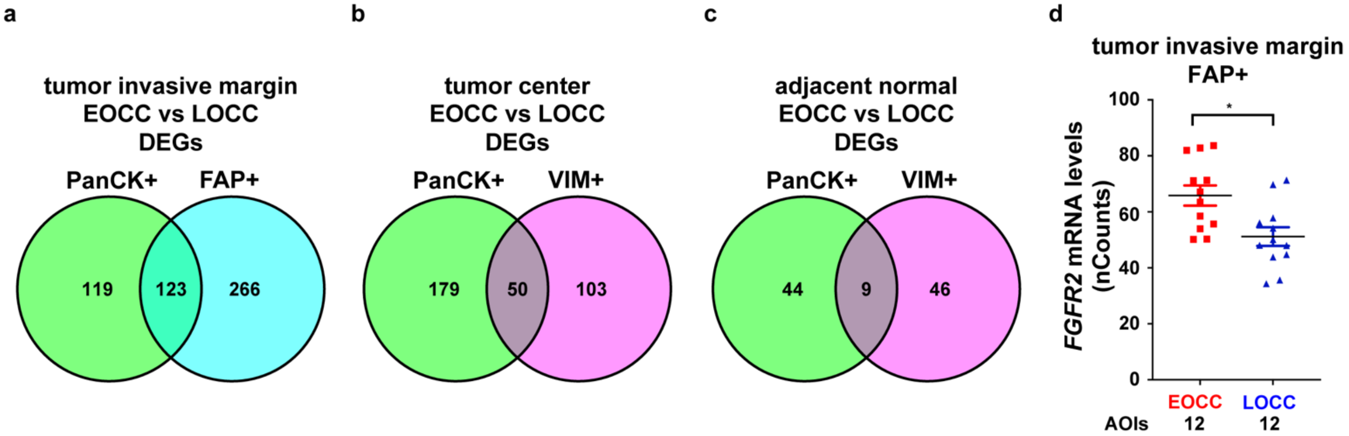
a
tumor invasive margin
EOCC vs LOCC
DEGs
PanCK+
FAP+
119
123
266
b
tumor center
EOCC vs LOCC
DEGs
PanCK+
VIM+
179
50
103
c
adjacent normal
EOCC vs LOCC
DEGs
PanCK+
VIM+
44
9
46
d
tumor invasive margin
FAP+
FGFR2 mRNA levels
(nCounts)
100
80
60
40
20
0
*
EOCC
LOCC
AOIs
12
12

Supplementary Figure 12: Overlapped DEGs in the PanCK(+) and FAP(+) cells between EOCC and LOCC

a-c Venn diagrams showing the overlapped DEGs in the PanCK(+) and FAP(+) cells obtained by the comparison between EOCC and LOCC at TIM (**a**), TC (**b**), and AN (**c**) areas. **d** Scatter plot showing the *FGFR2* mRNA levels (nCounts) in FAP(+) cells at EOCC and LOCC TIM. *$p < 0.05$. EOCC, early-onset colon cancer; TIM, tumor invasive margin; TC, tumor center; AN: adjacent normal; LOCC, late-onset colon cancer; DEGs, differentially expressed genes; nCounts, normalized counts; AOIs, area of illumination.

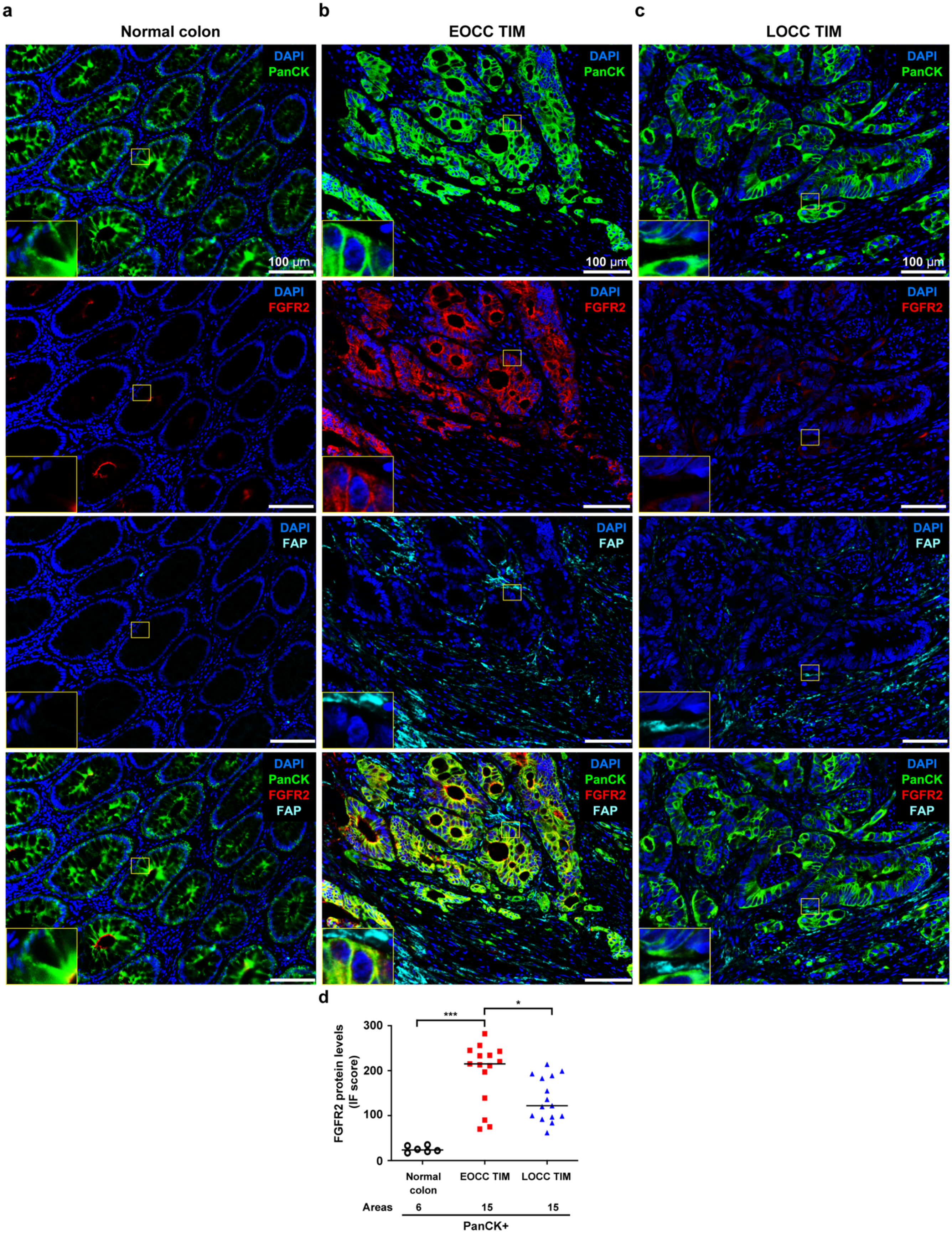
a
Normal colon
b
EOCC TIM
c
LOCC TIM
DAPI
PanCK
FGFR2
FAP
100 µm
d
FGFR2 protein levels (IF score)
***
*
0
100
200
300
Normal colon
EOCC TIM
LOCC TIM
Areas
6
15
15
PanCK+

Supplementary Figure 13: Multiplex immunofluorescence images showing FGFR2 staining patterns at tumor epithelial cells of EOCC tumor invasive margin

a-c Representative mIF images of normal colon (**a**), EOCC tumor invasive margin **(**TIM, **b**) and LOCC TIM (**c**). Tissue samples were stained using the Opal kit: DAPI (blue), PanCK (green, Opal 690); FGFR2 (red, Opal 620); FAP (cyan, Opal 650). The insets represent the magnified image of the yellow box of each picture. **d** Quantification of FGFR2 protein levels in each area of normal colon (white), EOCC (red), and LOCC (blue) tissues using IF scores which were calculated with InForm software. *p <0.05, ***p <0.001. EOCC, early-onset colon cancer; TIM, tumor invasive margin; LOCC, late-onset colon cancer; mIF, multiplex immunofluorescence.

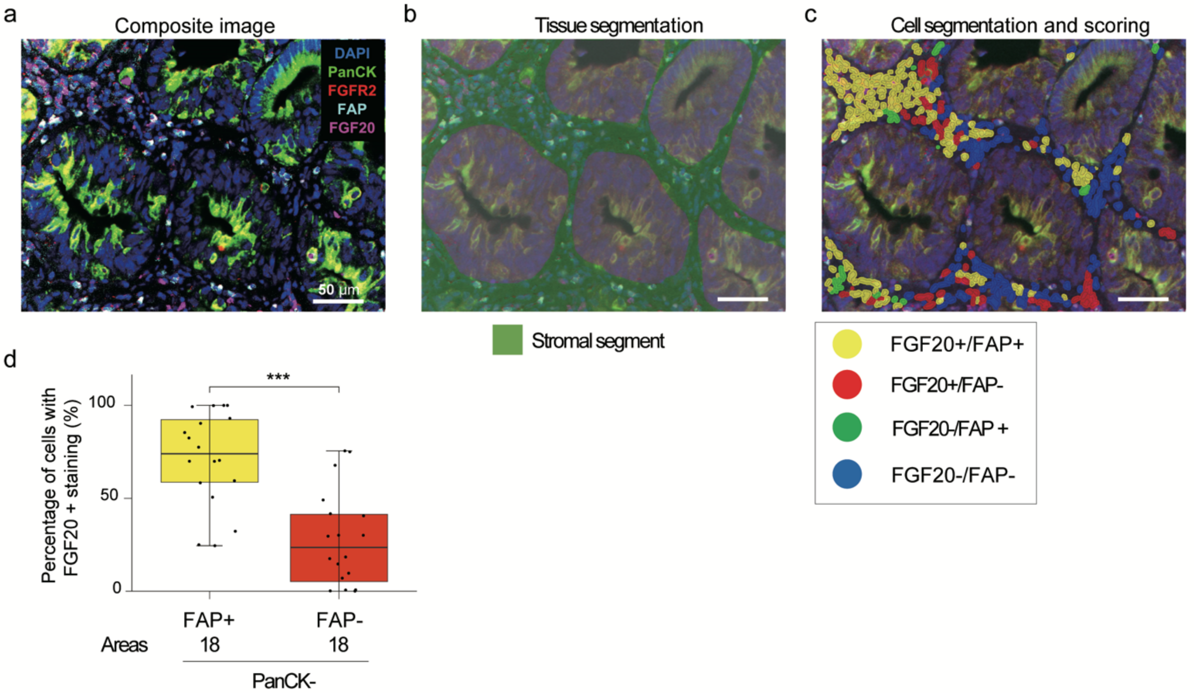
a
Composite image
DAPI
PanCK
FGFR2
FAP
FGF20
50 µm
b
Tissue segmentation
Stromal segment
c
Cell segmentation and scoring
FGF20+/FAP+
FGF20+/FAP-
FGF20-/FAP +
FGF20-/FAP-
d
Percentage of cells with FGF20 + staining (%)
100
50
0
***
FAP+
18
FAP-
18
Areas
PanCK-

Supplementary Figure 14: Comparison of FGF20 protein levels between FAP(+) and FAP(-) cells in stromal segment of colon cancer samples.

a-c Images shown are the composite image (**a**), tissue segmentation (**b**), and cell segmentation and scoring (**c**) performed by InForm software. The green mask in **b** is for PanCK(-) stromal segment. Tissue samples were stained using the Opal multiplex staining kit. DAPI (blue); PanCK (green, Opal 690); FGFR2 (red, Opal 620); FGF20 (magenta, Opal 540); FAP (cyan, Opal 650). **d** Box plot chart showing the proportion of FGF20 protein levels between FAP(+) and FAP(-) cells in PanCK(-) stromal segment. ***$p < 0.001$.

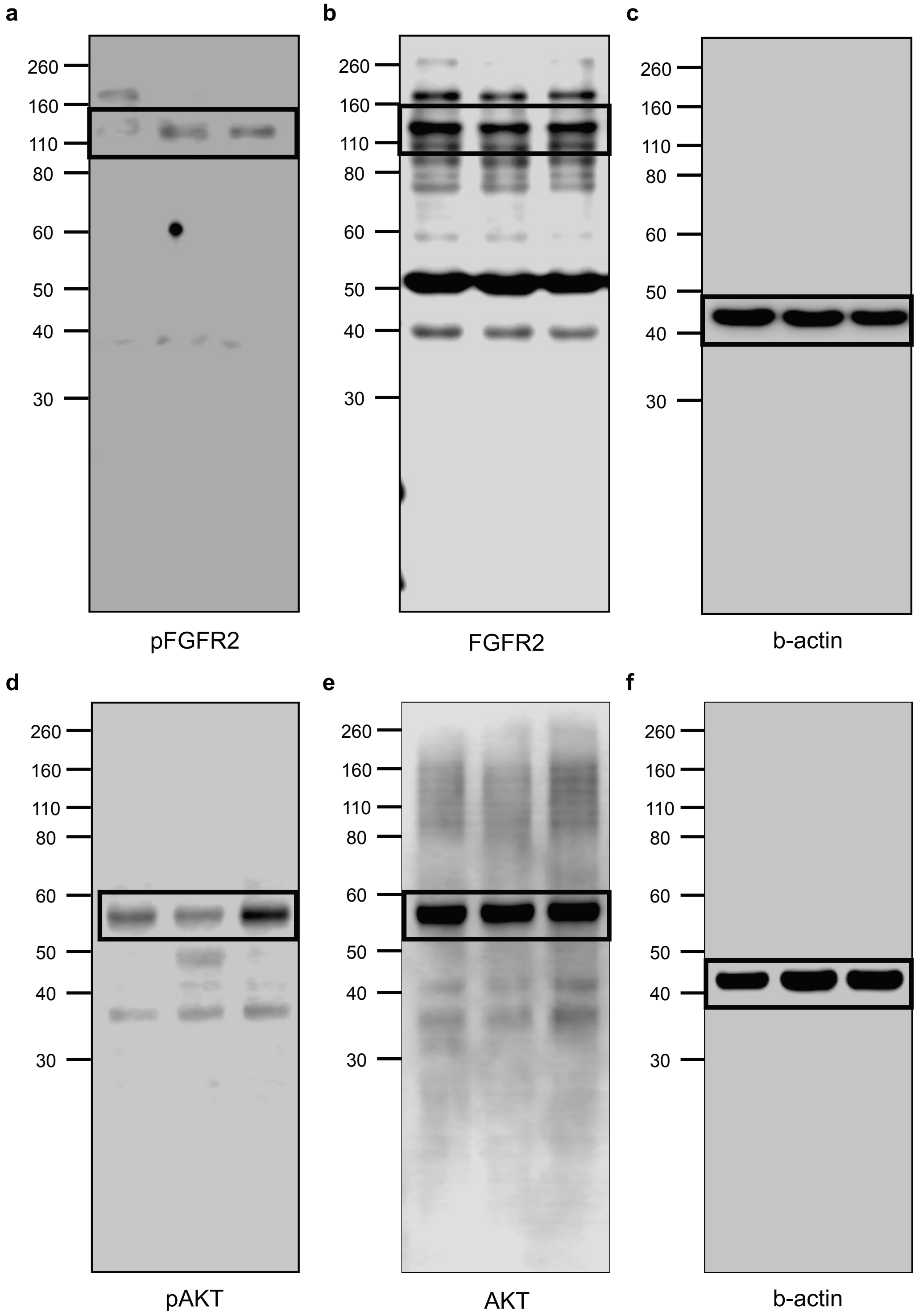
a
b
c
260
160
110
80
60
50
40
30
pFGFR2
FGFR2
b-actin
d
e
f
pAKT
AKT
b-actin

Supplementary Figure 15: Uncropped images of western blotting assay

a-f Uncropped images of western blotting for phosphorylated FGFR2 (pFGFR2, **a**), FGFR2 (**b**), β-actin (**c, f**), phosphorylated AKT (pAKT, **d**), and AKT (**e**). All the images refer to Fig. 7f.

Supplementary Table 1. List of the datasets used in the study.

Datasets accession number/name	Type of NGS dataset	Biomolecule	Technique	Number of patients analyzed	Type of tissue analyzed	Citation/Link
GSE240623	Targeted mRNA Sequencing	mRNA	HTG-EdgeSeq PIP assay	26	Colon cancer (EOCC = 13; LOCC = 13) and adjacent normal tissue samples (EOCC = 13; LOCC = 13)	This study
TCGA COAD	RNA-Sequencing	mRNA	RNA-Sequencing	454	Colon cancer (EOCC = 53; LOCC = 451) and adjacent normal tissue samples	http://xena.ucsc.edu/
GSE39396	Single-Cell RNA Sequencing	mRNA	Microarray	6	Colon cancer	Calon A. et al; Cancer Cell 2012
GSE39582	Targeted mRNA Sequencing	mRNA	Microarray	508	Colon cancer (EOCC = 54; LOCC = 454) and adjacent normal tissue samples	Marisa L. et al: Plos Med 2013
GSE146771	Single-Cell RNA Sequencing	mRNA	SMART-Seq2	10	Colon cancer	Zhang L. et al; Cell 2020
GSE240531	Nanostring GeoMx DSP	mRNA	Cancer Transcriptome Atlas	8	Colon cancer (EOCC = 4; LOCC = 4) and adjacent normal tissue samples (EOCC = 4; LOCC = 4)	This study

Supplementary Table 2. Clinicopathological features of sporadic EOCC (<50 yr.) and LOCC (≥50 yr.) patients included in the study and the techniques utilized to assess them.

No.	HTG EdgeSeq PIP Sample Name for Tumor	HTG EdgeSeq PIP Sample Name for Normal	GeoMx DSP	mIF	Age at diagnosis	Gender	Race	Location	BMI (kg/m^2)	Family history	IBD	Preoperative Treatment	Grade	pT	pN	pM	pStage	MSI status	KRAS Status	BRAF Status	NRAS Status	TP53 Status
1	EOCC_01_T	EOCC_01_N	EOCC_01	Opal_Y01	28	Female	Caucasian	Right	25.1	No	No	No	G2	pT4a	pN1a	pM0	III	MSS	Mutant	Wild	Wild	NA
2	EOCC_02_T	EOCC_02_N	EOCC_03	Opal_Y03	30	Male	Asian	Right	16.5	No	No	No	G2	pT3	pN1b	pM0	III	MSS	NA	NA	NA	NA
3	EOCC_03_T	EOCC_03_N	EOCC_04	Opal_Y05	33	Female	Caucasian	Left	20.0	No	No	No	G2	pT4b	pN2a	pM0	III	MSS	Mutant	Wild	Wild	NA
4	EOCC_04_T	EOCC_04_N	EOCC_02	Opal_Y02	36	Female	Caucasian	Left	26.5	No	No	No	G2	pT3	pN1b	pM0	III	MSS	NA	NA	NA	NA
5	EOCC_06_T	EOCC_06_N		Opal_Y04	36	Female	Caucasian	Left	25.0	No	No	No	G2	pT3	pN1b	pM0	III	MSS	NA	NA	NA	NA
6	EOCC_07_T	EOCC_07_N			42	Male	Caucasian	Left	22.7	No	No	No	G2	pT3	pN1b	pM0	III	MSS	NA	NA	NA	NA
7	EOCC_08_T	EOCC_08_N			43	Female	Caucasian	Left	18.5	No	No	No	G2	pT3	N0	pM0	II	MSS	Mutant	Wild	Wild	NA
8	EOCC_09_T	EOCC_09_N			43	Male	Asian	Left	20.3	No	No	No	G1	pT1	N2b	M0	III	MSS	N.A.	N.A.	N.A.	NA
9	EOCC_11_T	EOCC_11_N			44	Female	Caucasian	Right	30.3	No	No	No	G2	pT3	N1a	M0	III	MSS	N.A.	N.A.	N.A.	NA
10	EOCC_12_T	EOCC_12_N		Opal_Y06	46	Female	Caucasian	Right	22.1	No	No	No	G3	pT4a	N2b	M0	III	MSS	Wild	Mutant	Wild	NA
11	EOCC_13_T	EOCC_13_N			46	Male	Caucasian	Left	25.1	No	No	No	G1	pT2	N0	M0	I	MSS	NA	NA	NA	NA
12	EOCC_14_T	EOCC_14_N			47	Female	Caucasian	Left	19.6	No	No	No	G2	pT3	N0	M0	II	MSS	Wild	Wild	Wild	NA
13	EOCC_15_T	EOCC_15_N			47	Male	American Indian	Left	23.7	No	No	No	G2	pT3	N2b	pM1	IV	MSS	Mutant	Wild	Wild	NA
14	LOCC_01_T	LOCC_01_N			57	Female	Caucasian	Left	28.9	No	No	No	G2	T2	N0	pM0	I	MSS	NA	NA	NA	NA
15	LOCC_02_T	LOCC_02_N			61	Female	Caucasian	Left	30.0	No	No	No	G2	T3	N0	pM0	II	MSS	NA	NA	NA	NA
16	LOCC_03_T	LOCC_03_N			61	Male	Caucasian	Left	23.7	No	No	No	G3	pT4	N2b	pM0	IV	MSS	Wild	Wild	Wild	NA
17	LOCC_04_T	LOCC_04_N			62	Female	Caucasian	Left	26.7	No	No	No	G2	pT4a	N1c	pM0	III	MSS	NA	NA	NA	NA
18	LOCC_05_T	LOCC_05_N			63	Male	Other	Right	24.5	No	No	No	G2	pT2	N0	pM0	I	MSS	NA	NA	NA	NA
19	LOCC_08_T	LOCC_08_N	LOCC_01	Opal_O05	70	Male	Asian	Left	21.6	No	No	No	G2	pT2	N1a	pM0	III	MSS	NA	NA	NA	NA
20	LOCC_09_T	LOCC_09_N	LOCC_02	Opal_O01	73	Male	Other	Right	26.3	No	No	No	G2	pT3	pN1a	pM0	III	MSS	Mutant	Wild	Wild	NA
21	LOCC_10_T	LOCC_10_N	LOCC_04	Opal_O03	74	Female	Caucasian	Left	20.2	No	No	No	G2	pT4a	pN2a	pM0	III	MSS	NA	NA	NA	NA
22	LOCC_12_T	LOCC_12_N			76	Female	Caucasian	Right	19.7	No	No	No	G2	pT3	N0	pM0	II	MSS	NA	NA	NA	NA
23	LOCC_13_T	LOCC_13_N		Opal_O04	79	Female	Caucasian	Left	24.4	No	No	No	G2	pT3	pN1a	pM0	III	MSS	NA	NA	NA	NA
24	LOCC_15_T	LOCC_15_N	LOCC_03	Opal_O02	82	Female	Caucasian	Left	20.3	No	No	No	G1	pT3	pN1a	pM0	III	MSS	NA	NA	NA	NA
25	LOCC_16_T	LOCC_16_N		Opal_O06	84	Male	Caucasian	Right	26.5	No	No	No	G2	pT3	N1a	pM0	III	MSS	NA	NA	NA	NA
26	LOCC_17_T	LOCC_17_N			89	Male	Caucasian	Right	25.1	No	No	No	G2	pT4a	pN1b	pM1	IV	MSS	Mutant	Wild	Wild	NA

PIP Precision Immuno-Oncology Panel
DSP Digital Spatial Profiler
mIF multiplex immunofluorescence
EOCC early-onset colon cancer
LOCC late-onset colon cancer
BMI Body Mass Index
IBD Inflammatory Bowel Disease
G histological grade
pT pathological Tumor
pN pathological Node
pM pathological Metastasis
MSI Microsatellite instability
MSS Microsatellite stable
NA not available

Supplementary Table 3. Clinicopathological features of sporadic EOCC and LOCC patients in HTG EdgeSeq PIP analysis.

Variables		Age at Diagnosis			Univariate
		EOCC (<50 yr.) n=13	LOCC (≥50 yr.) n=13	Total	*p*-value
Age (y.o.), Median (range)		43 (28-47)	73 (57-89)		< 0.001
Gender					
	Male	5	6	11	1
	Female	8	7	15	
BMI (kg/m^2), Mean, ±SD		22.7 (±3.8)	24.5 (±3.3)		0.224
Race					
	Caucasian	10	10	20	0.593
	Asian	2	1	3	
	American Indian	1	0	1	
	Others	0	2	2	
Location					
	Right	9	8	17	1
	Left	4	5	9	
Histological grade					
	G1	2	1	3	1
	G2	10	11	21	
	G3	1	1	2	
Stage					
	I	1	2	3	0.866
	II	2	2	4	
	III	9	7	16	
	IV	1	2	3	

EOCC early-onset colon cancer
LOCC late-onset colon cancer
PIP Precision Immuno-Oncology Panel
BMI Body Mass Index
SD Standard deviation
G Grade

Supplementary Table 4. Clinicopathological features of sporadic EOCC and LOCC patients in TCGA COAD database.

Variables		Age at Diagnosis			Univariate
		EOCC (<50 yr.) n=53	LOCC (≥50 yr.) n = 401	Total	*p*-value
Age (y.o.), Median (range)		44 (31-49)	71 (50-90)		< 0.001
Gender					
	Male	23	220	243	0.143
	Female	30	181	211	
BMI (kg/m2), Median (range)		26.22 (18.9-38.4)	27.0 (14.7-52.1)		0.436
Race					
	White	29	174	203	0.519
	African American	9	48	57	
	Asian	3	8	11	
	American Indian	0	1	1	
	NA	12	170	182	
Location					
	Right	20	184	204	0.543
	Left	19	124	143	
	NA	14	93	107	
Histological type					
	Tubular	40	342	382	0.081
	Mucinous	13	53	66	
	Others	0	6	6	
CMS					
	1	6	59	65	0.161
	2	12	135	147	
	3	6	47	53	
	4	18	86	104	
	NOLBL or NA	11	74	85	
Stage					
	I	4	73	77	0.077
	II	17	157	174	
	III	19	108	127	
	IV	12	53	65	
	NA	1	10	11	

BMI body mass index
CMS consensus molecular subtype
TCGA The Cancer Genome Atlas
COAD colon adenocarcinoma
EOCC early-onset colon cancer
LOCC late-onset colon cancer
NA not available
NOLBL tumor with no label

Supplementary Table 5. Clinicopathological characteristics of patients assessed for FAP detection in mulitplex immunofluorescence.

Annotation Opal	Age at Diagnosis	Gender	Race	Location	Preoperative Treatment	IBD	pT	pN	pM	Stage	MSI status
Opal_FAP_1	40	Male	Caucasian	Right	No	No	pT2	pN1a	pM0	IIIb	MSS
Opal_FAP_2	42	Male	Caucasian	Left	No	No	pT3	pN1b	pM0	IIIb	MSS
Opal_FAP_3	44	Female	Caucasian	Right	No	No	pT3	pN1a	pM0	IIIb	MSS
Opal_FAP_4	62	Female	Caucasian	Left	No	No	pT4a	pN1b	pM0	IIIc	MSS
Opal_FAP_5	63	Male	Other	Right	No	No	pT2	pN1a	pM0	IIIa	MSS
Opal_FAP_6	68	Male	Asian	Right	No	No	pT3	pN1a	pM0	IIIb	MSS

FAP Fibroblast activation protein
mIF multiplex immunofluorescence
IBD Inframattory bowel disease
pT pathological Tumor
pN pathological Node
pM pathological Metastasis
MSI Microsatellite instability
EOCC early-onset colon cancer
LOCC late-onset colon cancer
MSS Microsatellite stable

Supplementary Table 6. List of antibodies and dilutions utilized for morphological markers in GeoMx DSP, immunohistochemistory, and Western blot assay.

Morphological markers used in GeoMx DSP

Reagents name	Class	Type	Dilution	Cat #	Supplier	Fluorophore Ex. wavelength (nm)
SYTO 13	-	-	1:2000	S7575	ThermoFisher	488
Vimentin (E-5)Ab PE	IgG	Mouse monoclonal	1:300	sc-373717 PE	Santa cruz Biotechnology	532
PanCK Ab (AE-1/AE-3) Alexa Fluor® 647	IgG	Mouse monoclonal	1:200	NBP2-33200A	Novus Biologicals (Centennial, CO, USA)	647
FAP	IgG	Rabbit monoclonal	1:200	ab240989	Abcam	-
DyLight® 594 Conjugation Kit (Fast) Lightning-Link®	-	-	-	ab201801	Abcam	594

*FAP Ab was conjugated with DyLight® 594 Conjugation Kit (Fast) Lightning-Link® according to the manufacturer's protocol.

Immunohistochemistry

Reagents name	Class	Type	Dilution	Catalog no.	Supplier	Fluorophore Ex. wavelength
FAP	IgG	Rabbit monoclonal	1:200	ab240989	Abcam	-
ACTA2	IgG	Rabitt polyclonal	1:2000	ab5694	Abcam	-

Western blot

Reagents name	Class	Type	Dilution	Catalog no.	Supplier	Fluorophore Ex. wavelength
pFGFR2	IgG	Rabbit polyclonal	1:1000	PA5-105880	ThermoScientific	-
FGFR2	IgG	Rabbit polyclonal	1:1000	13042-1-AP	Proteintech	-
pAKT	IgG	Rabbit polyclonal	1:1000	#9271S	Cell Signaling Technology	-
AKT	IgG	Rabbit polyclonal	1:2000	#9272S	Cell Signaling Technology	-
b-actin	IgG	Mouse polyclonal	1:10000	A5441	Sigma-Aldrich	-
secondary mouse	IgG	Goat polyclonal	1:5000	#7076P2	Cell Signaling Technology	-
secondary rabbit	IgG	Goat polyclonal	1:5000	#7074P2	Cell Signaling Technology	-

Ab Antibodies
DSP Digital spatial profiler
PanCK Pan-cytokeratin
FAP Fibroblast activation protein
ACTA2 Actin alpha 2, smooth muscle
Ex Excitaion

Supplementary Table 7. Phenotype proportions obtained by CIBERSORTx using GSE39582 dataset.

Sample	EPCAM	FAP	CD45	CD31
EOCC_001	0.743	0.134	0.112	0.012
EOCC_002	0.494	0.453	0.019	0.034
EOCC_003	0.778	0.177	0.030	0.016
EOCC_004	0.053	0.828	0.003	0.116
EOCC_005	0.455	0.419	0.068	0.058
EOCC_006	0.911	0.003	0.044	0.042
EOCC_007	0.134	0.557	0.167	0.142
EOCC_008	0.905	0.011	0.032	0.052
EOCC_009	0.549	0.350	0.074	0.027
EOCC_010	0.641	0.229	0.073	0.057
EOCC_011	0.945	0.000	0.029	0.026
EOCC_012	0.234	0.660	0.071	0.035
EOCC_013	0.389	0.464	0.101	0.047
EOCC_014	0.634	0.251	0.099	0.017
EOCC_015	0.890	0.057	0.029	0.024
EOCC_016	0.586	0.229	0.162	0.023
EOCC_017	0.862	0.050	0.050	0.038
EOCC_018	0.838	0.150	0.011	0.000
EOCC_019	0.521	0.340	0.068	0.070
EOCC_020	0.747	0.100	0.112	0.041
EOCC_021	0.947	0.052	0.000	0.002
EOCC_022	0.779	0.117	0.066	0.039
EOCC_023	0.530	0.104	0.197	0.169
EOCC_024	0.697	0.136	0.119	0.048
EOCC_025	0.173	0.680	0.084	0.063
EOCC_026	0.558	0.261	0.129	0.052
EOCC_027	0.091	0.701	0.060	0.147
EOCC_028	0.205	0.706	0.041	0.049
EOCC_029	0.973	0.026	0.000	0.001
EOCC_030	0.504	0.353	0.143	0.000
EOCC_031	0.885	0.005	0.060	0.050
EOCC_032	0.765	0.033	0.075	0.127
EOCC_033	0.827	0.043	0.076	0.055
EOCC_034	0.757	0.093	0.115	0.035
EOCC_035	0.310	0.575	0.038	0.078
EOCC_036	0.913	0.043	0.023	0.022
EOCC_037	0.753	0.113	0.082	0.052
EOCC_038	0.958	0.026	0.009	0.006
EOCC_039	0.671	0.267	0.045	0.016
EOCC_040	0.952	0.003	0.028	0.017
EOCC_041	0.607	0.199	0.091	0.103
EOCC_042	0.611	0.224	0.133	0.031
EOCC_043	0.685	0.120	0.096	0.099
EOCC_044	0.255	0.497	0.171	0.077
EOCC_045	0.858	0.064	0.044	0.033
EOCC_046	0.878	0.057	0.039	0.026
EOCC_047	0.788	0.053	0.075	0.084
EOCC_048	0.801	0.088	0.069	0.042
EOCC_049	0.845	0.091	0.034	0.030
EOCC_050	0.808	0.050	0.074	0.068
EOCC_051	0.755	0.245	0.000	0.000
EOCC_052	0.735	0.087	0.114	0.064
EOCC_053	0.558	0.218	0.164	0.060
EOCC_054	0.641	0.273	0.073	0.014
LOCC_001	0.734	0.128	0.057	0.082
LOCC_002	0.513	0.399	0.031	0.057

LOCC_003	0.886	0.089	0.015	0.010
LOCC_004	0.380	0.493	0.054	0.073
LOCC_005	0.443	0.422	0.052	0.084
LOCC_006	0.780	0.089	0.080	0.052
LOCC_007	0.554	0.319	0.127	0.000
LOCC_008	0.465	0.344	0.185	0.006
LOCC_009	0.820	0.094	0.027	0.059
LOCC_010	0.840	0.049	0.057	0.055
LOCC_011	0.597	0.171	0.165	0.067
LOCC_012	0.692	0.175	0.099	0.034
LOCC_013	0.718	0.229	0.053	0.000
LOCC_014	0.900	0.064	0.029	0.007
LOCC_015	0.868	0.000	0.072	0.060
LOCC_016	0.547	0.417	0.022	0.014
LOCC_017	0.742	0.103	0.081	0.074
LOCC_018	0.668	0.127	0.116	0.088
LOCC_019	0.788	0.102	0.043	0.067
LOCC_020	0.654	0.333	0.000	0.014
LOCC_021	0.926	0.007	0.044	0.023
LOCC_022	0.812	0.066	0.050	0.072
LOCC_023	0.299	0.635	0.033	0.034
LOCC_024	0.778	0.178	0.011	0.032
LOCC_025	0.679	0.114	0.115	0.092
LOCC_026	0.448	0.265	0.237	0.050
LOCC_027	0.543	0.297	0.161	0.000
LOCC_028	0.112	0.435	0.323	0.130
LOCC_029	0.678	0.222	0.077	0.023
LOCC_030	0.648	0.231	0.080	0.041
LOCC_031	0.874	0.070	0.045	0.010
LOCC_032	0.365	0.311	0.251	0.073
LOCC_033	0.934	0.000	0.045	0.021
LOCC_034	0.228	0.610	0.064	0.098
LOCC_035	0.441	0.509	0.030	0.021
LOCC_036	0.375	0.489	0.072	0.064
LOCC_037	0.518	0.373	0.054	0.055
LOCC_038	0.708	0.077	0.186	0.029
LOCC_039	0.000	0.729	0.015	0.256
LOCC_040	0.964	0.000	0.020	0.016
LOCC_041	0.721	0.233	0.011	0.034
LOCC_042	0.382	0.495	0.029	0.094
LOCC_043	0.775	0.133	0.067	0.025
LOCC_044	0.702	0.286	0.008	0.004
LOCC_045	0.825	0.136	0.015	0.025
LOCC_046	0.294	0.365	0.052	0.289
LOCC_047	0.680	0.169	0.083	0.067
LOCC_048	0.858	0.142	0.000	0.000
LOCC_049	0.712	0.144	0.068	0.075
LOCC_050	0.775	0.063	0.115	0.047
LOCC_051	0.474	0.038	0.292	0.196
LOCC_052	0.770	0.097	0.072	0.061
LOCC_053	0.793	0.163	0.024	0.021
LOCC_054	0.549	0.276	0.087	0.087
LOCC_055	0.730	0.159	0.045	0.065
LOCC_056	0.792	0.066	0.082	0.060
LOCC_057	0.062	0.652	0.227	0.059
LOCC_058	0.213	0.731	0.033	0.022
LOCC_059	0.798	0.092	0.083	0.028
LOCC_060	0.802	0.182	0.000	0.015

LOCC_061	0.924	0.053	0.023	0.000
LOCC_062	0.701	0.203	0.095	0.000
LOCC_063	0.252	0.624	0.050	0.074
LOCC_064	0.793	0.128	0.028	0.052
LOCC_065	0.881	0.111	0.007	0.002
LOCC_066	0.847	0.066	0.046	0.041
LOCC_067	0.018	0.878	0.061	0.043
LOCC_068	0.625	0.287	0.046	0.042
LOCC_069	0.922	0.047	0.012	0.018
LOCC_070	0.532	0.343	0.061	0.064
LOCC_071	0.562	0.269	0.113	0.056
LOCC_072	0.861	0.102	0.013	0.023
LOCC_073	0.866	0.113	0.006	0.015
LOCC_074	0.716	0.202	0.058	0.024
LOCC_075	0.823	0.156	0.010	0.011
LOCC_076	0.695	0.127	0.117	0.061
LOCC_077	0.404	0.496	0.069	0.031
LOCC_078	0.615	0.246	0.064	0.075
LOCC_079	0.669	0.174	0.082	0.074
LOCC_080	0.735	0.255	0.000	0.010
LOCC_081	0.641	0.184	0.159	0.016
LOCC_082	0.395	0.426	0.175	0.004
LOCC_083	0.506	0.380	0.057	0.058
LOCC_084	0.771	0.123	0.068	0.038
LOCC_085	0.984	0.000	0.016	0.000
LOCC_086	0.970	0.000	0.015	0.014
LOCC_087	0.713	0.112	0.080	0.096
LOCC_088	0.830	0.049	0.056	0.065
LOCC_089	0.909	0.073	0.010	0.008
LOCC_090	0.691	0.196	0.082	0.030
LOCC_091	0.840	0.132	0.006	0.021
LOCC_092	0.678	0.149	0.074	0.098
LOCC_093	0.963	0.000	0.020	0.017
LOCC_094	0.746	0.157	0.088	0.009
LOCC_095	0.770	0.198	0.032	0.000
LOCC_096	0.462	0.496	0.031	0.010
LOCC_097	0.560	0.177	0.146	0.117
LOCC_098	0.844	0.049	0.057	0.050
LOCC_099	0.845	0.148	0.008	0.000
LOCC_100	0.486	0.000	0.191	0.324
LOCC_101	0.585	0.297	0.118	0.000
LOCC_102	0.928	0.020	0.033	0.019
LOCC_103	0.473	0.443	0.041	0.044
LOCC_104	0.896	0.030	0.057	0.017
LOCC_105	0.869	0.071	0.037	0.023
LOCC_106	0.941	0.000	0.040	0.019
LOCC_107	0.750	0.144	0.047	0.059
LOCC_108	0.185	0.567	0.130	0.118
LOCC_109	0.531	0.415	0.015	0.039
LOCC_110	0.976	0.024	0.000	0.000
LOCC_111	0.831	0.051	0.072	0.046
LOCC_112	0.282	0.596	0.069	0.053
LOCC_113	0.842	0.080	0.031	0.047
LOCC_114	0.897	0.039	0.039	0.026
LOCC_115	0.758	0.095	0.105	0.041
LOCC_116	0.175	0.469	0.263	0.093
LOCC_117	0.792	0.125	0.059	0.024
LOCC_118	0.858	0.065	0.038	0.040

LOCC_119	0.757	0.190	0.052	0.000
LOCC_120	0.963	0.019	0.014	0.004
LOCC_121	0.834	0.166	0.000	0.000
LOCC_122	0.690	0.273	0.011	0.026
LOCC_123	0.748	0.183	0.067	0.002
LOCC_124	0.906	0.019	0.041	0.035
LOCC_125	0.295	0.582	0.087	0.037
LOCC_126	0.380	0.141	0.262	0.216
LOCC_127	0.280	0.462	0.198	0.060
LOCC_128	0.853	0.098	0.020	0.030
LOCC_129	0.550	0.293	0.084	0.073
LOCC_130	0.476	0.303	0.092	0.129
LOCC_131	0.658	0.237	0.072	0.033
LOCC_132	0.655	0.313	0.030	0.001
LOCC_133	0.882	0.060	0.025	0.033
LOCC_134	0.710	0.096	0.107	0.086
LOCC_135	0.875	0.089	0.010	0.027
LOCC_136	0.000	0.721	0.179	0.099
LOCC_137	0.762	0.072	0.092	0.074
LOCC_138	0.668	0.289	0.009	0.033
LOCC_139	0.872	0.008	0.071	0.049
LOCC_140	0.923	0.009	0.046	0.022
LOCC_141	0.927	0.060	0.000	0.012
LOCC_142	0.000	0.569	0.177	0.254
LOCC_143	0.585	0.227	0.152	0.036
LOCC_144	0.616	0.233	0.103	0.048
LOCC_145	0.729	0.234	0.011	0.025
LOCC_146	0.811	0.092	0.064	0.033
LOCC_147	0.860	0.012	0.074	0.054
LOCC_148	0.671	0.191	0.039	0.099
LOCC_149	0.888	0.024	0.051	0.037
LOCC_150	0.955	0.000	0.036	0.009
LOCC_151	0.947	0.000	0.034	0.019
LOCC_152	0.615	0.248	0.064	0.073
LOCC_153	0.713	0.142	0.070	0.075
LOCC_154	0.949	0.031	0.012	0.009
LOCC_155	0.429	0.384	0.127	0.060
LOCC_156	0.712	0.181	0.091	0.017
LOCC_157	0.144	0.240	0.502	0.114
LOCC_158	0.562	0.091	0.166	0.180
LOCC_159	0.664	0.097	0.152	0.086
LOCC_160	0.678	0.245	0.036	0.041
LOCC_161	0.782	0.201	0.006	0.011
LOCC_162	0.776	0.139	0.047	0.038
LOCC_163	0.851	0.136	0.012	0.000
LOCC_164	0.195	0.652	0.134	0.019
LOCC_165	0.588	0.412	0.000	0.000
LOCC_166	0.969	0.018	0.009	0.005
LOCC_167	0.321	0.426	0.131	0.122
LOCC_168	0.948	0.000	0.026	0.026
LOCC_169	0.528	0.217	0.214	0.041
LOCC_170	0.977	0.000	0.014	0.009
LOCC_171	0.438	0.365	0.159	0.038
LOCC_172	0.849	0.084	0.043	0.024
LOCC_173	0.867	0.062	0.052	0.019
LOCC_174	0.645	0.269	0.086	0.000
LOCC_175	0.501	0.425	0.042	0.032
LOCC_176	0.586	0.073	0.189	0.152

LOCC_177	0.265	0.607	0.108	0.021
LOCC_178	0.911	0.009	0.051	0.029
LOCC_179	0.878	0.073	0.023	0.026
LOCC_180	0.818	0.093	0.043	0.046
LOCC_181	0.951	0.000	0.029	0.021
LOCC_182	0.795	0.095	0.068	0.041
LOCC_183	0.382	0.600	0.007	0.011
LOCC_184	0.599	0.292	0.035	0.074
LOCC_185	0.981	0.000	0.011	0.008
LOCC_186	0.940	0.000	0.030	0.030
LOCC_187	0.000	0.711	0.185	0.104
LOCC_188	0.821	0.095	0.052	0.031
LOCC_189	0.628	0.315	0.039	0.019
LOCC_190	0.855	0.024	0.049	0.072
LOCC_191	0.633	0.182	0.146	0.039
LOCC_192	0.276	0.550	0.018	0.157
LOCC_193	0.708	0.193	0.058	0.042
LOCC_194	0.598	0.325	0.077	0.000
LOCC_195	0.649	0.292	0.016	0.043
LOCC_196	0.800	0.071	0.053	0.076
LOCC_197	0.503	0.316	0.092	0.089
LOCC_198	0.683	0.112	0.097	0.109
LOCC_199	0.735	0.242	0.014	0.010
LOCC_200	0.911	0.028	0.029	0.032
LOCC_201	0.888	0.096	0.011	0.004
LOCC_202	0.574	0.202	0.084	0.140
LOCC_203	0.677	0.240	0.056	0.027
LOCC_204	0.765	0.130	0.061	0.045
LOCC_205	0.475	0.462	0.047	0.016
LOCC_206	0.897	0.007	0.069	0.027
LOCC_207	0.573	0.407	0.012	0.007
LOCC_208	0.747	0.150	0.086	0.017
LOCC_209	0.629	0.228	0.103	0.040
LOCC_210	0.491	0.261	0.116	0.133
LOCC_211	0.748	0.119	0.045	0.087
LOCC_212	0.972	0.000	0.019	0.010
LOCC_213	0.000	0.801	0.000	0.199
LOCC_214	0.708	0.124	0.118	0.050
LOCC_215	0.365	0.481	0.113	0.042
LOCC_216	0.637	0.321	0.032	0.010
LOCC_217	0.909	0.024	0.044	0.024
LOCC_218	0.335	0.360	0.142	0.163
LOCC_219	0.927	0.046	0.008	0.019
LOCC_220	0.614	0.290	0.034	0.061
LOCC_221	0.266	0.373	0.192	0.169
LOCC_222	0.300	0.449	0.115	0.135
LOCC_223	0.839	0.134	0.018	0.009
LOCC_224	0.947	0.049	0.002	0.002
LOCC_225	0.802	0.092	0.038	0.068
LOCC_226	0.859	0.041	0.059	0.040
LOCC_227	0.944	0.005	0.034	0.017
LOCC_228	0.894	0.032	0.034	0.040
LOCC_229	0.949	0.000	0.037	0.014
LOCC_230	0.965	0.000	0.021	0.014
LOCC_231	0.721	0.184	0.034	0.061
LOCC_232	0.700	0.153	0.059	0.087
LOCC_233	0.024	0.559	0.320	0.096
LOCC_234	0.607	0.129	0.157	0.107

LOCC_235	0.930	0.006	0.041	0.023
LOCC_236	0.328	0.578	0.039	0.055
LOCC_237	0.346	0.336	0.260	0.058
LOCC_238	0.237	0.608	0.061	0.094
LOCC_239	0.805	0.160	0.009	0.026
LOCC_240	0.901	0.011	0.052	0.037
LOCC_241	0.917	0.082	0.000	0.001
LOCC_242	0.772	0.172	0.040	0.016
LOCC_243	0.744	0.082	0.061	0.113
LOCC_244	0.918	0.005	0.038	0.039
LOCC_245	0.210	0.398	0.283	0.109
LOCC_246	0.608	0.222	0.124	0.047
LOCC_247	0.579	0.241	0.096	0.083
LOCC_248	0.869	0.121	0.000	0.010
LOCC_249	0.959	0.000	0.014	0.027
LOCC_250	0.684	0.192	0.065	0.060
LOCC_251	0.423	0.373	0.086	0.118
LOCC_252	0.916	0.065	0.019	0.000
LOCC_253	0.832	0.112	0.029	0.027
LOCC_254	0.794	0.088	0.056	0.062
LOCC_255	0.888	0.035	0.048	0.029
LOCC_256	0.834	0.105	0.027	0.034
LOCC_257	0.032	0.650	0.153	0.165
LOCC_258	0.780	0.196	0.009	0.015
LOCC_259	0.942	0.047	0.010	0.001
LOCC_260	0.757	0.106	0.031	0.105
LOCC_261	0.890	0.080	0.011	0.019
LOCC_262	0.956	0.000	0.025	0.020
LOCC_263	0.934	0.016	0.021	0.028
LOCC_264	0.741	0.172	0.049	0.039
LOCC_265	0.599	0.235	0.077	0.090
LOCC_266	0.759	0.229	0.010	0.002
LOCC_267	0.653	0.178	0.046	0.123
LOCC_268	0.977	0.000	0.011	0.012
LOCC_269	0.951	0.047	0.003	0.000
LOCC_270	0.534	0.377	0.083	0.007
LOCC_271	0.937	0.002	0.035	0.026
LOCC_272	0.534	0.117	0.285	0.063
LOCC_273	0.961	0.039	0.000	0.000
LOCC_274	0.261	0.433	0.152	0.153
LOCC_275	0.718	0.147	0.083	0.052
LOCC_276	0.790	0.048	0.109	0.053
LOCC_277	0.688	0.219	0.056	0.037
LOCC_278	0.661	0.288	0.026	0.025
LOCC_279	0.727	0.223	0.050	0.000
LOCC_280	0.000	0.546	0.347	0.106
LOCC_281	0.164	0.822	0.005	0.009
LOCC_282	0.977	0.020	0.002	0.001
LOCC_283	0.417	0.373	0.175	0.035
LOCC_284	0.458	0.369	0.119	0.053
LOCC_285	0.559	0.359	0.033	0.048
LOCC_286	0.912	0.009	0.036	0.043
LOCC_287	0.943	0.000	0.033	0.024
LOCC_288	0.802	0.134	0.060	0.004
LOCC_289	0.807	0.090	0.069	0.034
LOCC_290	0.803	0.058	0.091	0.048
LOCC_291	0.804	0.133	0.026	0.037
LOCC_292	0.662	0.268	0.054	0.016

LOCC_293	0.731	0.227	0.029	0.014
LOCC_294	0.876	0.090	0.015	0.020
LOCC_295	0.573	0.226	0.133	0.067
LOCC_296	0.615	0.193	0.072	0.121
LOCC_297	0.873	0.037	0.041	0.049
LOCC_298	0.721	0.190	0.063	0.026
LOCC_299	0.786	0.091	0.075	0.048
LOCC_300	0.724	0.185	0.054	0.037
LOCC_301	0.448	0.549	0.000	0.002
LOCC_302	0.694	0.218	0.088	0.000
LOCC_303	0.829	0.120	0.033	0.018
LOCC_304	0.551	0.384	0.026	0.039
LOCC_305	0.801	0.062	0.069	0.067
LOCC_306	0.637	0.162	0.105	0.096
LOCC_307	0.713	0.210	0.075	0.003
LOCC_308	0.624	0.222	0.105	0.049
LOCC_309	0.712	0.106	0.099	0.082
LOCC_310	0.531	0.361	0.022	0.087
LOCC_311	0.175	0.616	0.130	0.079
LOCC_312	0.951	0.000	0.032	0.017
LOCC_313	0.681	0.293	0.012	0.014
LOCC_314	0.027	0.771	0.041	0.161
LOCC_315	0.687	0.252	0.034	0.026
LOCC_316	0.850	0.021	0.062	0.067
LOCC_317	0.015	0.732	0.168	0.086
LOCC_318	0.967	0.000	0.017	0.016
LOCC_319	0.537	0.290	0.146	0.027
LOCC_320	0.478	0.344	0.093	0.084
LOCC_321	0.943	0.043	0.006	0.007
LOCC_322	0.895	0.086	0.013	0.006
LOCC_323	0.902	0.015	0.071	0.013
LOCC_324	0.882	0.041	0.058	0.020
LOCC_325	0.822	0.111	0.036	0.031
LOCC_326	0.885	0.115	0.000	0.000
LOCC_327	0.759	0.138	0.062	0.041
LOCC_328	0.303	0.446	0.251	0.000
LOCC_329	0.944	0.000	0.028	0.028
LOCC_330	0.628	0.291	0.038	0.043
LOCC_331	0.000	0.751	0.140	0.109
LOCC_332	0.752	0.111	0.083	0.054
LOCC_333	0.000	0.632	0.221	0.147
LOCC_334	0.617	0.316	0.026	0.042
LOCC_335	0.579	0.261	0.105	0.056
LOCC_336	0.909	0.001	0.048	0.042
LOCC_337	0.668	0.160	0.139	0.033
LOCC_338	0.924	0.075	0.001	0.000
LOCC_339	0.726	0.224	0.032	0.018
LOCC_340	0.666	0.103	0.097	0.134
LOCC_341	0.576	0.208	0.122	0.094
LOCC_342	0.481	0.320	0.183	0.016
LOCC_343	0.184	0.288	0.272	0.256
LOCC_344	0.776	0.051	0.102	0.071
LOCC_345	0.981	0.000	0.008	0.011
LOCC_346	0.660	0.300	0.000	0.039
LOCC_347	0.979	0.002	0.007	0.012
LOCC_348	0.924	0.014	0.033	0.028
LOCC_349	0.550	0.281	0.123	0.046
LOCC_350	0.752	0.132	0.053	0.063

LOCC_351	0.331	0.625	0.000	0.044
LOCC_352	0.575	0.353	0.012	0.060
LOCC_353	0.625	0.274	0.101	0.000
LOCC_354	0.798	0.064	0.055	0.082
LOCC_355	0.846	0.053	0.067	0.035
LOCC_356	0.813	0.076	0.095	0.017
LOCC_357	0.482	0.423	0.031	0.064
LOCC_358	0.906	0.000	0.077	0.017
LOCC_359	0.781	0.085	0.083	0.051
LOCC_360	0.705	0.233	0.025	0.036
LOCC_361	0.968	0.000	0.016	0.015
LOCC_362	0.777	0.218	0.005	0.000
LOCC_363	0.796	0.131	0.037	0.035
LOCC_364	0.779	0.102	0.051	0.068
LOCC_365	0.868	0.103	0.013	0.017
LOCC_366	0.842	0.021	0.086	0.050
LOCC_367	0.370	0.357	0.138	0.135
LOCC_368	0.914	0.051	0.015	0.020
LOCC_369	0.780	0.084	0.093	0.043
LOCC_370	0.322	0.510	0.135	0.032
LOCC_371	0.463	0.229	0.130	0.178
LOCC_372	0.998	0.000	0.002	0.000
LOCC_373	0.699	0.228	0.044	0.029
LOCC_374	0.648	0.262	0.065	0.025
LOCC_375	0.685	0.199	0.091	0.024
LOCC_376	0.971	0.000	0.016	0.013
LOCC_377	0.978	0.000	0.007	0.015
LOCC_378	0.942	0.051	0.000	0.008
LOCC_379	0.939	0.000	0.025	0.036
LOCC_380	0.831	0.151	0.019	0.000
LOCC_381	0.568	0.322	0.074	0.035
LOCC_382	0.908	0.073	0.014	0.006
LOCC_383	0.924	0.021	0.029	0.027
LOCC_384	0.635	0.112	0.140	0.113
LOCC_385	0.657	0.264	0.038	0.041
LOCC_386	0.300	0.577	0.044	0.079
LOCC_387	0.000	0.882	0.118	0.000
LOCC_388	0.701	0.130	0.105	0.065
LOCC_389	0.571	0.268	0.119	0.042
LOCC_390	0.885	0.103	0.009	0.003
LOCC_391	0.690	0.219	0.065	0.026
LOCC_392	0.568	0.041	0.227	0.163
LOCC_393	0.885	0.020	0.043	0.052
LOCC_394	0.854	0.087	0.040	0.019
LOCC_395	0.486	0.484	0.023	0.007
LOCC_396	0.551	0.320	0.089	0.040
LOCC_397	0.597	0.318	0.055	0.030
LOCC_398	0.557	0.411	0.013	0.018
LOCC_399	0.802	0.025	0.072	0.101
LOCC_400	0.839	0.041	0.059	0.060
LOCC_401	0.339	0.650	0.007	0.004
LOCC_402	0.652	0.283	0.049	0.017
LOCC_403	0.842	0.150	0.002	0.006
LOCC_404	0.520	0.408	0.040	0.032
LOCC_405	0.650	0.137	0.094	0.119
LOCC_406	0.588	0.329	0.061	0.022
LOCC_407	0.911	0.089	0.000	0.000
LOCC_408	0.484	0.314	0.146	0.057

LOCC_409	0.938	0.000	0.031	0.030
LOCC_410	0.432	0.398	0.133	0.037
LOCC_411	0.702	0.136	0.080	0.082
LOCC_412	0.809	0.153	0.038	0.000
LOCC_413	0.663	0.337	0.000	0.000
LOCC_414	0.768	0.044	0.108	0.080
LOCC_415	0.568	0.200	0.152	0.080
LOCC_416	0.855	0.026	0.059	0.061
LOCC_417	0.784	0.000	0.128	0.088
LOCC_418	0.973	0.001	0.019	0.007
LOCC_419	0.601	0.349	0.016	0.034
LOCC_420	0.947	0.004	0.024	0.025
LOCC_421	0.955	0.003	0.021	0.021
LOCC_422	0.927	0.009	0.044	0.020
LOCC_423	0.390	0.322	0.165	0.124
LOCC_424	0.587	0.336	0.078	0.000
LOCC_425	0.174	0.445	0.176	0.206
LOCC_426	0.486	0.440	0.017	0.057
LOCC_427	0.816	0.013	0.084	0.087
LOCC_428	0.798	0.021	0.081	0.100
LOCC_429	0.656	0.176	0.079	0.090
LOCC_430	0.949	0.029	0.008	0.014
LOCC_431	0.487	0.348	0.096	0.068
LOCC_432	0.847	0.013	0.080	0.060
LOCC_433	0.749	0.059	0.145	0.048
LOCC_434	0.914	0.016	0.027	0.042
LOCC_435	0.977	0.000	0.011	0.011
LOCC_436	0.661	0.309	0.019	0.010
LOCC_437	0.681	0.087	0.122	0.110
LOCC_438	0.811	0.185	0.004	0.000
LOCC_439	0.969	0.000	0.017	0.015
LOCC_440	0.731	0.238	0.004	0.026
LOCC_441	0.498	0.380	0.061	0.061
LOCC_442	0.526	0.380	0.094	0.000
LOCC_443	0.814	0.165	0.013	0.009
LOCC_444	0.652	0.327	0.017	0.003
LOCC_445	0.720	0.240	0.023	0.017
LOCC_446	0.508	0.428	0.023	0.041
LOCC_447	0.936	0.039	0.005	0.021
LOCC_448	0.644	0.340	0.000	0.017
LOCC_449	0.855	0.101	0.028	0.016
LOCC_450	0.664	0.306	0.023	0.008
LOCC_451	0.564	0.235	0.144	0.058
LOCC_452	0.902	0.017	0.042	0.040
LOCC_453	0.901	0.083	0.000	0.016
LOCC_454	0.598	0.286	0.116	0.000
EOCC average	0.655	0.223	0.073	0.049
LOCC average	0.681	0.205	0.067	0.047
EOCC vs LOCC *P* value (One way ANOVA)	NS	NS	NS	NS

EOCC early-onset colon cancer
LOCC late-onset colon cancer
ANOVA analysis of variance
NS no significance

Supplementary Table 8. Upregulated cancer-related pathways in EPCAM+ tumor epithelial cells of EOCC patients.

NAME	NES	FDR
WP_COMPLEMENT_SYSTEM	2.071	0.004
WP_WNT_SIGNALING_PATHWAY	2.054	0.003
WP_DNA_REPLICATION	1.909	0.009
WP_VEGFAVEGFR2_SIGNALING_PATHWAY	1.887	0.011
WP_METABOLIC_REPROGRAMMING_IN_COLON_CANCER	1.874	0.010
WP_LNCRNA_IN_CANONICAL_WNT_SIGNALING_AND_COLORECTAL_CANCER	1.868	0.010
WP_TGFBETA_RECEPTOR_SIGNALING	1.829	0.014
WP_HIPPOYAP_SIGNALING_PATHWAY	1.810	0.016
WP_IL3_SIGNALING_PATHWAY	1.807	0.016
WP_TOLLLIKE_RECEPTOR_SIGNALING_RELATED_TO_MYD88	1.802	0.015
WP_DNA_MISMATCH_REPAIR	1.778	0.017
WP_OVERVIEW_OF_NANOPARTICLE_EFFECTS	1.774	0.017
WP_REGULATION_OF_WNT_BCATENIN_SIGNALING_BY_SMALL_MOLECULE_COMPOUNDS	1.772	0.017
WP_TGFBETA_SIGNALING_PATHWAY	1.768	0.017
WP_TLR4_SIGNALING_AND_TOLERANCE	1.758	0.019
WP_G1_TO_S_CELL_CYCLE_CONTROL	1.695	0.031
WP_CHEMOKINE_SIGNALING_PATHWAY	1.676	0.038
WP_IL7_SIGNALING_PATHWAY	1.676	0.037
WP_CHROMOSOMAL_AND_MICROSATELLITE_INSTABILITY_IN_COLORECTAL_CANCER	1.661	0.038
WP_MECHANOREGULATION_AND_PATHOLOGY_OF_YAPTAZ_VIA_HIPPO_AND_NONHIPPO_MECHANISMS	1.646	0.044
WP_IL17_SIGNALING_PATHWAY	1.624	0.047
WP_IL4_SIGNALING_PATHWAY	1.610	0.052
WP_AMPACTIVATED_PROTEIN_KINASE_AMPK_SIGNALING	1.580	0.063
WP_ERBB_SIGNALING_PATHWAY	1.574	0.065
WP_IL5_SIGNALING_PATHWAY	1.548	0.073
WP_INTERACTIONS_BETWEEN_IMMUNE_CELLS_AND_MICRORNAS_IN_TUMOR_MICROENVIRONMENT	1.539	0.076
WP_IL1_SIGNALING_PATHWAY	1.539	0.076
WP_INTERFERON_TYPE_I_SIGNALING_PATHWAYS	1.529	0.079
WP_SELENIUM_MICRONUTRIENT_NETWORK	1.507	0.091
WP_INTERLEUKIN11_SIGNALING_PATHWAY	1.500	0.094
WP_DNA_DAMAGE_RESPONSE_ONLY_ATM_DEPENDENT	1.500	0.093
WP_AGERAGE_PATHWAY	1.493	0.097

WP_DNA_DAMAGE_RESPONSE	1.487	0.100
WP_REGULATORY_CIRCUITS_OF_THE_STAT3_SIGNALING_PATHWAY	1.483	0.103
WP_CELL_CYCLE	1.472	0.108
WP_MODULATORS_OF_TCR_SIGNALING_AND_T_CELL_ACTIVATION	1.469	0.110
WP_IL18_SIGNALING_PATHWAY	1.466	0.109
WP_IL2_SIGNALING_PATHWAY	1.463	0.108
WP_TNFALPHA_SIGNALING_PATHWAY	1.456	0.110
WP_TCELL_RECEPTOR_TCR_SIGNALING_PATHWAY	1.446	0.114
WP_RAS_SIGNALING	1.431	0.125
WP_MIRNA_REGULATION_OF_PROSTATE_CANCER_SIGNALING_PATHWAYS	1.430	0.126
WP_TOLLLIKE_RECEPTOR_SIGNALING_PATHWAY	1.406	0.141
WP_HAIR_FOLLICLE_DEVELOPMENT_ORGANOGENESIS_PART_2_OF_3	1.405	0.142
WP_REGULATION_OF_ACTIN_CYTOSKELETON	1.398	0.148
WP_CILIARY_LANDSCAPE	1.395	0.150
WP_MALIGNANT_PLEURAL_MESOTHELIOMA	1.395	0.150
WP_CARDIAC_HYPERTROPHIC_RESPONSE	1.394	0.150
WP_SIGNAL_TRANSDUCTION_THROUGH_IL1R	1.394	0.150
WP_MITOCHONDRIAL_COMPLEX_IV_ASSEMBLY	1.392	0.151
WP_22Q112_COPY_NUMBER_VARIATION_SYNDROME	1.392	0.150
WP_OXIDATIVE_DAMAGE_RESPONSE	1.390	0.151
WP_EPITHELIAL_TO_MESENCHYMAL_TRANSITION_IN_COLORECTAL_CANCER	1.381	0.156
WP_TCELL_ANTIGEN_RECEPTOR_TCR_PATHWAY_DURING_STAPHYLOCOCCUS_AUREUS_INFECTION	1.368	0.168
WP_CANONICAL_AND_NONCANONICAL_TGFB_SIGNALING	1.365	0.170
WP_IL9_SIGNALING_PATHWAY	1.345	0.186
WP_FOCAL_ADHESION	1.315	0.212
WP_COMPLEMENT_ACTIVATION	1.313	0.211
WP_EGFR_TYROSINE_KINASE_INHIBITOR_RESISTANCE	1.309	0.214
WP_S1P_RECEPTOR_SIGNAL_TRANSDUCTION	1.302	0.219
WP_NOTCH_SIGNALING	1.300	0.222
WP_ALPHA_6_BETA_4_SIGNALING_PATHWAY	1.287	0.227

WP wikipathway
NES normalized enrichment score
FDR false discovery rate

Supplementary Table 9. Upregulated cancer-related pathways in FAP+ CAFs of EOCC patients

NAME	NES	FDR
WP_WNT_SIGNALING	1.639	0.111
WP_VEGFAVEGFR2_SIGNALING_PATHWAY	1.516	0.211
WP_NOTCH_SIGNALING_PATHWAY	1.470	0.214
WP_REGULATORY_CIRCUITS_OF_THE_STAT3_SIGNALING_PATHWAY	1.402	0.276
WP_INFLAMMATORY_RESPONSE_PATHWAY	1.339	0.369
WP_EPITHELIAL_TO_MESENCHYMAL_TRANSITION_IN_COLORECTAL_CANCER	1.313	0.388
WP_TGFBETA_RECEPTOR_SIGNALING	1.312	0.381
WP_FOCAL_ADHESION	1.278	0.404
WP_CHEMOKINE_SIGNALING_PATHWAY	1.276	0.399
WP_P53_TRANSCRIPTIONAL_GENE_NETWORK	1.168	0.599
WP_PPAR_SIGNALING_PATHWAY	1.122	0.650
WP_CELL_CYCLE	1.121	0.641
WP_TCELL_RECEPTOR_TCR_SIGNALING_PATHWAY	1.110	0.641
WP_TGFBETA_SIGNALING_PATHWAY	1.088	0.649
WP_COMPLEMENT_AND_COAGULATION_CASCADES	1.082	0.654
WP_TOLLLIKE_RECEPTOR_SIGNALING_PATHWAY	1.077	0.658
WP_EGFR_TYROSINE_KINASE_INHIBITOR_RESISTANCE	1.058	0.668
WP_CCL18_SIGNALING_PATHWAY	1.026	0.730
WP_REGULATION_OF_ACTIN_CYTOSKELETON	0.963	0.764
WP_IL18_SIGNALING_PATHWAY	0.959	0.764
WP_FOCAL_ADHESION_PI3KAKTMTORSIGNALING_PATHWAY	0.919	0.797
WP_AMPACTIVATED_PROTEIN_KINASE_AMPK_SIGNALING	0.852	0.830
WP_IL4_SIGNALING_PATHWAY	0.816	0.850
WP_RAS_SIGNALING	0.812	0.850
WP_G_PROTEIN_SIGNALING_PATHWAYS	0.809	0.848
WP_PI3KAKT_SIGNALING_PATHWAY	0.799	0.857
WP_DNA_REPAIR_PATHWAYS_FULL_NETWORK	0.747	0.905
WP_MIRNA_REGULATION_OF_DNA_DAMAGE_RESPONSE	0.679	0.965

WP wikipathway
NES normalized enrichment score
FDR false discovery rate

Supplementary Table 10. Phenotype proportions obtained by CIBERSORTx using TCGA COAD dataset.

Sample	Malignant cells	Fibroblasts	B cells	CD8 T cells	Epithelial cells	Myeloid cells	CD4 T cells	Innate lymphoid cells
EOCC_001	0.7785	0.1211	0.0958	0.0041	0.0000	0.0000	0.0000	0.0006
EOCC_002	0.8828	0.0539	0.0557	0.0077	0.0000	0.0000	0.0000	0.0000
EOCC_003	0.8054	0.0821	0.0928	0.0198	0.0000	0.0000	0.0000	0.0000
EOCC_004	0.8707	0.0915	0.0008	0.0015	0.0320	0.0035	0.0000	0.0000
EOCC_005	0.6520	0.2401	0.0913	0.0053	0.0000	0.0112	0.0000	0.0000
EOCC_006	0.9662	0.0091	0.0179	0.0011	0.0000	0.0000	0.0053	0.0003
EOCC_007	0.9052	0.0474	0.0393	0.0077	0.0000	0.0000	0.0004	0.0000
EOCC_008	0.6605	0.1835	0.1113	0.0296	0.0000	0.0151	0.0000	0.0000
EOCC_009	0.2749	0.3953	0.2163	0.0338	0.0000	0.0797	0.0000	0.0000
EOCC_010	0.9778	0.0200	0.0012	0.0011	0.0000	0.0000	0.0000	0.0000
EOCC_011	0.3934	0.2786	0.2921	0.0358	0.0000	0.0000	0.0000	0.0000
EOCC_012	0.7592	0.1855	0.0553	0.0000	0.0000	0.0000	0.0000	0.0000
EOCC_013	0.9042	0.0257	0.0619	0.0082	0.0000	0.0000	0.0000	0.0000
EOCC_014	0.8258	0.0817	0.0685	0.0179	0.0000	0.0000	0.0062	0.0000
EOCC_015	0.8695	0.1023	0.0251	0.0031	0.0000	0.0000	0.0000	0.0000
EOCC_016	0.3148	0.3413	0.1871	0.0383	0.0000	0.0937	0.0248	0.0000
EOCC_017	0.7989	0.1303	0.0424	0.0014	0.0000	0.0269	0.0000	0.0000
EOCC_018	0.7929	0.1852	0.0220	0.0000	0.0000	0.0000	0.0000	0.0000
EOCC_019	0.9169	0.0096	0.0646	0.0089	0.0000	0.0000	0.0000	0.0000
EOCC_020	0.7456	0.1840	0.0417	0.0078	0.0000	0.0199	0.0000	0.0011
EOCC_021	0.8836	0.0338	0.0748	0.0079	0.0000	0.0000	0.0000	0.0000
EOCC_022	0.7931	0.1365	0.0664	0.0039	0.0000	0.0000	0.0000	0.0000
EOCC_023	0.8172	0.0000	0.0000	0.0005	0.1823	0.0000	0.0000	0.0000
EOCC_024	0.9558	0.0000	0.0409	0.0006	0.0000	0.0000	0.0027	0.0000
EOCC_025	0.9295	0.0355	0.0343	0.0007	0.0000	0.0000	0.0000	0.0000
EOCC_026	0.7242	0.1986	0.0664	0.0043	0.0000	0.0065	0.0000	0.0000
EOCC_027	0.5985	0.2564	0.0943	0.0286	0.0000	0.0222	0.0000	0.0000
EOCC_028	0.8319	0.0923	0.0724	0.0016	0.0000	0.0000	0.0019	0.0000
EOCC_029	0.8612	0.0932	0.0423	0.0033	0.0000	0.0000	0.0000	0.0000
EOCC_030	0.8451	0.0768	0.0682	0.0099	0.0000	0.0000	0.0000	0.0000
EOCC_031	0.8294	0.1362	0.0309	0.0035	0.0000	0.0000	0.0000	0.0000
EOCC_032	0.8470	0.1005	0.0344	0.0070	0.0000	0.0000	0.0092	0.0019
EOCC_033	0.7007	0.2435	0.0434	0.0093	0.0000	0.0000	0.0031	0.0000
EOCC_034	0.8697	0.0341	0.0682	0.0128	0.0000	0.0110	0.0000	0.0042
EOCC_035	0.7650	0.1191	0.0640	0.0284	0.0000	0.0186	0.0000	0.0048
EOCC_036	0.8426	0.1051	0.0512	0.0010	0.0000	0.0000	0.0000	0.0001
EOCC_037	0.8323	0.0595	0.0882	0.0201	0.0000	0.0000	0.0000	0.0000
EOCC_038	0.7528	0.1660	0.0735	0.0077	0.0000	0.0000	0.0000	0.0000
EOCC_039	0.8730	0.0470	0.0690	0.0110	0.0000	0.0000	0.0000	0.0000
EOCC_040	0.7759	0.2046	0.0188	0.0000	0.0000	0.0000	0.0007	0.0000
EOCC_041	0.8301	0.0698	0.0896	0.0106	0.0000	0.0000	0.0000	0.0000
EOCC_042	0.6659	0.0392	0.0435	0.0000	0.2398	0.0110	0.0000	0.0007
EOCC_043	0.8746	0.0469	0.0526	0.0000	0.0000	0.0000	0.0246	0.0013
EOCC_044	0.8808	0.0666	0.0480	0.0000	0.0000	0.0046	0.0000	0.0000
EOCC_045	0.6993	0.2056	0.0737	0.0000	0.0000	0.0215	0.0000	0.0000
EOCC_046	0.9093	0.0150	0.0732	0.0000	0.0000	0.0000	0.0000	0.0026
EOCC_047	0.6652	0.1530	0.1158	0.0397	0.0000	0.0000	0.0263	0.0000
EOCC_048	0.9228	0.0433	0.0337	0.0000	0.0000	0.0000	0.0000	0.0001
EOCC_049	0.8215	0.0794	0.0630	0.0281	0.0000	0.0080	0.0000	0.0000
EOCC_050	0.5421	0.2813	0.0995	0.0478	0.0000	0.0294	0.0000	0.0000
EOCC_051	0.8497	0.0984	0.0485	0.0001	0.0000	0.0034	0.0000	0.0000
EOCC_052	0.9066	0.0376	0.0537	0.0021	0.0000	0.0000	0.0000	0.0000
EOCC_053	0.7809	0.0761	0.0977	0.0093	0.0000	0.0359	0.0000	0.0000
LOCC_001	0.9932	0.0046	0.0000	0.0022	0.0000	0.0000	0.0000	0.0000
LOCC_002	0.8857	0.0145	0.0851	0.0147	0.0000	0.0000	0.0000	0.0000
LOCC_003	0.7318	0.1533	0.0961	0.0165	0.0000	0.0000	0.0024	0.0000
LOCC_004	0.7397	0.0293	0.0000	0.0057	0.2046	0.0207	0.0000	0.0000
LOCC_005	0.8567	0.0724	0.0267	0.0442	0.0000	0.0000	0.0000	0.0000
LOCC_006	0.9034	0.0750	0.0139	0.0004	0.0000	0.0000	0.0071	0.0002
LOCC_007	0.7697	0.0743	0.1273	0.0046	0.0000	0.0233	0.0000	0.0008
LOCC_008	0.8096	0.0885	0.0910	0.0028	0.0000	0.0082	0.0000	0.0000
LOCC_009	0.8815	0.0491	0.0681	0.0013	0.0000	0.0000	0.0000	0.0001
LOCC_010	0.9188	0.0280	0.0509	0.0022	0.0000	0.0000	0.0000	0.0000
LOCC_011	0.7737	0.1520	0.0603	0.0053	0.0000	0.0087	0.0000	0.0000
LOCC_012	0.8208	0.1110	0.0541	0.0107	0.0000	0.0000	0.0025	0.0010
LOCC_013	0.8262	0.1264	0.0299	0.0175	0.0000	0.0000	0.0000	0.0000
LOCC_014	0.9373	0.0470	0.0086	0.0008	0.0000	0.0063	0.0000	0.0000
LOCC_015	0.8878	0.0768	0.0320	0.0000	0.0000	0.0000	0.0023	0.0010

LOCC_016	0.9188	0.0350	0.0455	0.0006	0.0000	0.0000	0.0000	0.0000
LOCC_017	0.7584	0.0950	0.0886	0.0150	0.0000	0.0376	0.0000	0.0053
LOCC_018	0.8877	0.0543	0.0356	0.0000	0.0210	0.0000	0.0014	0.0000
LOCC_019	0.9159	0.0516	0.0280	0.0044	0.0000	0.0000	0.0000	0.0000
LOCC_020	0.9055	0.0532	0.0398	0.0008	0.0000	0.0000	0.0006	0.0002
LOCC_021	0.7627	0.0513	0.1280	0.0230	0.0000	0.0327	0.0000	0.0024
LOCC_022	0.6881	0.1148	0.1114	0.0000	0.0000	0.0829	0.0000	0.0028
LOCC_023	0.8238	0.1116	0.0624	0.0020	0.0000	0.0000	0.0000	0.0002
LOCC_024	0.7499	0.1179	0.0754	0.0210	0.0000	0.0358	0.0000	0.0000
LOCC_025	0.9376	0.0423	0.0201	0.0000	0.0000	0.0000	0.0000	0.0000
LOCC_026	0.7696	0.1181	0.0565	0.0045	0.0000	0.0325	0.0180	0.0008
LOCC_027	0.6594	0.2245	0.0892	0.0104	0.0000	0.0128	0.0000	0.0037
LOCC_028	0.8387	0.0908	0.0578	0.0001	0.0000	0.0125	0.0000	0.0000
LOCC_029	0.8894	0.0674	0.0407	0.0025	0.0000	0.0000	0.0000	0.0000
LOCC_030	0.8009	0.0909	0.1082	0.0000	0.0000	0.0000	0.0000	0.0000
LOCC_031	0.7872	0.0881	0.0828	0.0419	0.0000	0.0000	0.0000	0.0000
LOCC_032	0.7710	0.1839	0.0441	0.0011	0.0000	0.0000	0.0000	0.0000
LOCC_033	0.8514	0.0880	0.0567	0.0012	0.0000	0.0000	0.0021	0.0006
LOCC_034	0.9288	0.0070	0.0634	0.0000	0.0000	0.0002	0.0000	0.0006
LOCC_035	0.8914	0.0320	0.0766	0.0000	0.0000	0.0000	0.0000	0.0000
LOCC_036	0.7085	0.1025	0.1262	0.0186	0.0000	0.0388	0.0000	0.0054
LOCC_037	0.7770	0.1214	0.0851	0.0071	0.0000	0.0095	0.0000	0.0000
LOCC_038	0.9720	0.0066	0.0205	0.0000	0.0000	0.0000	0.0000	0.0009
LOCC_039	0.7592	0.1556	0.0851	0.0001	0.0000	0.0000	0.0000	0.0000
LOCC_040	0.9522	0.0139	0.0315	0.0024	0.0000	0.0000	0.0000	0.0000
LOCC_041	0.9619	0.0117	0.0241	0.0024	0.0000	0.0000	0.0000	0.0000
LOCC_042	0.9561	0.0000	0.0437	0.0002	0.0000	0.0000	0.0000	0.0000
LOCC_043	0.6690	0.2075	0.1046	0.0189	0.0000	0.0000	0.0000	0.0000
LOCC_044	0.7338	0.1298	0.1205	0.0145	0.0000	0.0000	0.0000	0.0014
LOCC_045	0.9542	0.0216	0.0180	0.0062	0.0000	0.0000	0.0000	0.0000
LOCC_046	0.7178	0.1229	0.1159	0.0087	0.0000	0.0348	0.0000	0.0000
LOCC_047	0.4915	0.4181	0.0751	0.0154	0.0000	0.0000	0.0000	0.0000
LOCC_048	0.7969	0.0436	0.0988	0.0225	0.0000	0.0242	0.0000	0.0140
LOCC_049	0.8191	0.1104	0.0470	0.0000	0.0000	0.0000	0.0194	0.0041
LOCC_050	0.8646	0.0871	0.0404	0.0079	0.0000	0.0000	0.0000	0.0000
LOCC_051	0.8535	0.0667	0.0781	0.0017	0.0000	0.0000	0.0000	0.0000
LOCC_052	0.6773	0.0172	0.1369	0.0242	0.1404	0.0000	0.0040	0.0000
LOCC_053	0.8486	0.0155	0.1247	0.0077	0.0000	0.0000	0.0033	0.0002
LOCC_054	0.8124	0.0171	0.1017	0.0135	0.0000	0.0445	0.0077	0.0031
LOCC_055	0.6846	0.2384	0.0635	0.0057	0.0000	0.0078	0.0000	0.0000
LOCC_056	0.8337	0.0409	0.1177	0.0028	0.0000	0.0048	0.0000	0.0001
LOCC_057	0.9671	0.0193	0.0136	0.0000	0.0000	0.0000	0.0000	0.0000
LOCC_058	0.9661	0.0172	0.0129	0.0039	0.0000	0.0000	0.0000	0.0000
LOCC_059	0.9780	0.0179	0.0000	0.0021	0.0000	0.0000	0.0020	0.0000
LOCC_060	0.9091	0.0452	0.0413	0.0043	0.0000	0.0000	0.0000	0.0000
LOCC_061	0.8899	0.0432	0.0645	0.0021	0.0000	0.0000	0.0000	0.0003
LOCC_062	0.8706	0.0767	0.0459	0.0068	0.0000	0.0000	0.0000	0.0000
LOCC_063	0.8384	0.0646	0.0770	0.0184	0.0000	0.0001	0.0000	0.0015
LOCC_064	0.7051	0.2342	0.0571	0.0005	0.0000	0.0000	0.0031	0.0000
LOCC_065	0.5881	0.2065	0.1717	0.0075	0.0000	0.0037	0.0225	0.0000
LOCC_066	0.8266	0.0490	0.1220	0.0024	0.0000	0.0000	0.0000	0.0000
LOCC_067	0.7940	0.0661	0.1350	0.0049	0.0000	0.0000	0.0000	0.0000
LOCC_068	0.7312	0.0675	0.1593	0.0152	0.0000	0.0245	0.0000	0.0023
LOCC_069	0.8411	0.0328	0.1166	0.0095	0.0000	0.0000	0.0000	0.0000
LOCC_070	0.8181	0.0588	0.1204	0.0000	0.0000	0.0000	0.0000	0.0027
LOCC_071	0.6206	0.2477	0.0838	0.0171	0.0198	0.0106	0.0004	0.0000
LOCC_072	0.8015	0.0575	0.0880	0.0244	0.0000	0.0287	0.0000	0.0000
LOCC_073	0.6435	0.2376	0.1000	0.0102	0.0000	0.0088	0.0000	0.0000
LOCC_074	0.9065	0.0516	0.0325	0.0018	0.0000	0.0000	0.0052	0.0023
LOCC_075	0.8534	0.0779	0.0662	0.0002	0.0000	0.0000	0.0023	0.0000
LOCC_076	0.8429	0.0471	0.1039	0.0050	0.0000	0.0000	0.0000	0.0011
LOCC_077	0.9646	0.0063	0.0288	0.0002	0.0000	0.0000	0.0000	0.0001
LOCC_078	0.9324	0.0336	0.0328	0.0010	0.0000	0.0000	0.0000	0.0002
LOCC_079	0.9390	0.0341	0.0202	0.0023	0.0026	0.0000	0.0017	0.0000
LOCC_080	0.9534	0.0371	0.0000	0.0037	0.0059	0.0000	0.0000	0.0000
LOCC_081	0.8580	0.0628	0.0589	0.0092	0.0000	0.0000	0.0110	0.0000
LOCC_082	0.6099	0.2519	0.0742	0.0074	0.0525	0.0000	0.0042	0.0000
LOCC_083	0.9467	0.0237	0.0274	0.0022	0.0000	0.0000	0.0000	0.0000
LOCC_084	0.9305	0.0108	0.0505	0.0082	0.0000	0.0000	0.0000	0.0000
LOCC_085	0.8205	0.0715	0.1071	0.0009	0.0000	0.0000	0.0000	0.0000
LOCC_086	0.7199	0.1628	0.0956	0.0079	0.0000	0.0138	0.0000	0.0000

LOCC_087	0.9433	0.0193	0.0308	0.0051	0.0000	0.0000	0.0015	0.0000
LOCC_088	0.7756	0.0540	0.1177	0.0053	0.0000	0.0474	0.0000	0.0000
LOCC_089	0.7837	0.0820	0.1319	0.0014	0.0000	0.0011	0.0000	0.0000
LOCC_090	0.8764	0.0583	0.0506	0.0054	0.0000	0.0000	0.0094	0.0000
LOCC_091	0.8078	0.1131	0.0771	0.0000	0.0000	0.0019	0.0000	0.0001
LOCC_092	0.8823	0.0330	0.0824	0.0020	0.0000	0.0000	0.0000	0.0003
LOCC_093	0.7929	0.0184	0.0003	0.0192	0.1614	0.0049	0.0000	0.0030
LOCC_094	0.7502	0.1973	0.0501	0.0000	0.0000	0.0000	0.0025	0.0000
LOCC_095	0.3519	0.3200	0.0944	0.0303	0.1351	0.0683	0.0000	0.0000
LOCC_096	0.7103	0.1799	0.0670	0.0428	0.0000	0.0000	0.0000	0.0000
LOCC_097	0.8938	0.0171	0.0843	0.0037	0.0000	0.0000	0.0000	0.0011
LOCC_098	0.7744	0.1778	0.0450	0.0014	0.0000	0.0000	0.0014	0.0000
LOCC_099	0.7417	0.0992	0.1256	0.0284	0.0000	0.0051	0.0000	0.0000
LOCC_100	0.8523	0.0383	0.1085	0.0004	0.0000	0.0000	0.0005	0.0000
LOCC_101	0.7045	0.1926	0.0788	0.0230	0.0000	0.0000	0.0012	0.0000
LOCC_102	0.9151	0.0335	0.0463	0.0051	0.0000	0.0000	0.0000	0.0000
LOCC_103	0.9385	0.0130	0.0418	0.0067	0.0000	0.0000	0.0000	0.0000
LOCC_104	0.7978	0.1187	0.0631	0.0184	0.0000	0.0020	0.0000	0.0000
LOCC_105	0.7577	0.1150	0.1121	0.0074	0.0000	0.0078	0.0000	0.0000
LOCC_106	0.4675	0.4047	0.1060	0.0143	0.0000	0.0021	0.0054	0.0000
LOCC_107	0.6934	0.1146	0.0870	0.0000	0.0621	0.0414	0.0000	0.0015
LOCC_108	0.8764	0.0308	0.0876	0.0044	0.0000	0.0001	0.0000	0.0006
LOCC_109	0.9897	0.0103	0.0000	0.0000	0.0000	0.0000	0.0000	0.0000
LOCC_110	0.8623	0.0558	0.0616	0.0101	0.0000	0.0101	0.0000	0.0000
LOCC_111	0.8741	0.0623	0.0615	0.0000	0.0022	0.0000	0.0000	0.0000
LOCC_112	0.8875	0.0403	0.0719	0.0000	0.0000	0.0000	0.0000	0.0003
LOCC_113	0.4274	0.4888	0.0717	0.0085	0.0000	0.0035	0.0000	0.0000
LOCC_114	0.8269	0.0176	0.0258	0.0000	0.1297	0.0000	0.0000	0.0000
LOCC_115	0.8650	0.0498	0.0830	0.0021	0.0000	0.0000	0.0000	0.0000
LOCC_116	0.8565	0.1086	0.0237	0.0111	0.0000	0.0000	0.0000	0.0000
LOCC_117	0.8127	0.1405	0.0214	0.0038	0.0000	0.0000	0.0201	0.0014
LOCC_118	0.7256	0.2025	0.0649	0.0069	0.0000	0.0000	0.0000	0.0000
LOCC_119	0.7502	0.0900	0.0754	0.0168	0.0155	0.0475	0.0000	0.0046
LOCC_120	0.9293	0.0385	0.0321	0.0000	0.0000	0.0000	0.0000	0.0001
LOCC_121	0.9053	0.0217	0.0621	0.0109	0.0000	0.0000	0.0000	0.0000
LOCC_122	0.8465	0.1001	0.0330	0.0084	0.0000	0.0000	0.0082	0.0038
LOCC_123	0.8702	0.0650	0.0646	0.0003	0.0000	0.0000	0.0000	0.0000
LOCC_124	0.8256	0.0363	0.1182	0.0051	0.0000	0.0145	0.0000	0.0003
LOCC_125	0.9048	0.0507	0.0426	0.0013	0.0000	0.0000	0.0000	0.0005
LOCC_126	0.8600	0.0918	0.0351	0.0000	0.0113	0.0019	0.0000	0.0000
LOCC_127	0.8385	0.0786	0.0780	0.0047	0.0000	0.0000	0.0000	0.0002
LOCC_128	0.8056	0.0533	0.0806	0.0183	0.0000	0.0395	0.0000	0.0027
LOCC_129	0.7333	0.1180	0.0945	0.0050	0.0000	0.0492	0.0000	0.0000
LOCC_130	0.4832	0.3782	0.0646	0.0291	0.0000	0.0449	0.0000	0.0000
LOCC_131	0.6917	0.2016	0.0939	0.0106	0.0000	0.0022	0.0000	0.0000
LOCC_132	0.6610	0.2343	0.0786	0.0204	0.0000	0.0000	0.0057	0.0000
LOCC_133	0.5264	0.2861	0.0959	0.0218	0.0000	0.0697	0.0000	0.0000
LOCC_134	0.9195	0.0342	0.0425	0.0037	0.0000	0.0000	0.0002	0.0000
LOCC_135	0.8675	0.0655	0.0640	0.0023	0.0000	0.0000	0.0000	0.0007
LOCC_136	0.9472	0.0064	0.0460	0.0004	0.0000	0.0000	0.0000	0.0000
LOCC_137	0.6982	0.0944	0.1053	0.0518	0.0000	0.0460	0.0000	0.0042
LOCC_138	0.9626	0.0153	0.0221	0.0000	0.0000	0.0000	0.0000	0.0000
LOCC_139	0.8488	0.0787	0.0221	0.0000	0.0471	0.0000	0.0033	0.0000
LOCC_140	0.8793	0.0326	0.0696	0.0031	0.0000	0.0150	0.0000	0.0004
LOCC_141	0.8712	0.0703	0.0461	0.0009	0.0000	0.0000	0.0115	0.0000
LOCC_142	0.6790	0.2033	0.1088	0.0029	0.0048	0.0000	0.0000	0.0013
LOCC_143	0.8688	0.0568	0.0687	0.0020	0.0000	0.0037	0.0000	0.0000
LOCC_144	0.7741	0.1360	0.0822	0.0074	0.0000	0.0000	0.0000	0.0003
LOCC_145	0.7108	0.1742	0.0795	0.0163	0.0000	0.0151	0.0000	0.0041
LOCC_146	0.7696	0.1263	0.0832	0.0100	0.0000	0.0109	0.0000	0.0000
LOCC_147	0.8773	0.0328	0.0601	0.0104	0.0000	0.0157	0.0000	0.0037
LOCC_148	0.8653	0.0506	0.0771	0.0024	0.0000	0.0043	0.0000	0.0003
LOCC_149	0.9296	0.0000	0.0653	0.0040	0.0000	0.0000	0.0000	0.0011
LOCC_150	0.8392	0.1504	0.0064	0.0041	0.0000	0.0000	0.0000	0.0000
LOCC_151	0.9264	0.0390	0.0305	0.0041	0.0000	0.0000	0.0000	0.0000
LOCC_152	0.8177	0.1180	0.0642	0.0000	0.0000	0.0000	0.0000	0.0001
LOCC_153	0.8433	0.1418	0.0098	0.0000	0.0000	0.0000	0.0052	0.0000
LOCC_154	0.4533	0.4659	0.0692	0.0115	0.0000	0.0000	0.0000	0.0000
LOCC_155	0.8911	0.0305	0.0768	0.0000	0.0000	0.0003	0.0000	0.0012
LOCC_156	0.9551	0.0168	0.0213	0.0068	0.0000	0.0000	0.0000	0.0000
LOCC_157	0.7579	0.1702	0.0667	0.0028	0.0000	0.0000	0.0025	0.0000

LOCC_158	0.9197	0.0192	0.0610	0.0000	0.0000	0.0000	0.0000	0.0000
LOCC_159	0.8600	0.0869	0.0431	0.0100	0.0000	0.0000	0.0000	0.0000
LOCC_160	0.7994	0.1163	0.0614	0.0066	0.0000	0.0163	0.0000	0.0000
LOCC_161	0.9353	0.0166	0.0404	0.0075	0.0000	0.0000	0.0000	0.0003
LOCC_162	0.8873	0.0639	0.0396	0.0016	0.0000	0.0000	0.0053	0.0023
LOCC_163	0.7257	0.1994	0.0697	0.0051	0.0000	0.0000	0.0000	0.0000
LOCC_164	0.8242	0.0529	0.1191	0.0029	0.0000	0.0003	0.0000	0.0006
LOCC_165	0.7092	0.2137	0.0692	0.0074	0.0000	0.0000	0.0000	0.0004
LOCC_166	0.8407	0.0927	0.0664	0.0002	0.0000	0.0000	0.0000	0.0001
LOCC_167	0.8434	0.0781	0.0658	0.0062	0.0000	0.0057	0.0000	0.0009
LOCC_168	0.9573	0.0156	0.0253	0.0019	0.0000	0.0000	0.0000	0.0000
LOCC_169	0.7512	0.1987	0.0474	0.0027	0.0000	0.0000	0.0000	0.0000
LOCC_170	0.9798	0.0031	0.0125	0.0046	0.0000	0.0000	0.0000	0.0000
LOCC_171	0.8468	0.0786	0.0696	0.0027	0.0000	0.0023	0.0000	0.0000
LOCC_172	0.8676	0.0871	0.0269	0.0059	0.0000	0.0000	0.0126	0.0000
LOCC_173	0.7763	0.1118	0.0874	0.0009	0.0000	0.0234	0.0000	0.0002
LOCC_174	0.8474	0.0437	0.0901	0.0130	0.0000	0.0058	0.0000	0.0000
LOCC_175	0.8860	0.0688	0.0375	0.0077	0.0000	0.0000	0.0000	0.0000
LOCC_176	0.9118	0.0204	0.0457	0.0204	0.0000	0.0000	0.0000	0.0016
LOCC_177	0.8613	0.0899	0.0446	0.0034	0.0000	0.0000	0.0000	0.0007
LOCC_178	0.8696	0.0520	0.0544	0.0152	0.0000	0.0089	0.0000	0.0000
LOCC_179	0.9088	0.0160	0.0667	0.0077	0.0000	0.0009	0.0000	0.0000
LOCC_180	0.6486	0.2698	0.0740	0.0077	0.0000	0.0000	0.0000	0.0000
LOCC_181	0.6205	0.2524	0.0776	0.0114	0.0000	0.0381	0.0000	0.0000
LOCC_182	0.8599	0.1066	0.0316	0.0018	0.0000	0.0000	0.0000	0.0000
LOCC_183	0.8744	0.0703	0.0539	0.0013	0.0000	0.0000	0.0000	0.0000
LOCC_184	0.7699	0.1782	0.0513	0.0005	0.0000	0.0000	0.0000	0.0000
LOCC_185	0.9601	0.0386	0.0000	0.0011	0.0000	0.0000	0.0000	0.0001
LOCC_186	0.8787	0.0995	0.0212	0.0000	0.0000	0.0000	0.0006	0.0000
LOCC_187	0.4867	0.3079	0.1661	0.0150	0.0000	0.0243	0.0000	0.0000
LOCC_188	0.7501	0.1124	0.1148	0.0000	0.0000	0.0000	0.0196	0.0031
LOCC_189	0.9163	0.0836	0.0000	0.0001	0.0000	0.0000	0.0000	0.0000
LOCC_190	0.9730	0.0000	0.0257	0.0013	0.0000	0.0000	0.0000	0.0000
LOCC_191	0.7386	0.1247	0.0932	0.0085	0.0000	0.0324	0.0000	0.0026
LOCC_192	0.8913	0.0511	0.0507	0.0064	0.0000	0.0000	0.0003	0.0001
LOCC_193	0.7643	0.0798	0.1148	0.0000	0.0000	0.0377	0.0000	0.0034
LOCC_194	0.8432	0.0706	0.0832	0.0009	0.0000	0.0021	0.0000	0.0000
LOCC_195	0.7937	0.0992	0.0885	0.0045	0.0000	0.0137	0.0000	0.0004
LOCC_196	0.5069	0.3798	0.1071	0.0063	0.0000	0.0000	0.0000	0.0000
LOCC_197	0.8721	0.0916	0.0082	0.0268	0.0000	0.0000	0.0013	0.0000
LOCC_198	0.9260	0.0354	0.0348	0.0036	0.0000	0.0000	0.0000	0.0001
LOCC_199	0.9865	0.0079	0.0000	0.0000	0.0000	0.0000	0.0056	0.0000
LOCC_200	0.8526	0.0725	0.0718	0.0011	0.0000	0.0020	0.0000	0.0000
LOCC_201	0.8810	0.0682	0.0482	0.0027	0.0000	0.0000	0.0000	0.0000
LOCC_202	0.8581	0.0675	0.0000	0.0018	0.0642	0.0000	0.0083	0.0000
LOCC_203	0.9691	0.0136	0.0164	0.0007	0.0000	0.0000	0.0000	0.0002
LOCC_204	0.9674	0.0156	0.0082	0.0088	0.0000	0.0000	0.0000	0.0000
LOCC_205	0.9310	0.0266	0.0380	0.0000	0.0000	0.0000	0.0044	0.0000
LOCC_206	0.9018	0.0397	0.0573	0.0011	0.0000	0.0000	0.0000	0.0000
LOCC_207	0.5928	0.2420	0.1067	0.0393	0.0000	0.0192	0.0000	0.0000
LOCC_208	0.8854	0.0535	0.0413	0.0035	0.0000	0.0149	0.0015	0.0000
LOCC_209	0.9290	0.0074	0.0495	0.0141	0.0000	0.0000	0.0000	0.0000
LOCC_210	0.8750	0.0363	0.0327	0.0374	0.0000	0.0000	0.0185	0.0000
LOCC_211	0.8297	0.1020	0.0620	0.0000	0.0000	0.0055	0.0008	0.0000
LOCC_212	0.7613	0.1148	0.0996	0.0038	0.0000	0.0205	0.0000	0.0000
LOCC_213	0.6516	0.2692	0.0779	0.0014	0.0000	0.0000	0.0000	0.0000
LOCC_214	0.7802	0.1536	0.0569	0.0093	0.0000	0.0000	0.0000	0.0000
LOCC_215	0.7375	0.1989	0.0566	0.0071	0.0000	0.0000	0.0000	0.0000
LOCC_216	0.6449	0.2191	0.0967	0.0367	0.0000	0.0000	0.0025	0.0000
LOCC_217	0.8837	0.0315	0.0795	0.0047	0.0000	0.0000	0.0000	0.0006
LOCC_218	0.8460	0.0789	0.0745	0.0000	0.0000	0.0000	0.0000	0.0006
LOCC_219	0.9058	0.0528	0.0387	0.0026	0.0000	0.0000	0.0000	0.0000
LOCC_220	0.8252	0.1395	0.0314	0.0039	0.0000	0.0000	0.0000	0.0000
LOCC_221	0.8417	0.0883	0.0494	0.0206	0.0000	0.0000	0.0000	0.0000
LOCC_222	0.7780	0.1763	0.0440	0.0000	0.0000	0.0000	0.0017	0.0000
LOCC_223	0.7754	0.1165	0.0631	0.0089	0.0000	0.0356	0.0000	0.0004
LOCC_224	0.8876	0.0631	0.0486	0.0003	0.0000	0.0000	0.0000	0.0004
LOCC_225	0.8884	0.0433	0.0673	0.0000	0.0000	0.0000	0.0000	0.0010
LOCC_226	0.8404	0.1032	0.0460	0.0041	0.0000	0.0062	0.0000	0.0000
LOCC_227	0.9615	0.0022	0.0344	0.0019	0.0000	0.0000	0.0000	0.0000
LOCC_228	0.8848	0.0710	0.0411	0.0025	0.0000	0.0005	0.0000	0.0000

LOCC_229	0.8071	0.1101	0.0683	0.0145	0.0000	0.0000	0.0000	0.0000
LOCC_230	0.2679	0.5166	0.1593	0.0212	0.0000	0.0351	0.0000	0.0000
LOCC_231	0.9649	0.0253	0.0086	0.0007	0.0000	0.0000	0.0000	0.0005
LOCC_232	0.9336	0.0480	0.0139	0.0043	0.0000	0.0000	0.0000	0.0001
LOCC_233	0.7581	0.1823	0.0430	0.0054	0.0000	0.0106	0.0007	0.0000
LOCC_234	0.9020	0.0676	0.0285	0.0007	0.0000	0.0000	0.0000	0.0011
LOCC_235	0.9202	0.0365	0.0433	0.0000	0.0000	0.0000	0.0000	0.0000
LOCC_236	0.9218	0.0507	0.0109	0.0000	0.0000	0.0000	0.0159	0.0008
LOCC_237	0.6927	0.1742	0.1247	0.0027	0.0000	0.0057	0.0000	0.0000
LOCC_238	0.8117	0.0797	0.0968	0.0118	0.0000	0.0000	0.0000	0.0000
LOCC_239	0.9143	0.0000	0.0486	0.0091	0.0000	0.0280	0.0000	0.0000
LOCC_240	0.6264	0.2715	0.0927	0.0077	0.0000	0.0016	0.0000	0.0000
LOCC_241	0.8677	0.0537	0.0785	0.0000	0.0000	0.0000	0.0000	0.0000
LOCC_242	0.8350	0.0855	0.0738	0.0004	0.0000	0.0050	0.0000	0.0002
LOCC_243	0.5607	0.0420	0.2735	0.0155	0.0000	0.0979	0.0103	0.0000
LOCC_244	0.8468	0.0682	0.0653	0.0049	0.0000	0.0141	0.0000	0.0007
LOCC_245	0.8563	0.1319	0.0005	0.0071	0.0000	0.0000	0.0041	0.0000
LOCC_246	0.9672	0.0316	0.0000	0.0010	0.0000	0.0000	0.0000	0.0002
LOCC_247	0.7502	0.2231	0.0251	0.0016	0.0000	0.0000	0.0000	0.0000
LOCC_248	0.8965	0.0228	0.0798	0.0004	0.0000	0.0000	0.0000	0.0005
LOCC_249	0.7814	0.0823	0.1154	0.0000	0.0000	0.0210	0.0000	0.0000
LOCC_250	0.5527	0.2243	0.1583	0.0163	0.0000	0.0484	0.0000	0.0000
LOCC_251	0.8555	0.0809	0.0636	0.0000	0.0000	0.0000	0.0000	0.0000
LOCC_252	0.8598	0.0934	0.0353	0.0080	0.0000	0.0000	0.0020	0.0015
LOCC_253	0.8248	0.0588	0.1106	0.0058	0.0000	0.0000	0.0000	0.0000
LOCC_254	0.6527	0.1646	0.1733	0.0000	0.0000	0.0094	0.0000	0.0000
LOCC_255	0.7846	0.0900	0.1089	0.0107	0.0000	0.0058	0.0000	0.0000
LOCC_256	0.8486	0.0662	0.0790	0.0000	0.0000	0.0062	0.0000	0.0000
LOCC_257	0.8899	0.0688	0.0250	0.0017	0.0000	0.0000	0.0120	0.0026
LOCC_258	0.8663	0.0097	0.1090	0.0123	0.0000	0.0000	0.0026	0.0000
LOCC_259	0.7365	0.0514	0.1825	0.0111	0.0000	0.0185	0.0000	0.0001
LOCC_260	0.7631	0.1305	0.0924	0.0000	0.0000	0.0141	0.0000	0.0000
LOCC_261	0.8043	0.0739	0.1195	0.0016	0.0000	0.0004	0.0000	0.0003
LOCC_262	0.9067	0.0402	0.0313	0.0000	0.0152	0.0062	0.0000	0.0004
LOCC_263	0.8282	0.1407	0.0270	0.0041	0.0000	0.0000	0.0000	0.0000
LOCC_264	0.6792	0.1842	0.1218	0.0147	0.0000	0.0000	0.0000	0.0000
LOCC_265	0.7861	0.0747	0.1251	0.0000	0.0000	0.0133	0.0000	0.0009
LOCC_266	0.9559	0.0260	0.0168	0.0013	0.0000	0.0000	0.0000	0.0000
LOCC_267	0.8037	0.1159	0.0612	0.0055	0.0000	0.0000	0.0130	0.0007
LOCC_268	0.8384	0.0910	0.0681	0.0015	0.0000	0.0000	0.0000	0.0010
LOCC_269	0.7856	0.1442	0.0644	0.0052	0.0000	0.0000	0.0000	0.0005
LOCC_270	0.9812	0.0158	0.0011	0.0019	0.0000	0.0000	0.0000	0.0000
LOCC_271	0.8743	0.0744	0.0514	0.0000	0.0000	0.0000	0.0000	0.0000
LOCC_272	0.9075	0.0641	0.0181	0.0047	0.0000	0.0056	0.0000	0.0000
LOCC_273	0.8474	0.0268	0.0958	0.0248	0.0000	0.0000	0.0025	0.0027
LOCC_274	0.8916	0.0608	0.0457	0.0019	0.0000	0.0000	0.0000	0.0000
LOCC_275	0.9057	0.0207	0.0651	0.0085	0.0000	0.0000	0.0000	0.0000
LOCC_276	0.9544	0.0315	0.0031	0.0109	0.0000	0.0000	0.0000	0.0000
LOCC_277	0.7374	0.1605	0.0943	0.0076	0.0000	0.0001	0.0000	0.0000
LOCC_278	0.8456	0.0515	0.0748	0.0280	0.0000	0.0000	0.0000	0.0000
LOCC_279	0.9044	0.0300	0.0634	0.0000	0.0000	0.0000	0.0015	0.0007
LOCC_280	0.9425	0.0236	0.0337	0.0003	0.0000	0.0000	0.0000	0.0000
LOCC_281	0.8143	0.1458	0.0355	0.0000	0.0000	0.0000	0.0043	0.0000
LOCC_282	0.8930	0.0540	0.0502	0.0012	0.0000	0.0011	0.0000	0.0004
LOCC_283	0.7135	0.2175	0.0669	0.0019	0.0000	0.0000	0.0000	0.0002
LOCC_284	0.8808	0.0576	0.0564	0.0019	0.0000	0.0000	0.0033	0.0000
LOCC_285	0.6809	0.1844	0.0836	0.0257	0.0000	0.0255	0.0000	0.0000
LOCC_286	0.8348	0.1231	0.0355	0.0000	0.0000	0.0066	0.0000	0.0000
LOCC_287	0.9736	0.0162	0.0093	0.0009	0.0000	0.0000	0.0000	0.0000
LOCC_288	0.9268	0.0231	0.0501	0.0000	0.0000	0.0000	0.0000	0.0000
LOCC_289	1.0000	0.0000	0.0000	0.0000	0.0000	0.0000	0.0000	0.0000
LOCC_290	0.8667	0.0481	0.0679	0.0029	0.0000	0.0000	0.0144	0.0000
LOCC_291	0.7886	0.0981	0.1048	0.0085	0.0000	0.0000	0.0000	0.0000
LOCC_292	0.7089	0.1431	0.0661	0.0526	0.0017	0.0275	0.0000	0.0000
LOCC_293	0.7728	0.1493	0.0727	0.0035	0.0000	0.0000	0.0017	0.0000
LOCC_294	0.8566	0.0507	0.0781	0.0146	0.0000	0.0000	0.0000	0.0000
LOCC_295	0.8136	0.1142	0.0594	0.0098	0.0000	0.0013	0.0017	0.0000
LOCC_296	0.9348	0.0253	0.0379	0.0018	0.0000	0.0000	0.0000	0.0001
LOCC_297	0.8572	0.0694	0.0657	0.0076	0.0000	0.0000	0.0000	0.0000
LOCC_298	0.7576	0.2025	0.0400	0.0000	0.0000	0.0000	0.0000	0.0000
LOCC_299	0.8541	0.0524	0.0909	0.0005	0.0000	0.0021	0.0000	0.0000

LOCC_300	0.7800	0.0919	0.0975	0.0199	0.0000	0.0100	0.0000	0.0007
LOCC_301	0.9017	0.0256	0.0720	0.0000	0.0000	0.0000	0.0000	0.0007
LOCC_302	0.9655	0.0345	0.0000	0.0000	0.0000	0.0000	0.0000	0.0000
LOCC_303	0.8970	0.0727	0.0198	0.0085	0.0000	0.0000	0.0021	0.0000
LOCC_304	0.9032	0.0500	0.0463	0.0000	0.0000	0.0000	0.0000	0.0005
LOCC_305	0.7985	0.0742	0.1163	0.0063	0.0000	0.0048	0.0000	0.0000
LOCC_306	0.8892	0.0114	0.0933	0.0028	0.0000	0.0024	0.0000	0.0009
LOCC_307	0.8858	0.0338	0.0662	0.0142	0.0000	0.0000	0.0000	0.0000
LOCC_308	0.5667	0.2060	0.0661	0.0367	0.0000	0.0879	0.0367	0.0000
LOCC_309	0.8174	0.0741	0.1077	0.0008	0.0000	0.0000	0.0000	0.0000
LOCC_310	0.9424	0.0000	0.0483	0.0091	0.0000	0.0000	0.0000	0.0002
LOCC_311	0.7576	0.1617	0.0778	0.0014	0.0000	0.0015	0.0000	0.0000
LOCC_312	0.6783	0.0538	0.1302	0.0174	0.1129	0.0021	0.0000	0.0052
LOCC_313	0.8384	0.0937	0.0666	0.0013	0.0000	0.0000	0.0000	0.0000
LOCC_314	0.9206	0.0223	0.0539	0.0026	0.0000	0.0000	0.0000	0.0005
LOCC_315	0.8564	0.0411	0.0914	0.0035	0.0000	0.0077	0.0000	0.0000
LOCC_316	0.8828	0.0035	0.0368	0.0053	0.0716	0.0000	0.0000	0.0000
LOCC_317	0.9331	0.0133	0.0391	0.0145	0.0000	0.0000	0.0000	0.0000
LOCC_318	0.8860	0.0512	0.0589	0.0039	0.0000	0.0000	0.0000	0.0000
LOCC_319	0.7037	0.2368	0.0428	0.0000	0.0000	0.0167	0.0000	0.0000
LOCC_320	0.9627	0.0000	0.0325	0.0020	0.0000	0.0000	0.0026	0.0002
LOCC_321	0.7991	0.0640	0.1019	0.0088	0.0000	0.0205	0.0000	0.0056
LOCC_322	0.9002	0.0988	0.0004	0.0006	0.0000	0.0000	0.0000	0.0000
LOCC_323	0.8542	0.0414	0.0795	0.0144	0.0000	0.0000	0.0105	0.0000
LOCC_324	0.9205	0.0435	0.0303	0.0057	0.0000	0.0000	0.0000	0.0000
LOCC_325	0.6284	0.2652	0.0925	0.0032	0.0000	0.0106	0.0000	0.0000
LOCC_326	0.9545	0.0141	0.0278	0.0035	0.0000	0.0000	0.0000	0.0003
LOCC_327	0.7508	0.1569	0.0638	0.0087	0.0098	0.0101	0.0000	0.0000
LOCC_328	0.9057	0.0366	0.0537	0.0031	0.0000	0.0000	0.0000	0.0008
LOCC_329	0.9316	0.0518	0.0122	0.0043	0.0000	0.0000	0.0000	0.0000
LOCC_330	0.8461	0.0414	0.0921	0.0000	0.0000	0.0194	0.0000	0.0010
LOCC_331	0.9344	0.0266	0.0261	0.0031	0.0000	0.0000	0.0082	0.0015
LOCC_332	0.9144	0.0461	0.0370	0.0025	0.0000	0.0000	0.0000	0.0000
LOCC_333	0.7703	0.1100	0.1197	0.0000	0.0000	0.0000	0.0000	0.0000
LOCC_334	0.9265	0.0548	0.0142	0.0011	0.0000	0.0000	0.0033	0.0000
LOCC_335	0.6314	0.2468	0.1036	0.0079	0.0103	0.0000	0.0000	0.0000
LOCC_336	0.9508	0.0170	0.0316	0.0006	0.0000	0.0000	0.0000	0.0000
LOCC_337	0.8381	0.0650	0.0570	0.0129	0.0000	0.0270	0.0000	0.0000
LOCC_338	0.8789	0.0384	0.0776	0.0049	0.0000	0.0000	0.0002	0.0000
LOCC_339	0.8901	0.0674	0.0365	0.0039	0.0000	0.0000	0.0000	0.0021
LOCC_340	0.8739	0.0115	0.1121	0.0013	0.0000	0.0011	0.0000	0.0000
LOCC_341	0.7889	0.1280	0.0788	0.0043	0.0000	0.0000	0.0000	0.0000
LOCC_342	0.8633	0.0478	0.0802	0.0036	0.0000	0.0050	0.0000	0.0000
LOCC_343	0.8588	0.0061	0.0013	0.0000	0.1337	0.0000	0.0000	0.0000
LOCC_344	0.9129	0.0238	0.0566	0.0065	0.0000	0.0000	0.0000	0.0002
LOCC_345	0.9340	0.0234	0.0425	0.0000	0.0000	0.0000	0.0000	0.0000
LOCC_346	0.8785	0.0578	0.0446	0.0191	0.0000	0.0000	0.0000	0.0000
LOCC_347	0.8185	0.0536	0.1105	0.0088	0.0000	0.0059	0.0000	0.0027
LOCC_348	0.8390	0.0477	0.1082	0.0000	0.0000	0.0050	0.0000	0.0000
LOCC_349	0.8958	0.0264	0.0586	0.0140	0.0000	0.0000	0.0052	0.0000
LOCC_350	0.9507	0.0155	0.0241	0.0000	0.0000	0.0000	0.0086	0.0011
LOCC_351	0.8819	0.0816	0.0364	0.0001	0.0000	0.0000	0.0000	0.0000
LOCC_352	0.4434	0.3895	0.1275	0.0132	0.0000	0.0264	0.0000	0.0000
LOCC_353	0.7687	0.0987	0.1326	0.0000	0.0000	0.0000	0.0000	0.0000
LOCC_354	0.9409	0.0371	0.0216	0.0004	0.0000	0.0000	0.0000	0.0000
LOCC_355	0.7992	0.1710	0.0297	0.0000	0.0000	0.0000	0.0000	0.0001
LOCC_356	0.9333	0.0515	0.0129	0.0023	0.0000	0.0000	0.0000	0.0000
LOCC_357	0.9672	0.0262	0.0065	0.0000	0.0000	0.0000	0.0000	0.0002
LOCC_358	0.6779	0.1707	0.1189	0.0319	0.0000	0.0000	0.0006	0.0000
LOCC_359	0.8468	0.0211	0.1158	0.0146	0.0000	0.0000	0.0000	0.0017
LOCC_360	0.8108	0.0288	0.1555	0.0046	0.0000	0.0000	0.0000	0.0002
LOCC_361	0.5378	0.1489	0.1911	0.0715	0.0091	0.0408	0.0000	0.0008
LOCC_362	0.9568	0.0248	0.0132	0.0052	0.0000	0.0000	0.0000	0.0000
LOCC_363	0.4173	0.3171	0.0762	0.0177	0.1154	0.0493	0.0070	0.0000
LOCC_364	0.7131	0.2150	0.0497	0.0103	0.0000	0.0000	0.0119	0.0000
LOCC_365	0.8875	0.0219	0.0855	0.0051	0.0000	0.0000	0.0000	0.0000
LOCC_366	0.7874	0.1143	0.0937	0.0047	0.0000	0.0000	0.0000	0.0000
LOCC_367	0.8502	0.0966	0.0499	0.0032	0.0000	0.0000	0.0000	0.0001
LOCC_368	0.7524	0.1375	0.0290	0.0021	0.0143	0.0647	0.0000	0.0000
LOCC_369	0.6827	0.2395	0.0521	0.0133	0.0000	0.0110	0.0015	0.0000
LOCC_370	0.8735	0.0506	0.0678	0.0064	0.0000	0.0000	0.0000	0.0016

LOCC_371	0.8588	0.0935	0.0406	0.0005	0.0000	0.0000	0.0067	0.0000
LOCC_372	0.8831	0.1073	0.0094	0.0000	0.0000	0.0000	0.0000	0.0002
LOCC_373	0.8138	0.0577	0.0797	0.0488	0.0000	0.0000	0.0000	0.0000
LOCC_374	0.8273	0.1230	0.0488	0.0000	0.0000	0.0009	0.0000	0.0000
LOCC_375	0.8271	0.0637	0.0819	0.0170	0.0000	0.0103	0.0000	0.0000
LOCC_376	0.8647	0.0913	0.0431	0.0009	0.0000	0.0000	0.0000	0.0000
LOCC_377	0.7810	0.1216	0.0760	0.0150	0.0000	0.0000	0.0063	0.0002
LOCC_378	0.7097	0.1433	0.1124	0.0114	0.0000	0.0232	0.0000	0.0000
LOCC_379	0.9428	0.0249	0.0323	0.0000	0.0000	0.0000	0.0000	0.0000
LOCC_380	0.6987	0.1381	0.1429	0.0057	0.0000	0.0146	0.0000	0.0000
LOCC_381	0.8012	0.0956	0.0686	0.0175	0.0000	0.0171	0.0000	0.0000
LOCC_382	0.9181	0.0593	0.0142	0.0040	0.0000	0.0000	0.0032	0.0012
LOCC_383	0.6594	0.2548	0.0612	0.0095	0.0000	0.0151	0.0000	0.0000
LOCC_384	0.9944	0.0000	0.0000	0.0010	0.0000	0.0000	0.0045	0.0001
LOCC_385	0.8065	0.0630	0.1085	0.0011	0.0000	0.0196	0.0000	0.0014
LOCC_386	0.8383	0.1064	0.0538	0.0015	0.0000	0.0000	0.0000	0.0000
LOCC_387	0.8612	0.0792	0.0338	0.0111	0.0000	0.0000	0.0114	0.0033
LOCC_388	0.9413	0.0217	0.0322	0.0042	0.0000	0.0000	0.0005	0.0000
LOCC_389	0.9290	0.0209	0.0456	0.0000	0.0000	0.0000	0.0045	0.0000
LOCC_390	0.8163	0.0899	0.0505	0.0189	0.0000	0.0245	0.0000	0.0000
LOCC_391	0.7950	0.1053	0.0979	0.0018	0.0000	0.0000	0.0000	0.0000
LOCC_392	0.9116	0.0611	0.0235	0.0033	0.0000	0.0000	0.0000	0.0005
LOCC_393	0.9149	0.0065	0.0666	0.0111	0.0000	0.0009	0.0000	0.0000
LOCC_394	0.9615	0.0365	0.0000	0.0019	0.0000	0.0000	0.0000	0.0002
LOCC_395	0.7795	0.0308	0.1647	0.0250	0.0000	0.0000	0.0000	0.0000
LOCC_396	0.8460	0.0849	0.0590	0.0007	0.0073	0.0019	0.0000	0.0002
LOCC_397	0.8657	0.0807	0.0536	0.0000	0.0000	0.0000	0.0000	0.0000
LOCC_398	0.9119	0.0238	0.0552	0.0010	0.0000	0.0062	0.0000	0.0019
LOCC_399	0.9305	0.0405	0.0261	0.0011	0.0000	0.0000	0.0017	0.0001
LOCC_400	0.8431	0.0938	0.0581	0.0000	0.0000	0.0042	0.0000	0.0008
LOCC_401	0.8907	0.0581	0.0468	0.0039	0.0000	0.0000	0.0005	0.0000
EOCC_average	0.7882	0.1155	0.0675	0.0101	0.0086	0.0080	0.0020	0.0003
LOCC_average	0.8264	0.0909	0.0639	0.0069	0.0039	0.0061	0.0013	0.0004
EOCC vs LOCC P value (One way ANOVA)	< 0.05	NS	NS	NS	NS	NS	NS	NS

EOCC early-onset colon cancer
LOCC late-onset colon cancer
ANOVA analysis or variance
*N*S no significance

Supplementary Table 11. List of WNT signaling downstream target genes.

Gene	Organism/system	Direct/Indirect	Status	References
c-myc	human colon cancer	Yes	Upregulated	He 1998
n-myc	mesenchyme limbs		Upregulated	Ten Berge 2008
Cyclin D	human colon cancer	Yes	Upregulated	Tetsu 1999
				Shtutman 1999
				Disputed by Sansom, 2005
Tcf-1	human colon cancer	Yes	Upregulated	Roose 1999
LEF1	human colon cancer	Yes	Upregulated	Hovanes, 2001
				Filali 2002
PPARdelta	human colon cancer	Yes	Upregulated	He TC, et al 1999
c-jun	human colon cancer	Yes	Upregulated	Mann B, 1999
fra-1	human colon cancer	Yes	Upregulated	Mann B, 1999
uPAR	human colon cancer	?	Upregulated	Mann B, 1999
matrix metalloproteinase MMP-7	human colon cancer	Yes	Upregulated	Brabletz 1999
				Crawford 1999
Axin-2	human colon cancer	Yes	Upregulated	Yan, 2001
				Lustig, 2002
				Jho, 2002
Nr-CAM	human colon cancer	Yes	Upregulated	Conacci-Sorrell 2002
ITF-2	human colon cancer	Yes	Upregulated	Kolligs, 2002
Gastrin	human colon cancer	?	Upregulated	Koh, 2000
CD44	human colon cancer	?	Upregulated	Wielenga 1999
EphB/ephrin-B	human colon cancer	?	Upregulated/Downregulated	Batlle, 2002
BMP4	human colon cancer	?	Upregulated	Kim 2002
claudin-1	human colon cancer	Yes	Upregulated	Miwa 2002
Survivin	human colon cancer		Upregulated	Zhang, 2001
VEGF	human colon cancer	Yes	Upregulated	Zhang, 2001
FGF18	human colon cancer	Yes	Upregulated	Shimokawa 2003
Hath1	human colon cancer	..	Downregulated	Leow 2004
Met	human colon cancer		Upregulated	Boon 2002
endothelin-1	human colon cancer		Upregulated	Kim 2004
c-myc binding protein	human colon cancer	Yes	Upregulated	Jung 2005
L1 neural adhesion	human colon cancer		Upregulated	Gavert 2005
Id2	human colon cancer	Yes	Upregulated	Rockman 2001
				Willert 2002
Jagged	human colon cancer		Upregulated	Rodilla, 2009
Msl1	human colon cancer		Upregulated	Spears, 2011
Tiam1	Colon tumors			Malliri 2005
Nitric Oxide Synthase 2	Hepg2 cells		Upregulated	Du, 2006
Telomerase	ES, other stem	Yes	Upregulated	Hoffmyer, 2012
Dickkopf	Various cells, tumors		Upregulated	Niida. 2004
				Gonzalez-Sancho 2004
				Chamorro 2004
FGF9	ovarian endometrioid adenocarcinoma		Upregulated	Hendrix, 2006LB
LBH	breast cancer		Upregulated	Rieger 2010
FGF20	Various cells, tumors			Chamorro 2004
LGR5/GPR49	Intestine	Yes	Upregulated	Barker, 2007
Sox9	Intestine		Upregulated	Blache 2004
Sox9	mesenchyme		down	Hill, 2005
				Day 2005
				Yano, 2005
Sox17	gastrointestinal tumors		Upregulated	Du, 2009
Runx2	chondrocytes		Upregulated	Dong 2006
Gremlin	fibroblasts		Upregulated	Klapholz-Brown 2007
SALL4				Bohm, 2006
RANK ligand	Osteoblasts		Downregulated	Spencer 2006
Osteoprotegerin	Osteoblasts		Upregulated	Glass, 2005
CCN1/Cyr61	Osteoblasts		Upregulated	Si, 2006
Sox2	Xenopus retina		Upregulated	Van Raay, 2005
Pituitary tumor transforming gene (PTTG)	esophageal squamous cell carcinoma			Zhou 2004
Delta-like 1	somites			Galceran, 2004

				Hofmann 2004
FoxN1	thymus	?	yes	Balciunaite 2002
matrix metalloproteinase-26	Human			Marchenko 2002
nanog	ES			Pereira, 2006
				Cole 2008
44838	ES		Upregulated	Cole 2008
snail	ES/EB		Upregulated	Ten Berge 2008
Fibronectin	ES/EB		Upregulated	Ten Berge 2008
Frizzled 7	EC cells	Yes	Upregulated	Willert 2002
Follistatin	EC cells, ovary	Yes	Upregulated	Willert 2002
				Yao 2004
Wnt3a	EC cells			Zhang 2009
Fibronectin	Mouse lung		Upregulated	De Langhe 2005
Islet1	Cardiac cells		Upregulated	Lin 2007
MMP2, MMP9	T cells			Wu 2007
Siamois	Xenopus	Yes	Upregulated	Brannon 1997
fibronectin	Xenopus	Yes	Upregulated	Gradl 1999
BMP4	Xenopus	?	down	Baker 1999
myogenic bHLH	Xenopus	?	Upregulated	Munsterberg 1995
engrailed-2	Xenopus	Yes	Upregulated	McGrew 1999
Xnr3	Xenopus	Yes	Upregulated	McKendry 1997
connexin43	Xenopus, Mouse	Yes	Upregulated	van der Heyden 1999
twin	Xenopus	Yes	Upregulated	Laurent 1997
connexin 30	Xenopus	?		McGrew 1999
retinoic acid receptor gamma	Xenopus	?		McGrew 1999
dharma/bozozok	Zebrafish	Yes	Upregulated	Ryu 2001
MITF/nacre	Zebrafish	Yes	Upregulated	Dorsky, 2000
				Saito 2002
				Yasumoto 2002
Stra6	Wnt-1 transformed mouse cells	?	Upregulated	Szeto 2001
		co-induced by Wnt plus RA		
Wrch-1	Wnt-1 transformed mouse cells	?	Upregulated	Tao, 2001
		Not through TCF		
TNF family 41BB ligand, ephrinB1, Stra6, autotaxin and ISLR	Wnt-1 transformed mouse cells	By Wnt plus retinoic acid	Upregulated	Tice 2002
Twist	Wnt1 induced mammary cancer		Upregulated	Howe, 2003
Stromelysin	Wnt-1 transformed mouse cells		Upregulated	Prieve, 2003
WISP	Wnt-1 transformed mouse cells	yes, but not through TCF (Xu, 2000)	Upregulated	Xu, 2000
Brachyury (Tbox1)	Mouse (Wnt-3A)	Yes	Upregulated	Yamaguchi 1999
				Arnold 2000
Tbx3 (Tbox3)	Mouse, Human	Yes	Upregulated	Renard 2007
Proglucagon	Mouse	?	Upregulated	Ni 2003
Osteocalcin	Mouse	Yes	Downregulated	Kahler 2003
Cdx1	Mouse embryo			Pilon 2007
cyclooxygenase-2	mouse (Wnt-1)	?	Upregulated	Howe 1999
				Haertel-Wiesmann 2000
Irx3 and Six3	Mouse brain			Braun 2003
neurogenin 1	Mouse brain	Yes	Upregulated	Hirabayashi 2004
SP5	Mouse brain	Yes	Upregulated	Weidinger 2005
				Fujimura 2007
NeuroD1	Mouse Brain	Yes	Upregulated	Kuwabara 2009
Nkx2.2	Neural tube	Yes	Downregulated	Lei, 2006
Gbx2	Neural Crest	Yes	Upregulated	Li, 2009
Cacna1g	Neuron		Upregulated	Wisniewska 2010
WISP-1, WISP-2, IGF-II , Proliferin-2, Proliferin-3, Emp, IGF-I, VEGF-C, MDR1, COX-2, IL-6	3T3-L1 Preadipocytes	?	Upregulated	Longo, 2002
periostin	Mouse Wnt-3	not through b-catenin?	Downregulated	Haertel-Wiesmann 2000
Cdx1	Mouse Wnt-3A	Yes	Upregulated	Lickert 2000
Cdx4	Mouse Wnt-3A			Pilon, 2006
Cdx4	Zebrafish HSC	?	Upregulated	Davidson 2003
betaTrCP		Independent of transcription	Upregulated	Spiegelman 2000
Cdc25	Sarcoma cells	Yes	Upregulated	Viyajakumar 2011
sFRP-2	Mouse (Wnt-4)	?	Upregulated	Lescher 1998
Pitx2	pituitary	Yes	Upregulated	Kioussi 2002

EGF receptor	Liver		Upregulated	Tan 2005
Eda (TNF-related)	Mouse hair follicle	?	Upregulated	Laurikkala 2002
				Durmowicz 2002
E-cadherin	Mouse hair follicle	Yes	Downregulated	Jamora, 2003
E-cadherin	ES/EB		Downregulated	Ten Berge 2008
Keratin	Mouse hair follicle	Yes	Upregulated	Dasgupta 1999
movo1	Mouse hair follicle	Yes	Upregulated	Li, 2002
Jagged1	Mouse hair follicle		Upregulated	Estrach, 2006
P16ink4A	Melanocytes	Yes	Downregulated	Delmas, 2007
CTLA-4	Melanomas	Yes	Upregulated	Shah 2008
mBTEB2	Mouse	Independent β-catenin	Upregulated	Ziemer 2001
FGF4	Mouse tooth bud	Yes	Upregulated	Kratochwil 2002
Interleukin8	Endothelial cells			Masckauchan 2005
ret	rat PC12	?	Upregulated	Zheng, 1996
connexin43	rat cardiomyocytes	?	Upregulated	Ai 2000
versican	vascular smooth muscle cells	Yes	Upregulated	Rahmani 2005
Tnfrsf19	Somitic mesoderm	Yes	Upregulated	Buttitta 2003
Ubx	Drosophila	Yes	Upregulated or Downregulated	Riese 1997
wingless	Drosophila	?	Upregulated or Downregulated	Yu 1998
Dpp	Drosophila	Yes	Downregulated	Yang, 2000
Engrailed	Drosophila	?	Upregulated	Hooper 1994
Dfrizzled2	Drosophila	?	Downregulated	Cadigan 1998
shavenbaby	Drosophila	?	Downregulated	Payre 1999
stripe	Drosophila	Yes	Downregulated	Piepenburg 2000
Nemo	Drosophila		Upregulated	Zeng , 2004

Supplementary Table 12. List of commonly DEGs in PanCK+ and FAP+ or VIM+ in EOCC at the TIM, TC, and AN.

TIM	TC	AN
CEP55	TNFAIP3	IER3
MBNL3	PVRIG	SF3B1
TNFSF9	BCL2L1	CT45A1
AXIN2	RAB7A	PVRIG
CMTM4	RELB	DTX4
ITGB4	MAP3K7	STK11IP
ALKBH3	NFIL3	SSX1
NUF2	SMAD2	KRT5
OPN3	GDF15	TAPBPL
MAP3K8	STAG2	
MAP4K2	PFKFB3	
TCF3	KRAS	
TLK2	GRB7	
MAPK14	NDUFA6	
CD63	LRP6	
SMAD2	SLC7A5	
PLA2G4F	IHH	
NGFR	PRKCA	
PPARG	ULBP2	
GLUD1	MIB1	
DTX4	PLCB4	
COL17A1	MSH2	
GPC4	POLR2A	
IRAK4	L1CAM	
FOXM1	PUM1	
STAT3	MAPK10	
CDC14B	MBNL3	
NFE2L2	REL	
PRC1	TP53	
MELK	ITGA4	
NSD1	NF1	
BRD2	ATR	
STAT2	NFKB1	
LTF	SDHA	
MRPL19	OPN3	
RAD51C	HSP90B1	
KRAS	MAML2	
HSP90B1	CMTM4	
NDUFB4	TTK	
NFIL3	GNAQ	
SMAP1	MAPK14	
HELLS	BST2	
RPTOR	HPRT1	
PDGFC	TLK2	
IL34	RPTOR	
HLA-DRA	FUBP1	
PBX1	ORC6	
WRN	ATP2A2	
SMAD9	NECTIN1	
DLL1	FZD8	
FUT4		
SDHA		
RIPK1		
TMUB2		
UBE2T		
ANP32B		
RNF43		
MAPK10		
EWSR1		
TRAF6		
MIB1		
TAP2		
FGFR2		

MDC1
GDF15
HDAC6
IL20RA
CDC25A
CDK1
HRAS
POLR2A
HSPB1
MAML2
CHEK1
CTSS
SOX9
FANCL
PRKCA
ATG12
MAP3K7
FOXO4
HPRT1
GRB7
MAP2K4
FOXC1
NF1
NECTIN1
HDAC4
COX6A1
ATF2
NDUFB8
RAB7A
ITGB3
SSX1
BLNK
REPS1
RPS6KA6
TP53
POLR2D
MSH6
HLA-DRB
CCRL2
PSEN1
TYMP
FANCB
NOD1
PIAS4
MAP3K5
PRR5
NDUFB11
STAG2
ATP5ME
ULBP2
MFNG
PTPRR
MAP3K20
CD74
SOS2
MAP3K12
EXO1
CASP3
PUM1
ORC6

DEG differentially expressed gene
TIM tumor invasive margin
TC tumor center
AN adjacent normal

Supplementary Table 13. List of antibodies (Ab) and dilutions utilized for muliplex immunofluorescence using Opal Manual Kit

Panel 1: For Validation of FAP expression shown in Supplementary Figure 3

Staining order	Ab name	Class	Type	Dilution	Ab incubation time	Cat #	Supplier	AR	Secondary Ab	Secondary Ab incubation time	Opal Fluorophore	Opal (TSA) dilution	Opal TSA incubation time	TSA Catalog N.
1	PanCK	IgG	Mouse monoclonal	1:200	30 min	M3515	DAKO	AR buffer	Ms Ra	10 min	**690**	1:150	10 min	FP1497A
2	CD45	IgG	Rabbit monoclonal	1:200	1 hr	#13917	Cell Signaling Technology	AR buffer	Ms Ra	10 min	**540**	1:150	10 min	FP1494A
3	FAP	IgG	Rabbit monoclonal	1:200	1 hr	ab240989	Abcam	AR buffer	Ms Ra	10 min	**650**	1:200	10 min	FP1496A
4	DAPI	-	-	1:1000	5 min	62248	ThermoFisher	AR buffer	-	-	-	-	-	-

Panel 2: For Validation of FGF20 expression shown in Figure 6

Staining order	Ab name	Class	Type	Dilution	Ab incubation time	Cat #	Supplier	AR	Secondary Ab	Secondary Ab incubation time	Opal Fluorophore	Opal (TSA) dilution	Opal TSA incubation time	TSA Catalog N.
1	PanCK	IgG	Mouse monoclonal	1:200	30 min	M3515	DAKO	AR buffer	Ms Ra	10 min	**690**	1:150	10 min	FP1497A
2	FGF20	IgG	Rabbit polyclonal	1:25	1 hr	ab198876	Abcam	AR buffer	Ms Ra	10 min	**540**	1:150	10 min	FP1494A
3	FAP	IgG	Rabbit monoclonal	1:200	1 hr	ab240989	Abcam	AR buffer	Ms Ra	10 min	**650**	1:200	10 min	FP1496A
4	DAPI	-	-	1:1000	5 min	62248	ThermoFisher	AR buffer	-	-	-	-	-	-

Panel 3: For Validation of FGFR2 expression shown in Supplementary Figure 13

Staining order	Ab name	Class	Type	Dilution	Ab incubation time	Cat #	Supplier	AR	Secondary Ab	Secondary Ab incubation time	Opal Fluorophore	Opal (TSA) dilution	Opal TSA incubation time	TSA Catalog N.
1	PanCK	IgG	Mouse monoclonal	1:200	30 min	M3515	DAKO	AR buffer	Ms Ra	10 min	**690**	1:150	10 min	FP1497A
2	FGFR2	IgG	Rabbit monoclonal	1:150	1 hr	ab227683	Abcam	AR buffer	Ms Ra	10 min	**620**	1:150	10 min	FP1495A
3	FAP	IgG	Rabbit monoclonal	1:200	1 hr	ab240989	Abcam	AR buffer	Ms Ra	10 min	**650**	1:200	10 min	FP1496A
4	DAPI	-	-	1:1000	5 min	62248	ThermoFisher	AR buffer	-	-	-	-	-	-

Panel 4: For Validation of pAKT expression shown in Figure 8

Staining order	Ab name	Class	Type	Dilution	Ab incubation time	Cat #	Supplier	AR	Secondary Ab	Secondary Ab incubation time	Opal Fluorophore	Opal (TSA) dilution	Opal TSA incubation time	TSA Catalog N.
1	PanCK	IgG	Mouse monoclonal	1:200	30 min	M3515	DAKO	AR buffer	Ms Ra	10 min	**690**	1:150	10 min	FP1497A
2	FGFR2	IgG	Rabbit monoclonal	1:150	1 hr	ab227683	Abcam	AR buffer	Ms Ra	10 min	**620**	1:150	10 min	FP1495A
3	FAP	IgG	Rabbit monoclonal	1:200	1 hr	ab240989	Abcam	AR buffer	Ms Ra	10 min	**650**	1:200	10 min	FP1496A
4	pAKT	IgG	Rabbit polyclonal	1:150	1 hr	#9271S	Cell Signaling Technology	AR buffer	Ms Ra	10 min	**570**	1:150	10 min	FP1488A
5	DAPI	-	-	1:1000	5 min	62248	ThermoFisher	AR buffer	-	-	-	-	-	-

Panel 5: For Validation of FGFR2-FGF20 co-expression shown in Figure 7

Staining order	Ab name	Class	Type	Dilution	Ab incubation time	Cat #	Supplier	AR	Secondary Ab	Secondary Ab incubation time	Opal Fluorophore	Opal (TSA) dilution	Opal TSA incubation time	TSA Catalog N.
1	PanCK	IgG	Mouse monoclonal	1:200	30 min	M3515	DAKO	AR buffer	Ms Ra	10 min	**690**	1:150	10 min	FP1497A
2	FGFR2	IgG	Rabbit monoclonal	1:150	1 hr	ab227683	Abcam	AR buffer	Ms Ra	10 min	**620**	1:150	10 min	FP1495A
3	FGF20	IgG	Rabbit polyclonal	1:100	1hr	ab198876	Abcam	AR buffer	Ms Ra	10 min	**540**	1:150	10 min	FP1494A
4	FAP	IgG	Rabbit monoclonal	1:200	1 hr	ab240989	Abcam	AR buffer	Ms Ra	10 min	**650**	1:150	10 min	FP1496A
5	DAPI			1:1000	5 min	62248	ThermoFisher	AR buffer	-	-	-	-	-	-

Ab Antibody
PanCK Pan-cytokeratin
FAP Fibroblast activation protein
DAPI 4′,6-Diamidino-2-phenylindole dihydrochloride
AR Antigen retrieval
Ms Rb Mouse Rabbit
TSA Tyramide signal amplification
FGFR2 Fibroblast Growth Factor Receptor 2
FGF20 Fibroblast Growth Factor 20
