# Supplementary material for: Spatial profiling of cancer-associated fibroblasts of sporadic early onset colon cancer microenvironment
Source: NPJ Precis Oncol. 2023 Nov 14;7:118. doi: 10.1038/s41698-023-00474-w (PMC10645739; doi:10.1038/s41698-023-00474-w)
Supplement: Supplementary file 2 — REPORTING SUMMARY [file 41698_2023_474_MOESM2_ESM.pdf]

nature portfolio

Corresponding author(s): DAPR NPJPRECISIONONCOLOGY-03518R1

Last updated by author(s): 2023-18-10

Reporting Summary

Nature Portfolio wishes to improve the reproducibility of the work that we publish. This form provides structure for consistency and transparency in reporting. For further information on Nature Portfolio policies, see our Editorial Policies and the Editorial Policy Checklist.

Statistics

For all statistical analyses, confirm that the following items are present in the figure legend, table legend, main text, or Methods section.

n/a	Confirmed	
☐	☒	The exact sample size (*n*) for each experimental group/condition, given as a discrete number and unit of measurement
☐	☒	A statement on whether measurements were taken from distinct samples or whether the same sample was measured repeatedly
☐	☒	The statistical test(s) used AND whether they are one- or two-sided<br>*Only common tests should be described solely by name; describe more complex techniques in the Methods section.*
☒	☐	A description of all covariates tested
☐	☒	A description of any assumptions or corrections, such as tests of normality and adjustment for multiple comparisons
☐	☒	A full description of the statistical parameters including central tendency (e.g. means) or other basic estimates (e.g. regression coefficient) AND variation (e.g. standard deviation) or associated estimates of uncertainty (e.g. confidence intervals)
☒	☐	For null hypothesis testing, the test statistic (e.g. *F*, *t*, *r*) with confidence intervals, effect sizes, degrees of freedom and *P* value noted<br>*Give P values as exact values whenever suitable.*
☒	☐	For Bayesian analysis, information on the choice of priors and Markov chain Monte Carlo settings
☒	☐	For hierarchical and complex designs, identification of the appropriate level for tests and full reporting of outcomes
☒	☐	Estimates of effect sizes (e.g. Cohen's *d*, Pearson's *r*), indicating how they were calculated

Our web collection on statistics for biologists contains articles on many of the points above.

Software and code

Policy information about availability of computer code

Data collection	NicheNet algorithm.
Data analysis	NanoString GeoMx DSP software (NanoString Technologies, Inc., Seattle, WA); ImageJ software (http://imagej.nih.gov/ij/); HTG EdgeSeq RUO Library calculator software version 2.0.0.; Illumina BaseSpace BCL to FASTQ software version 2.2.0 and Illumina Local Run Manager Software version 2.0.0.; HTG EdgeSeq Parser software version v5.1.724.4793; HTG EdgeSeq Reveal (HTG REVEAL software version 2.0.1, http://reveal.htgmolecular.com);Qupath software (v.0.3.2, Queen's University, Belfast, Northern Ireland); InForm Analysis software (v.2.6.0, Akoya Biosciences); GraphPad Prism 7 software (GraphPad software Inc., La Jolla); R 4.2.1. Adobe Illustrator Creative Cloud (Adobe Inc., Los Angeles, CA); CIBERSORTx (https://cibersortx.stanford.edu/); Gene Expression Omnibus (GEO) database (https://www.ncbi.nlm.nih.gov/geo/); Gene set enrich analysis (GSEA) application (Broad Institute of Massachusetts Institute of Technology, https://www.gsea-msigdb.org/gsea/index.jsp).

For manuscripts utilizing custom algorithms or software that are central to the research but not yet described in published literature, software must be made available to editors and reviewers. We strongly encourage code deposition in a community repository (e.g. GitHub). See the Nature Portfolio guidelines for submitting code & software for further information.

Data

Policy information about availability of data

All manuscripts must include a data availability statement. This statement should provide the following information, where applicable:

- Accession codes, unique identifiers, or web links for publicly available datasets
- A description of any restrictions on data availability
- For clinical datasets or third party data, please ensure that the statement adheres to our policy

Data availability
The data that support the findings of this study has been deposited in GEO database under GSE240624 SuperSeries which includes GSE240531 for Nanostring GeoMx DSP dataset and GSE240623 for HTG-EdgeSeq PIP dataset. The rest of the publicly available data utilized and obtained from GEO or TCGA databases have been described in Supplementary Table 1.

Research involving human participants, their data, or biological material

Policy information about studies with human participants or human data. See also policy information about sex, gender (identity/presentation), and sexual orientation and race, ethnicity and racism.

Reporting on sex and gender	We have used the term gender. Gender was determined based on self-reporting by patient in the database.
Reporting on race, ethnicity, or other socially relevant groupings	We have used the race of the patients included based on self-reporting by patient in the in the database.
Population characteristics	The population characteristics included in the study were: Age, Gender, BMI, Race.
Recruitment	This is a retrospective study that analyzed the tissue samples of 26 patients who had elective surgery at SJHC between 2015-2020.
Ethics oversight	Universal Consent (Providence Health and Services Portland IRB: JWCI-18-0401) and Western IRB: MORD-RTPCR-0995.

Note that full information on the approval of the study protocol must also be provided in the manuscript.

Field-specific reporting

Please select the one below that is the best fit for your research. If you are not sure, read the appropriate sections before making your selection.

☒ Life sciences ☐ Behavioural & social sciences ☐ Ecological, evolutionary & environmental sciences

For a reference copy of the document with all sections, see nature.com/documents/nr-reporting-summary-flat.pdf

Life sciences study design

All studies must disclose on these points even when the disclosure is negative.

Sample size	The study included 52 samples from 26 patients diagnosed with sporadic colon cancer. All 26 patients underwent surgery for colon cancer in SJHC between 2015 and 2020. The following data were collected from each patient chart: patient baseline characteristics at the time of operation including, age at diagnosis, gender, race, family history, comorbidities of inflammatory bowel diseases, tumor location, the presence of preoperative treatment, and pathological features of the resected specimens. Patients were divided into young (EOCC, <50 yr.) and old (LOCC, ≥50 yr.) patient groups, according to the definition of EOCC described in previous studies. Patients with any suspicious family history of hereditary colon cancer, a known genetic predisposition for colon cancer, or the comorbidity of inflammatory bowel disease were excluded. Patients who had received any preoperative therapy were also excluded. The location of tumors was defined as right sided (cecum, ascending colon, hepatic flexure, transverse colon, and splenic flexure) or left-sided (descending colon, sigmoid colon), according to the International Classification of Diseases (ICD)-10 classification. The pathological features were determined following the Tumor, Node, and Metastasis (TNM) system, based on the eighth edition of the American Joint Committee on Cancer. DNA MMR status was evaluated by immunohistochemical staining of MLH1, PMS2, MSH2, and MSH6 by the Pathology Dept. at SJHC. Patients with loss of two or more of these four genes were classified as microsatellite instability-high (MSI-H) and excluded from the study. The quality of all FFPE sections was evaluated using hematoxylin and eosin (H&E) staining. All FFPE sections included in the study were evaluated by a board-certified pathologist at the Pathology Dept. at SJHC. Detailed clinicopathological information about the patients is listed in Supplementary Tables 2-4. All the 26 patients included were analyzed by HTG-EdgeSeq PIP (Supplementary Tables 2-3). A cohort of 8 patients, out of the 26 patients included in the study, were assessed by NGDSP. Four patients were diagnosed with EOCC, and four patients were diagnosed with LOCC (Supplementary Table 2). A cohort of 8 patients were assessed by Opal staining (Supplementary Table 2). The patients analyzed by the three assays overlapped (Supplementary Table 2).
Data exclusions	Patients with any suspicious family history of hereditary colon cancer, a known genetic predisposition for colon cancer, or the comorbidity of inflammatory bowel disease were excluded. Patients who had received any preoperative therapy were also excluded.
Replication	The findings on this cohort were validated using publicly available data from TCGA COAD. The TCGA COAD dataset (RNA-Seq) was downloaded

Replication	from UCSC Xena (https://xena.ucsc.edu/). Among 512 tissue samples, 472 samples were successfully annotated with clinical information. Patients with MSI-H or the presence of any preoperative treatment were excluded, resulting in 454 samples for analysis. Samples were categorized into early-onset colon cancer (EOCC, < 50 yr., n = 53) and late-onset colon cancer (LOCC, ≥ 50 yr., n = 401), respectively. A total of 13,145 genes were included in the analysis; genes with max counts < 3 were excluded. Normal tissues from COAD patients who had gene expression profiles (n = 40) were used in different comparisons. The TCGA COAD whole-exome sequencing dataset, to determine gene mutation status, was downloaded from cBioPortal (https://www.cbioportal.org/). The consensus molecular subtyping (CMS) status of the TCGA COAD dataset was downloaded from synapse (http://www.synapse.org).
Randomization	N/A
Blinding	FFPE tissue samples were de-identified, before the analysis. All the bioinformatic analysis were carried out using two groups classification based on the clinical data and the exclusion criteria set.

Reporting for specific materials, systems and methods

We require information from authors about some types of materials, experimental systems and methods used in many studies. Here, indicate whether each material, system or method listed is relevant to your study. If you are not sure if a list item applies to your research, read the appropriate section before selecting a response.

Materials & experimental systems

n/a	Involved in the study	
☐	☒	Antibodies
☐	☒	Eukaryotic cell lines
☒	☐	Palaeontology and archaeology
☒	☐	Animals and other organisms
☐	☒	Clinical data
☒	☐	Dual use research of concern
☒	☐	Plants

Methods

n/a	Involved in the study	
☒	☐	ChIP-seq
☒	☐	Flow cytometry
☒	☐	MRI-based neuroimaging

Antibodies

Antibodies used	This information is provided in Supplementary Tables 6 and 13.
Validation	Validation was performed by manufacturer's.

Eukaryotic cell lines

Policy information about cell lines and Sex and Gender in Research

Cell line source(s)	HT-29: Human Colorectal Adenocarcinoma Cell Line (ATCC HTB-38). This cell line was bougth from ATCC. This cell line was established in 1964 from the primary tumor of a 44-year-old Caucasian female with colorectal adenocarcinoma.
Authentication	Cell line was purchased on 04/06/2021 from ATCC. Cell lines were authenticated by manufacturer's.
Mycoplasma contamination	Cell were tested negative for mycoplasma.
Commonly misidentified lines (See ICLAC register)	N/A

Clinical data

Policy information about clinical studies

All manuscripts should comply with the ICMJE guidelines for publication of clinical research and a completed CONSORT checklist must be included with all submissions.

Clinical trial registration	N/A
Study protocol	N/A
Data collection	N/A
Outcomes	N/A
